# Photophysical, photostability, and ROS generation properties of new trifluoromethylated quinoline-phenol Schiff bases

**DOI:** 10.3762/bjoc.17.191

**Published:** 2021-12-01

**Authors:** Inaiá O Rocha, Yuri G Kappenberg, Wilian C Rosa, Clarissa P Frizzo, Nilo Zanatta, Marcos A P Martins, Isadora Tisoco, Bernardo A Iglesias, Helio G Bonacorso

**Affiliations:** 1 1Núcleo de Química de Heterociclos (NUQUIMHE), Departamento de Química, Universidade Federal de Santa Maria, Santa Maria, RS, 97105-900, Brazil; 2 Laboratório de Bioinorgânica e Materiais Porfirínicos, Departamento de Química, Universidade Federal de Santa Maria, Santa Maria, RS, 97105-900, Brazil

**Keywords:** photophysical properties, photostability, quinoline, ROS generation, Schiff base

## Abstract

A new series of ten examples of Schiff bases, namely (*E*)-2-(((2-alkyl(aryl/heteroaryl)-4-(trifluoromethyl)quinolin-6-yl)imino)methyl)phenols **3**, was easily synthesized with yields of up to 91% from the reactions involving a series of 2-(R-substituted) 6-amino-4-(trifluoromethyl)quinolines **1** and 4(5)-R^1^-substituted salicylaldehydes **2** – in which alkyl/aryl/heteroaryl for 2-R-substituents are Me, Ph, 4-MeC_6_H_4_, 4-FC_6_H_4_, 4-NO_2_C_6_H_4_, and 2-furyl, and R^1^-substituents are 5-NEt_2_, 5-OCH_3_, 4-Br, and 4-NO_2_. Complementarily, the Schiff bases showed low to good quantum fluorescence yield values in CHCl_3_ (Φ_f_ = 0.12–0.80), DMSO (Φ_f_ = 0.20–0.75) and MeOH (Φ_f_ = 0.13–0.85). Higher values of Stokes shifts (SS) were observed in more polar solvents (DMSO; 65–150 nm and MeOH; 65–130 nm) than in CHCl_3_ (59–85 nm). Compounds **3** presented good stability under white-LED irradiation conditions and moderate ROS generation properties were observed.

## Introduction

**Figure 1:**
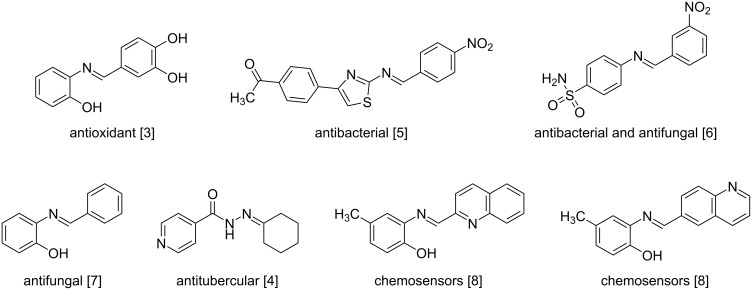
Examples of structures and properties of Schiff bases of interest in the present study.

Schiff bases are an important class of organic compounds first reported by the German chemist Hugo Schiff in 1864 and are formed from the reversible condensation between a primary amine and an aldehyde or a ketone [1]. Also known as azomethines, aldimines, and more commonly as imines, Schiff bases have the general organic function -C=N- [2], and are known to have a wide range of biological activities, including antioxidant [3], antitubercular [4], antibacterial [5], antimicrobial [6], and antifungal properties [7], in addition to their role as chemosensors [8] (Figure 1).

Quinolines are another important class of compounds and have numerous medicinal chemistry applications due to their biological applicability [9] and promising antimycobacterial [10], antimalarial [11–12], and antibacterial [13] activities. On the other hand, 6-aminoquinoline compounds demonstrate interesting luminescent properties [14] that have attracted great interest because of their potential applicability in the composition of organic light-emitting diodes (OLED), organic solar cells (OSC), and biomolecular markers [15–16].

Moreover, the trifluoromethyl substituent (CF_3_) is an interesting electron-withdrawing group that enhances the effects of many bioactive molecules due to a significant improvement in stability, lipophilicity, and resistance to enzymatic degradation [17–18]. Also, it has been widely applied as a special alkyl substituent for ligands of phosphorescent heavy metal complexes in OLEDs. Due to the ability to increase the electron transport and decrease molecule stacking, trifluoromethyl-substituted molecules have been employed to the development of phosphorescent materials [19–21].

On the other hand, antioxidants are known for protecting organisms against cell damage caused by oxidative stress, especially by eliminating reactive oxygen species (ROS) such as hydroxyl radicals (•OH), superoxide anions (O_2_^•‒^), and singlet oxygen (^1^O_2_) [3,22–23]. Therefore, research in recent years has focused on new compounds obtained from natural sources or by synthetic methods, which provide active ingredients to prevent or reduce the effects of oxidative stress in cells.

Recently, our research group reported the synthesis of 6-amino-4-(trifluoromethyl)quinolines, which were obtained through an electrophilic aromatic substitution reaction catalyzed by sulfuric acid from 4-substituted 4-methoxy-1,1,1-trifluoroalk-3-en-2-ones in a two-step reaction procedure and with satisfactory yields of up to 87%. The prepared 6-aminoquinolines presented promising photophysical properties and high thermal stability [14].

**Scheme 1:**
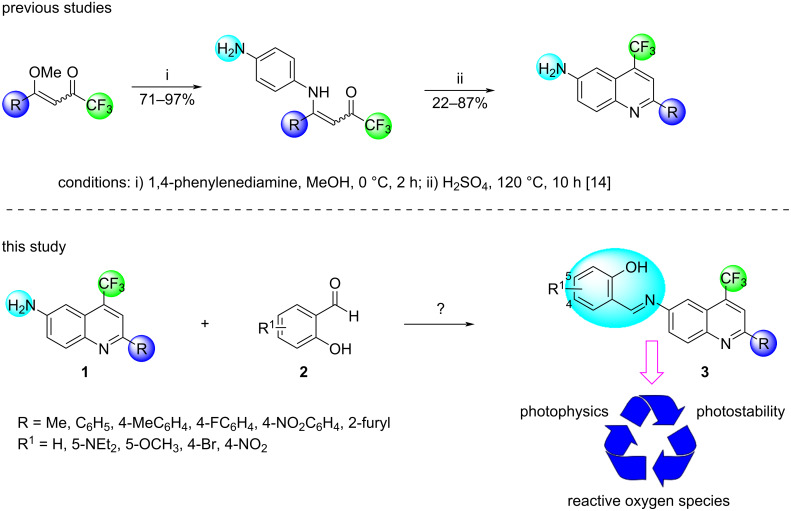
General view for the present study.

In this sense, the present study aimed to synthesize a novel trifluoromethylated hybrid system comprising the Schiff base scaffolds from some 6-aminoquinolines and salicylaldehyde derivatives in order to analyze and evaluate their photophysical, photostability, and antioxidant properties for possible future applications in the pharmacological areas or materials sciences (Scheme 1).

## Results and Discussion

### Chemistry

**Scheme 2:**
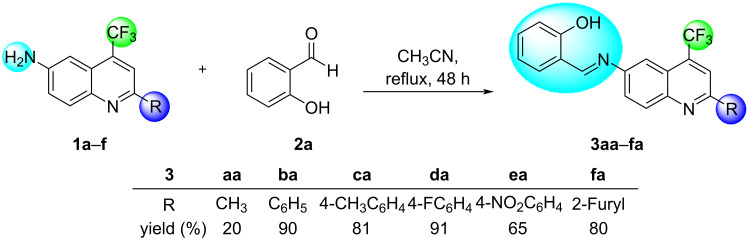
Synthesis of ((trifluoromethyl)quinolinyl)phenol Schiff bases **3aa**–**fa**.

**Scheme 3:**
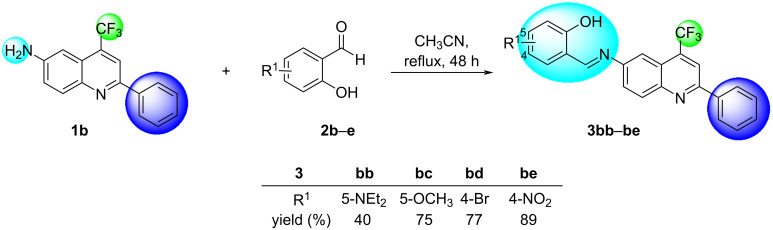
Synthesis of trifluoromethylated quinolinyl-phenol Schiff bases **3bb**–**be**.

The synthetic routes and structures for the synthesis of (*E*)-2-(((2-alkyl(aryl/heteroaryl)-4-(trifluoromethyl)quinolin-6-yl)imino)methyl)phenols **3** are collected in Scheme 2 and Scheme 3.

First, a series of six 6-amino-2-alkyl(aryl/heteroaryl)-4-(trifluoromethyl)quinolines **1a**–**f** was synthesized via an intramolecular cyclization reaction, in which trifluoromethyl-substituted enamino ketones reacted with concentrated sulfuric acid at adequate temperature (120 °C) and time (10 hours) to furnish the desired compounds, following the method already described in the literature by our research group [14].

With the quinolines **1a**–**f** at hands we initially selected quinoline **1b** and salicylaldehyde (**2a**) to optimize the reaction conditions leading to Schiff bases **3**.

Hence, the reaction solvent and molar ratio between the precursors were evaluated. The reactions were carried out using an equimolar ratio of both reactants in methanol, ethanol, and acetonitrile as solvent at reflux temperature according to previously reported data [22,24–25]. Thus, we found increasing yields for **3ba** depending on the solvent used, i.e., 70% (methanol), 80% (ethanol), and 83% (acetonitrile).

Based on these results, we selected acetonitrile as the best solvent for further optimization. When changing the molar ratio of the reactants to a 1:2 molar ratio of quinoline **1b** and salicylaldehyde (**2a**), the yield of the desired product **3ba** increased to 90%. The best result was obtained when quinoline **1b** (1 mmol) was added to the salicylaldehyde (**2a**, 2 mmol) in acetonitrile (10 mL) and refluxing the mixture for 48 h. Under these conditions, the reactions of quinolines **1a**–**f** with salicylaldehyde (**2a**) afforded the desired Schiff bases **3aa**–**fa** (6 examples) with 20–90% yields for the isolated products after recrystallization from ethanol (Scheme 2).

In order to evaluate the properties related to the substituents of the portion provided by salicylaldehyde, the same optimized conditions were applied to the reaction of quinoline **1b** (R = C_6_H_5_) with various salicylaldehydes **2b**–**e**, resulting in four more Schiff bases **3bb**–**be** with 40–89% yield for the isolated products after recrystallization from ethanol (Scheme 3).

With some exceptions, electron-deficient or electron-rich substituted 2-aryl-6-aminoquinolines and aromatic aldehydes worked very well to furnish the Schiff bases **3**. However, a poor yield (20%) was observed when 2-methyl-6-aminoquinoline (**1a**) was employed to obtain the derivative **3aa**. We initially speculated that a competition between the 6-amino group and the 2-methyl substituent present in quinoline **1a** might have occurred, as described by Fu and co-workers [26], which could lead to only the respective 2-alkenylquinoline or to simultaneous reaction products from both moieties (6-amino and 2-methyl substituents). However, at the end of the reaction only the Schiff base **3aa** could be isolated and the 2-alkenylquinoline derivative was not identified by GC or ^1^H NMR.

In contrast to the yield obtained for the synthesis of **3bc** (R^1^ = 5-OMe, 75%), the aromatic aldehyde **2c** substituted with a similar electron-rich group (R^1^ = 5-NEt_2_) gave only a regular 40% yield for **3bb**.

The structures of the new Schiff bases **3** were characterized by ^1^H, ^13^C, and ^19^F NMR spectroscopy and HRMS techniques. The structural assignments for the synthesized quinolines **1a**–**f** were consistent with the ^1^H, ^13^C, and ^19^F NMR spectra described in the literature [14]. When the ^1^H NMR spectral data were recorded in CDCl_3_ as a solvent for the **3aa**–**be** series and compared with the NMR spectral data of the **1a**–**f** series, the lack of the chemical shift related to the signal for the NH_2_ group was clearly noted, which was always present in the series of quinolines **1** at 4.51 ppm, on average. The appearance of a singlet for the azomethine proton (CH=N) with a chemical shift in the 8.53–8.77 ppm region in all ^1^H NMR spectra supported the structures of the Schiff bases **3**. Additionally, the hydrogens of the hydroxy group were observed at 13.12 ppm, on average.

The analysis of the ^13^C NMR spectra in CDCl_3_ for the new Schiff bases **3** showed chemical shifts in the 162.68–164.71 ppm range for the CH=N moiety, which agrees with similar structures described in the literature [7,14,22]. The CF_3_ group bonded at C-4 was assigned as a quartet with ^1^*J*_CF_ ≈ 274.6 Hz, with chemical shifts of 123.52 ppm, on average. The ^19^F NMR spectra in CDCl_3_ showed a singlet at −61.70 ppm, on average, in relation to the CF_3_ group. Furthermore, there were no significant differences in the chemical shift values between the quinoline precursor and new Schiff bases regarding the aromatic resonances in the ^1^H NMR and ^13^C NMR data.

Studies in the literature have reported that the imine group may exist as *E*/*Z* geometrical isomers in the –CH=N double bond [22]. Moreover, the *E* geometrical isomer in the –CH=N- double bond has a higher percentage when in dimethyl sulfoxide-*d*_6_ solution. On the other hand, the *Z* isomer can be stabilized in less polar solvents by an intramolecular hydrogen bond. In the present study, the spectral data were recorded in CDCl_3_ solution and no signal belonging to the *Z* isomer was observed in all cases, which can be confirmed by the chemical shift values in the ^1^H NMR regarding the –CH=N bond.

**Figure 2:**
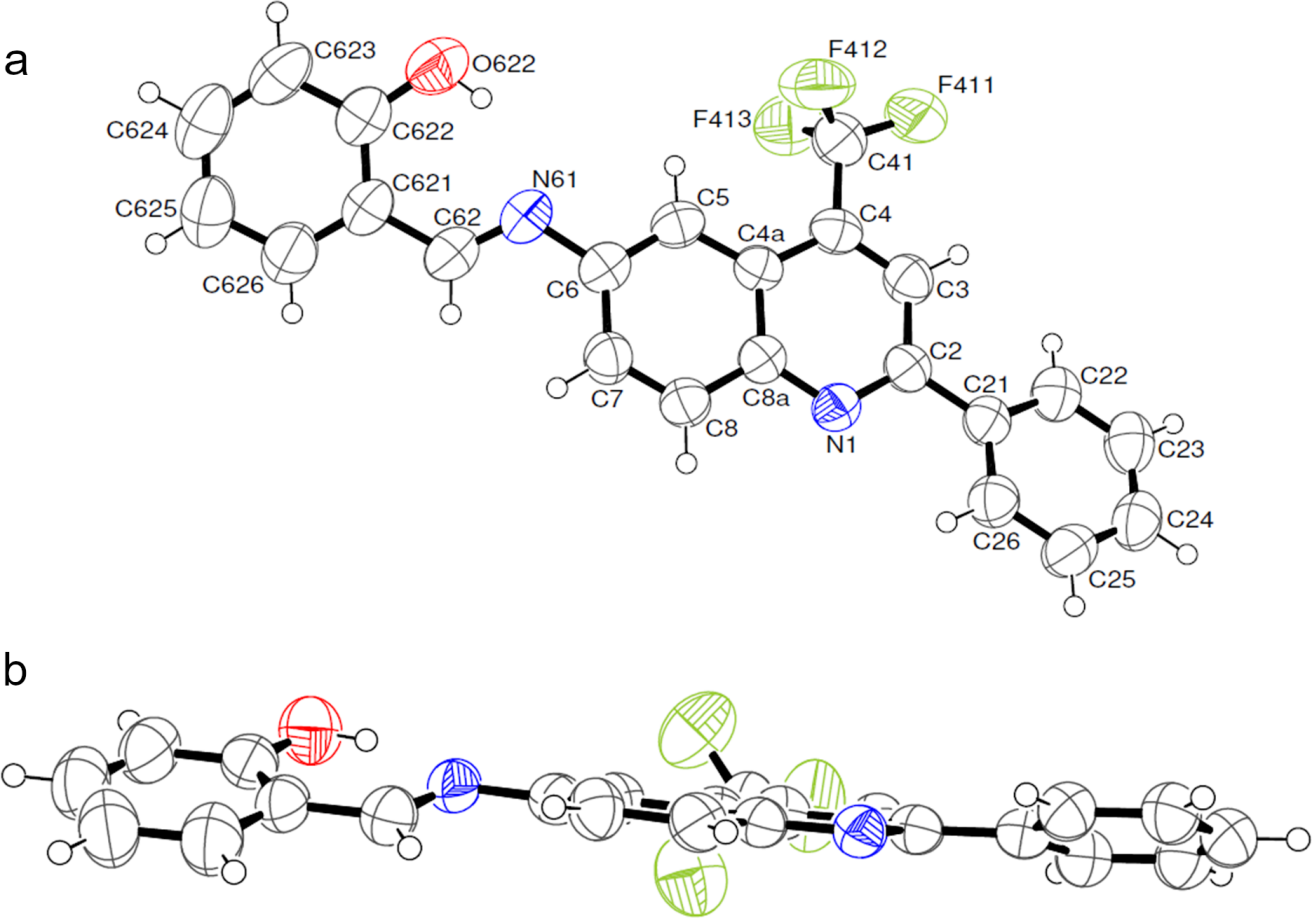
ORTEP diagram of the crystal structure of (*E*)-2-(((2-phenyl-4-(trifluoromethyl)quinolin-6-yl)imino)methyl)phenol (**3ba**, CCDC 2036933). (a) Displacement ellipsoids are drawn at the 50% probability level, and circles with arbitrary radius represent the hydrogen atoms; (b) side view showing coplanarity of the same system.

Finally, in order to determine the real molecular structure of the Schiff bases **3**, single-crystal X-ray diffraction (SC-XRD) was performed for compound **3ba** in the solid state (Figure 2). The compound crystallized in the *P*2_1_/*c* space group, and it was possible to verify that the dihedral angles between the substituent (C_6_H_5_) and quinoline ring (N1–C2–C21–C26) were 18.1°. The dihedral angles between the quinoline ring and the substituent (C_6_H_4_) C(621)–C(62)–N(61)–C(6) were 179.0°, which shows some degree of planarity over the entire molecule. Additional bond lengths and angles and crystallographic refinement details can be found in the Supporting Information File 1 (Tables S1and S2).

### Photophysical behavior

**Figure 3:**
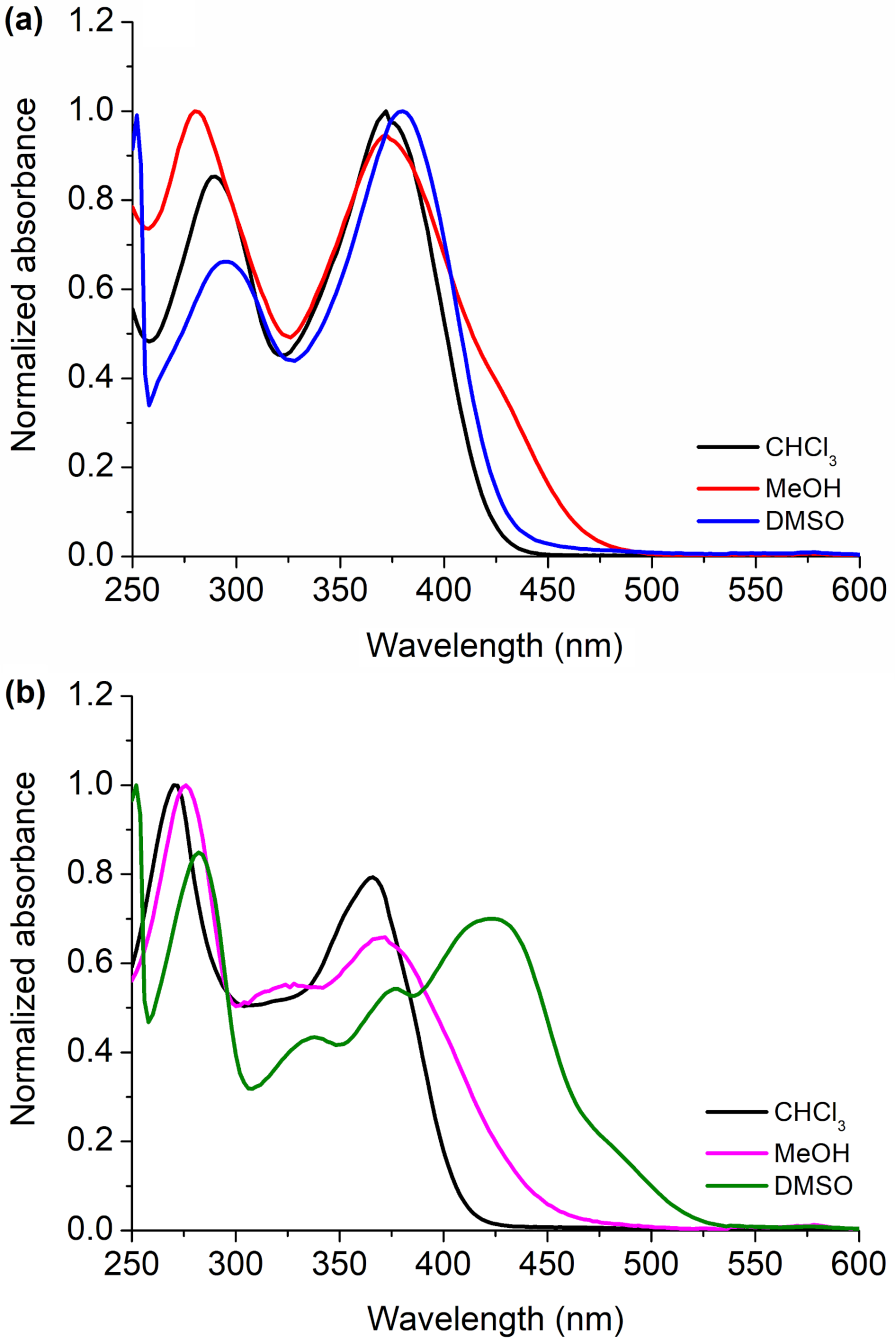
Normalized absorption spectra in the UV–vis region of compounds (a) **3ea** and (b) **3be** in CHCl_3_, MeOH or DMSO solution, respectively ([ ] = 1.50 × 10^−5^ M).

The photophysical study for the series of compounds **3aa**–**fa** and **3bb**–**be** was carried out using chloroform (CHCl_3_), methanol (MeOH) or dimethyl sulfoxide (DMSO) solutions. As comparison, the UV–vis absorption spectra of compounds **3ea** and **3be**, which contain the nitro group in two different ring positions of the molecules recorded in the three solvents are depicted in Figure 3. The values of maximum molar absorption coefficients (in log ε) and wavelengths (nm) of all compounds are listed in Table 1.

**Table 1:** Photophysical data of derivatives **3aa**–**fa** and **3bb**–**be** ([ ] = 1.50 × 10^−5^ M).

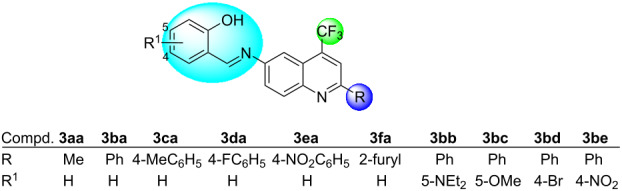				

Compound (CHCl_3_)	λ, nm (log ε)	Emission nm (Φ_f_)^a^	SS (nm/cm^−1^)^b^	*E*_0-0_ (in eV)^c^

**3aa**	275 (4.76), 354 (4.64)	451 (0.90)	97/6075	3.10
**3ba**	274 (4.71), 369 (4.65)	442, 546 (0.71)	73/4475	2.95
**3ca**	276 (4.64), 370 (4.59)	430, 547 (0.70)	60/3770	3.10
**3da**	274 (4.69), 368 (4.64)	438, 544 (0.74)	70/4340	3.00
**3ea**	289 (4.61), 373 (4.67)	439, 556 (0.16)	66/4030	2.90
**3fa**	280 (4.72), 383 (4.71)	451 (0.69)	68/3935	2.90
**3bb**	274 (4.31), 322 (4.26), 420 (4.78)	457 (0.92)	37/1925	2.60
**3bc**	267 (4.53), 293 (4.40), 375 (4.70)	440, 526 (0.65)	65/3940	3.05
**3bd**	273 (4.71), 371 (4.61)	557 (0.81)	186/9000	2.95
**3be**	271 (4.78), 366 (4.68)	438, 524 (0.66)	72/4490	3.00

Compound (DMSO)	λ, nm (log ε)	Emission nm (Φ_f_)^a^	SS (nm/cm^−1^)^b^	*E*_0-0_ (in eV)^c^

**3aa**	277 (4.50), 354 (4.58)	481 (0.96)	127/7460	2.80
**3ba**	276 (4.73), 371 (4.66)	490 (0.94)	119/6545	2.75
**3ca**	278 (4.52), 374 (4.46)	488 (0.95)	114/6245	2.75
**3da**	276 (4.48), 372 (4.42)	491 (0.96)	119/6515	2.75
**3ea**	294 (4.22), 380 (4.42)	488 (0.93)	108/5825	2.75
**3fa**	281 (4.46), 386 (4.55)	491 (0.85)	105/5540	2.70
**3bb**	273 (4.37), 326 (4.15), 431 (4.64)	573 (0.95)	142/5750	2.45
**3bc**	275 (4.34), 296 (4.41), 381 (4.72)	491 (0.96)	110/5880	2.80
**3bd**	275 (4.32), 374 (4.24)	489 (0.89)	115/6290	2.75
**3be**	282 (4.73), 336 (4.39), 376 (4.51), 423 (4.27)	489 (0.80)	66/3190	2.75

Compound (MeOH)	λ, nm (log ε)	Emission nm (Φ_f_)^a^	SS (nm/cm^−1^)^b^	*E*_0-0_ (in eV)^c^

**3aa**	273 (4.35), 356 (4.23)	484 (0.86)	128/7430	2.70
**3ba**	273 (4.27), 372 (4.37)	489 (0.85)	117/6430	2.90
**3ca**	273 (4.65), 367 (4.58)	486 (0.85)	119/6670	2.80
**3da**	275 (4.61), 331 (4.43), 369 (4.39)	490 (0.83)	121/6690	2.80
**3ea**	280 (4.30), 371 (4.28)	485 (0.40)	114/6335	2.90
**3fa**	276 (4.55), 382 (4.54)	488 (0.75)	106/5685	2.77
**3bb**	273 (4.19), 345 (4.63), 427 (4.06)	490 (0.91)	63/3010	2.80
**3bc**	272 (4.25), 371 (4.36)	499 (0.94)	128/6915	2.80
**3bd**	274 (4.43), 371 (4.25)	483 (0.84)	112/6250	2.80
**3be**	275 (4.51), 325 (4.48), 370 (4.32)	490 (0.82)	120/6620	2.75

^a^Using 9,10-diphenylanthracene (DPA) as standard in CHCl_3_ (Φ_f_ = 0.65; λ_exc_ = 366 nm); ^b^SS = Stokes shifts: Δλ = 1/λ_abs_ − 1/λ_em_; ^c^determined from the interception of the normalized absorption and fluorescence emission spectra; *E*_0-0_ = 1240/λ (eV).

The absorption spectra of the studied Schiff base series presented electronic transitions in the 250–500 nm UV–vis region. In the ultraviolet range, the observed transitions can be attributed to the π → π* transitions and refer to the heterocyclic ring. Transitions above 350 nm can be attributed to the *n* → π* transition referring to the imine moiety, causing an intramolecular charge-transfer type (ICT) transition [27]. Complementary, according to Temel and co-workers who studied a similar scaffold, namely, 4-bromo-2-((quinoline-8-yl)methyl)phenol [27], no imine–hemiaminal tautomer peak transition was observed for the Schiff bases **3**.

In general, there were slightly significant changes in the transitions according to the changes in the substituent or polarity of the solvent (behavior in CHCl_3_, MeOH, and DMSO is quite similar). Notably, one can highlight compound **3bb** (R = Ph, R^1^ = 5-NEt_2_) which transitions vary significantly with the change in polarity of the medium (4–24 nm), and this may be due to the donor diethylamino group attached to the imine portion of the molecule. In comparison to the absorption spectra of aminoquinolines published by Kappenberg and co-workers [14], the Schiff bases studied here show similar absorption behavior, making it clear that these transitions originate mainly from the substituents (NH_2_, CF_3_, and R) attached to the quinoline heterocycle ring, respectively.

Moreover, as shown in Figure 3, the position of the substituent in the molecule (as exemplarily shown for compounds **3ea** and **3be**) in the same solvent and in solvents of different polarities affect the values of the wavelengths and molar absorption coefficients of the derivatives studied here. All absorption spectra are listed in Supporting Information File 1 (Figures S2–S7).

**Figure 4:**
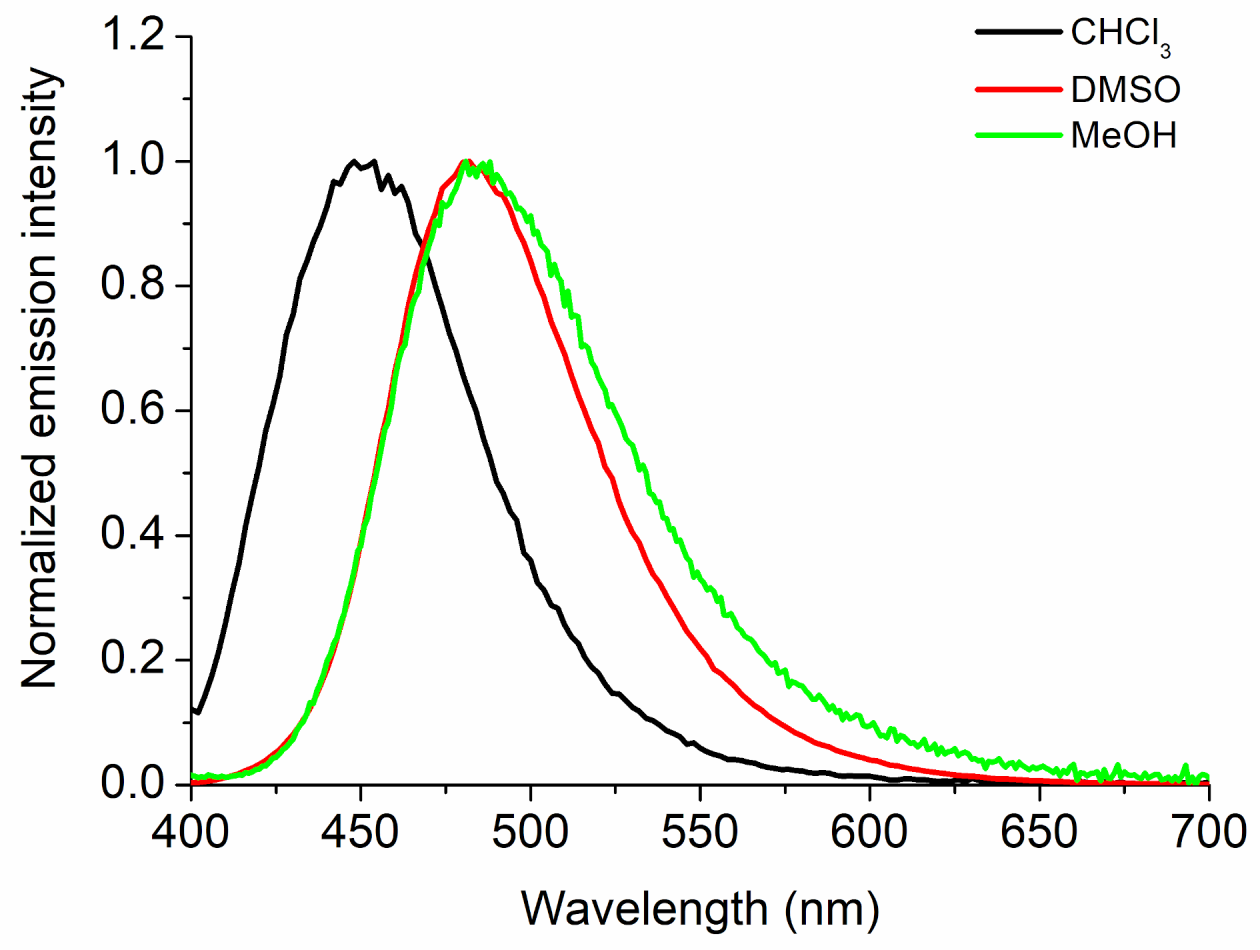
Normalized steady-state fluorescence emission spectra of compound **3aa** (R = Ph, R^1^ = H) in CHCl_3_ (black solid line), DMSO (red solid line) and MeOH (light green solid line) solutions ([ ] = 1.50 × 10^−5^ M).

The steady-state fluorescence emission spectra of the Schiff base compounds from their absorption in the UV–vis region was carried out. Derivatives **3aa**–**fa** and **3bb**–**be** were analyzed in CHCl_3_, DMSO, and MeOH solutions by the emission and excitation spectra and exemplified in the Supporting Information File 1 (Figures S2–S7). For emission measurements, the maximum wavelength with the lowest absorption energy was used for excitation for the fluorescence measurements. The quantum fluorescence yields (Φ_f_) were calculated in order to prove the quantum efficiency of these derivatives in terms of fluorescence emission and thus discuss the influence of the different solvents and substituents. Firstly, by comparing the selected solvents, compound **3aa** (R = Ph, R^1^ = H) presented an emission in the 400–550 nm region depending on the solvent polarity (CHCl_3_, DMSO, and MeOH). Therefore, it is possible to infer that the polarity of the solvents directly influences the electronic transitions in the excited state, with significant changes for the compound in more polar and protic solvents, by shifting the emission maxima to lower energy values (Figure 4).

**Figure 5:**
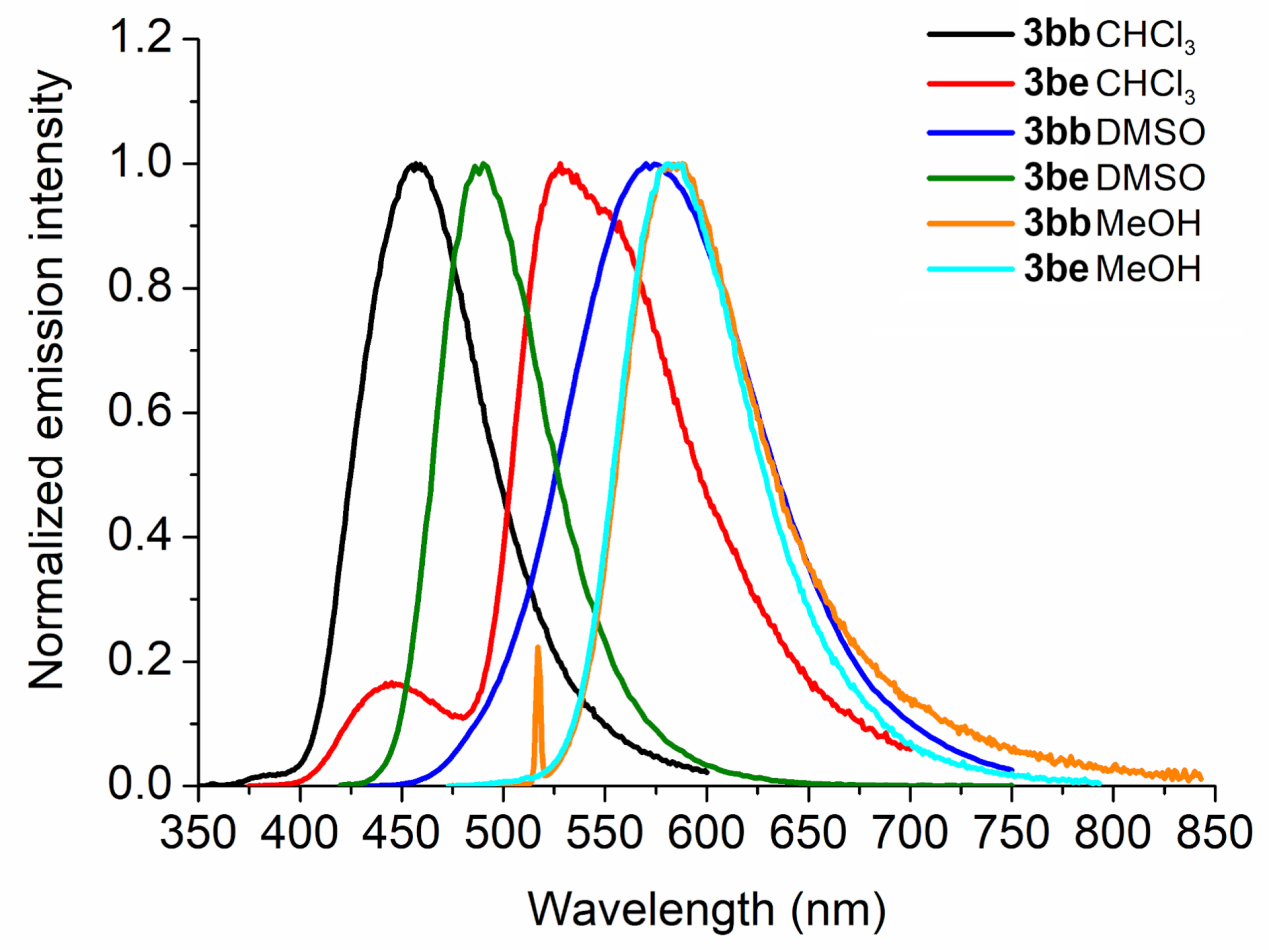
Comparative normalized steady-state fluorescence emission spectra of compounds **3bb** and **3be** in the three studied solvents ([ ] = 1.50 × 10^−5^ M).

Another possible comparison is between substituents with electron-donating and accepting properties in the same solvent media. Therefore, when the compounds containing a diethylamino group (**3bb**, R = Ph, R^1^ = 5-NEt_2_) and a nitro group (**3be**, R = Ph, R^1^ = 4-NO_2_) were analyzed, a significant difference was observed compared to the other compounds in the series **3**. Thus, we can say that these characteristics exist because there is more stabilization of the excited state in a polar environment (DMSO and MeOH), adding to a possible push–pull effect of the diethylamino group (donor group) [14,28–29]. Moreover, these results should indicate a negative possibility of excited state intramolecular proton transfer (ESIPT) phenomena occurring (in protic MeOH solvent and low values of Stokes shifts), being only a direct influence of the substituents on the excited state (Figure 5 and Table 1) [30–31].

In general, the Schiff bases showed variable quantum fluorescence yields in CHCl_3_, DMSO and MeOH solutions (see Table 1), using 9,10-diphenylanthracene (DPA) as standard molecule by comparative method. An analysis of the obtained Φ_f_ values revealed significant differences, mainly according to the change in solvent polarity, being, for example, higher for compounds in DMSO and MeOH than in chloroform solution. Furthermore, the highest values were found in the presence of electron-donating groups, i.e., in compounds **3bb** (R = Ph, R^1^ = 5-NEt_2_) and **3bc** (R = Ph, R^1^ = 5-OMe) (Table 1). These values observed according to the solvent may be related to the stabilization of the excited state in each solution.

Regarding the Stokes shifts (SS), higher values were observed for derivatives in a more polar solvent (DMSO and MeOH) than in CHCl_3_ (Table 1) and also depending on the electronic properties of the substituents in the molecules. According to the characteristics and properties described herein, once again, we can attribute these differences to the stabilization of the excited states in more polar solvents combined with the properties of electron-donating groups (push–pull system).

As in the aminoquinolines described in the literature [14], the Schiff base derivatives present emission spectra in a different region to the amino derivatives (blue to green region), but with higher quantum fluorescence yields and Stokes shifts, a fact attributed to a greater electronic conjugation provided by the imine function present in the molecules of the series **3**.

### Photostability and singlet oxygen quantum yield (Φ_Δ_) assays

**Figure 6:**
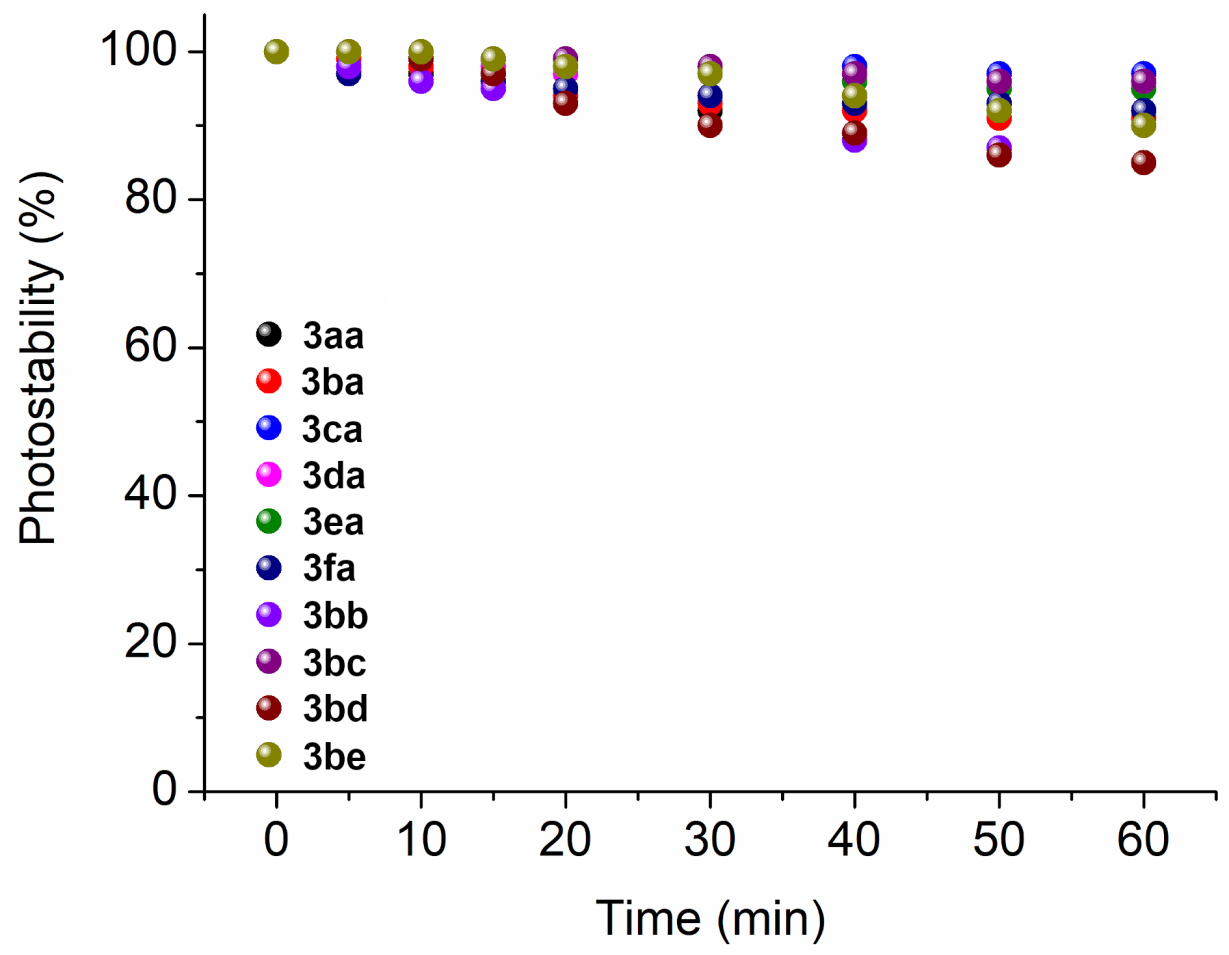
Photostability (%) plots of derivatives **3aa**–**fa** and **3bb**–**be** in DMSO solution after irradiation with white-light LED array system (400–800 nm) at a fluence rate of 25 mW/cm^2^ for different periods (0 to 60 min; total light dosage = 90 J/cm^2^).

In order to be efficient for applications in photobiology, organic dyes must be stable when subjected to light irradiation for long periods of time. Thus, the photostability parameter is an important characteristic considering that photogenerated singlet oxygen (^1^O_2_) can react with a dye molecule, promoting its degradation [32]. Based on the changes in the UV–vis absorption spectra as a function of time, the Schiff base derivatives **3aa**–**fa** and **3bb**–**be** were confirmed to present good photostability under white-light LED irradiation conditions (25 mW/cm^2^ fluence rate and 90 J/cm^2^ light dosage) in the 400–800 nm range for 60 min in DMSO solution (Figure 6).

The ability of the Schiff bases **3aa**–**fa** and **3bb**–**be** to produce ^1^O_2_ species was studied in DMSO solution using a 1,3-diphenylisobenzofuran (DPBF) photooxidation assay and the adequate equation as described by Pivetta and co-workers [33]. In this study, the methylene blue dye (MB) was used as a reference. The photooxidation rate constants (*k*) and ^1^O_2_ yield of derivatives (Φ_Δ_) were determined and the values are presented in Table 2. All UV–vis spectra of the DPBF photooxidation are shown in Supporting Information File 1 (Figures S8–S17).

**Table 2:** Photooxidation rate constants and singlet oxygen quantum yield of compounds **3aa**–**fa** and **3bb**–**be** in DMSO solution.

Compound	R	R^1^	*k* (min^−1^)	Φ_Δ_	Compound	R	R^1^	*k* (min^−1^)	Φ_Δ_

**3aa**	Me	H	2.6 × 10^−4^	0.19	**3bb**	Ph	5-NEt_2_	7.0 × 10^−4^	0.51
**3ba**	Ph	H	2.2 × 10^−4^	0.16	**3bc**	Ph	5-OMe	2.5 × 10^−4^	0.18
**3ca**	4-MeC_6_H_4_	H	1.0 × 10^−4^	0.07	**3bd**	Ph	4-Br	1.6 × 10^−4^	0.12
**3da**	4-FC_6_H_4_	H	5.4 × 10^−4^	0.40	**3be**	Ph	4-NO_2_	1.3 × 10^−4^	0.09
**3ea**	4-NO_2_C_6_H_4_	H	1.9 × 10^−4^	0.14	MB^a^	–	–	7.7 × 10^−4^	0.52
**3fa**	2-Furyl	H	2.3 × 10^−4^	0.17	RhB^b^	–	–	6.3 × 10^−4^	0.78

^a^MB = methylene blue dye standard in ethanol solution (Φ_Δ_ = 0.52) [34]. ^b^RhB = rhodamine B dye in ethanol solution (Φ_Δ_ = 0.78) [35].

**Figure 7:**
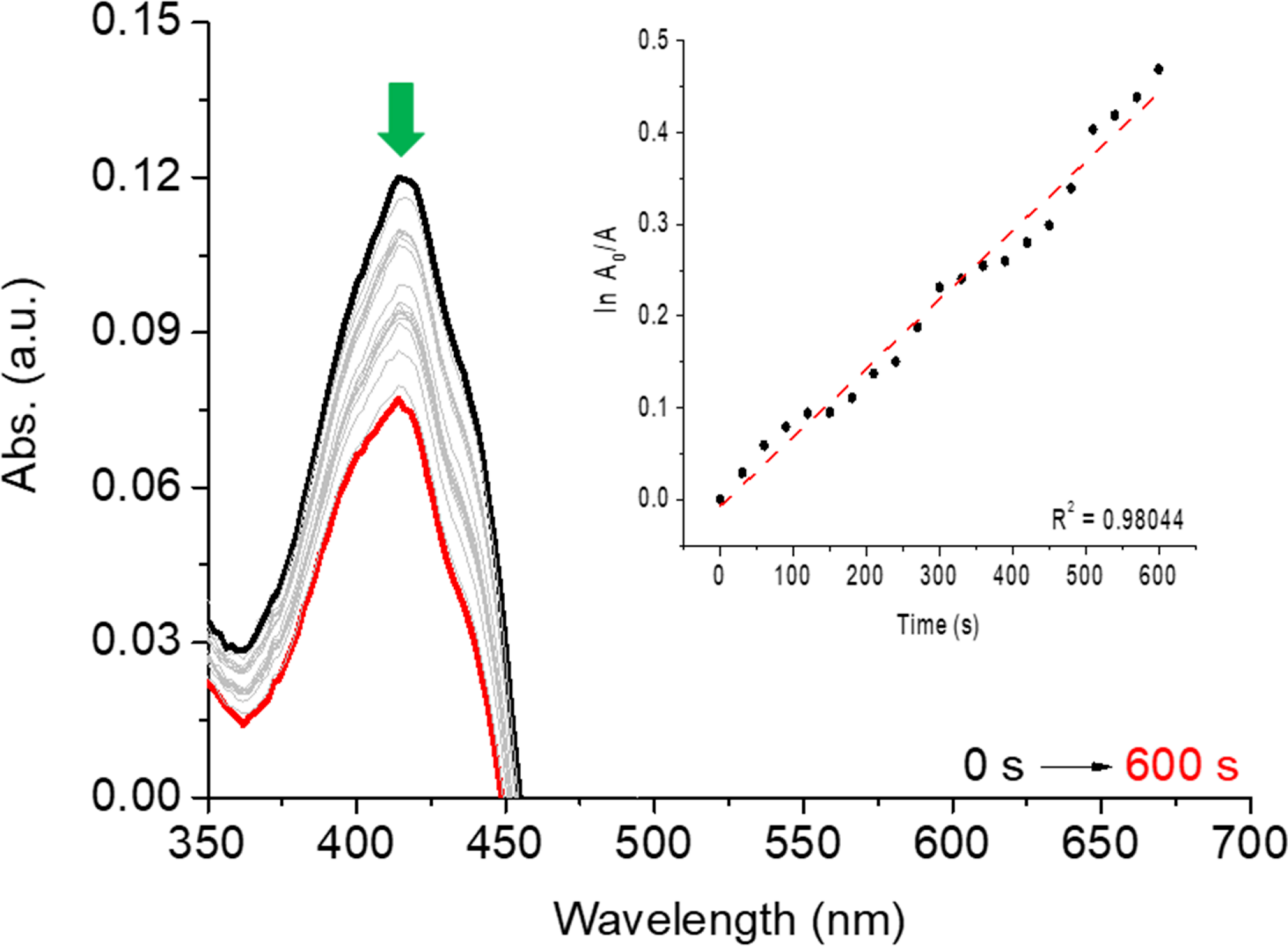
DPBF photooxidation assays by red-light irradiation with diode laser (λ = 660 nm) in the presence of quinoline **3bb** (R = Ph, R^1^ = 5-NEt_2_). The inset shows the first-order kinetic profile.

In general, all compounds at a concentration of 0.5 μM showed weak photooxidization tendency against DPBF quencher at 50 μM (e.g., **3bb**; Figure 7). In this way, Schiff bases presented moderate singlet oxygen species generator character (Φ_Δ_ between 0.07–0.51) after 600 s of irradiation with a red-light diode laser source (λ = 660 nm, 100 mW).

The moderate generation of singlet oxygen species may be attributed to the formation of other possible reactive oxygen species in DMSO solution (e.g., hydroxyl and superoxide radical species) that are not identified by the type of photooxidation assay employed in this work. The good photostability and tendency of these Schiff base derivatives to generate ^1^O_2_ under light irradiation demonstrated here highlights their potential as dyes in photobiological applications.

## Conclusion

In conclusion, we reported the synthesis and photophysical and antioxidant properties of a new series of ten novel Schiff bases **3**. The scaffolds **3** could be synthesized by a simple condensation reaction between 6-aminoquinolines and salicylaldehydes and an easy purification methodology with yields of up to 91%. Photophysical experiments with the derivatives exhibited common transitions in these heterocycle units and corroborated the aromatic structures and good fluorescence quantum yield values for all compounds. Additionally, good photostability and moderate ROS generation may be interesting features for applying these derivatives in photooxidation and photodamage reactions to biomolecules.

## Experimental

### General

Unless otherwise indicated, all reagents and solvents were used as obtained from commercial suppliers and without further purification. ^1^H and ^13^C NMR spectra were acquired on a Bruker DPX 400 MHz spectrometer for one-dimensional experiments and on a Bruker Avance III 600 MHz for ^19^F NMR spectra and 2D-experiments (gHMBC). NMR spectra were recorded using 5 mm sample tubes, at 298 K, at a digital resolution of ±0.01 ppm in CDCl_3_ with TMS as the internal reference. All results are reported as follows: Chemical shift (δ) (multiplicity, coupling constant, integration). The following abbreviations were used to explain multiplicities: s = singlet, bs = broad singlet, d = doublet, t = triplet, q = quartet, qui = quintet, m = multiplet, dd = doublet of doublets. All NMR chemical shifts are reported in parts per million relative to the internal reference. All melting points were determined using coverslips on a Microquímica MQAPF-302 apparatus. FTIR spectra were recorded using the ATR sampling mode on a Bruker VERTEX 70 spectrophotometer with Platinum ATR accessory (diamond crystal) in the 4000–400 cm^−1^ region. High-resolution mass spectra (HRMS) were obtained for all compounds on a hybrid high-resolution and high-accuracy (5 μL/L) micro Q-TOF mass spectrometer (Bruker Scientific^®^, Billerica, MA, USA) at the Caxias do Sul University – UCS, Brazil. The found HRMS values were within 0.003 *m*/*z* unit of the calculated values, as following: **3aa** (0.001 *m*/*z*), **3ba** (0.002 *m*/*z*), **3ca** (0.0001 *m*/*z*), **3da** (0.0004 *m*/*z*), **3ea** (0.0001 *m*/*z*), **3fa** (0.0005 *m*/*z*), **3bb** (0.003 *m*/*z*), **3bc** (0.003 *m*/*z*), **3bd** (0.0000 *m*/*z*) and **3be** (0.0003 *m*/*z*). CHN elemental analyses were performed using a Perkin Elmer CHNS/O Analyzer 2400 Series II at the Federal University of Rio Grande do Sul – UFRGS and the values obtained for carbon, hydrogen, and nitrogen agreed with the calculated values within 0.5%. All NMR and FTIR spectra can be found in Supporting Information File 1 (Figures S38–S47). Single crystals of compound **3ba** were obtained by slow evaporation of a CDCl_3_ solution at 25 °C. Diffraction measurement of compound **3ba** was performed using a Bruker D8 QUEST diffractometer using Cu Kα radiation (λ = 1.54178 Å) with a KAPPA four-circle goniometer equipped with a PHOTON II CPAD area detector, at a temperature of 296 K. For spectroscopic analysis, UV–vis absorption spectra were recorded using a Shimadzu UV2600 spectrophotometer (2.0 nm data range), using DMSO, MeOH or chloroform as solvent. The steady-state emission fluorescence spectra in DMSO, MeOH or chloroform solutions were measured with a Cary50 Eclipse Fluorescence Spectrophotometer (excitation/emission; slit 2.5 mm). All spectra can be found in Supporting Information File 1 (Figures S2–S7). Photostability assays were performed using white-light LED array system irradiation (visible range) at 25 mW/cm^2^ and total light dosage 90 J/cm^2^ at 60 min, according to the current literature. All experiments were performed in duplicate and independently. In order to measure ^1^O_2_ generation ability, UV–vis spectra of the solutions (samples and standard) were recorded for different exposure times by using a 660 nm red light diode laser positioned 2.0 cm from the sample (TheraLase DMC, São Carlos, SP, Brazil) with an average power of 100 mW, during 10 min (irradiation intervals every 30 s). All spectra are provided in Supporting Information File 1 (Figures S8–S17).

### Synthetic procedures

#### General procedure for the preparation of Schiff bases **3aa–af** and **3bb–be**

A mixture of the respective 6-amino-2-alkyl(aryl/heteroaryl)-4-(trifluoromethyl)quinoline (**1a**–**f**, 1.0 mmol) and salicylaldehyde (**2a**–**e**, 2.0 mmol) in anhydrous acetonitrile (10.0 mL) was heated for 48 h at reflux temperature. After completion of the reaction (TLC) and cooling the mixture to room temperature, the solid was filtered under reduced pressure. The crude compounds **3** were purified by recrystallization from ethanol to provide the desired (*E*)-2-(((4-(trifluoromethyl)quinolin-6-yl)imino)methyl) phenols **3** in 20–91% yield.

#### Spectral data

(*E*)-2-(((2-Methyl-4-(trifluoromethyl)quinolin-6-yl)imino)methyl)phenol (**3aa**): Yellow solid, yield 20%; mp 129–132 °C; ^1^H NMR (400 MHz, CDCl_3_) δ 12.98 (s, 1H, OH), 8.72 (s, 1H, CH=N), 8.15 (d, *J* = 8.9 Hz, 1H, H-8), 7.85 (bs, *J* = 2.1 Hz, 1H, H-5), 7.74 (dd, *J* = 9.0, 2.3 Hz, 1H, H-7), 7.61 (s, 1H, H-3), 7.48–7.39 (m, 2H, C_6_H_4_OH), 7.09–7.03 (m, 1H, C_6_H_4_OH), 6.98 (td, *J* = 7.5, 1.1 Hz, 1H, C_6_H_4_OH), 2.82 (s, 3H, CH_3_) ppm; ^13^C NMR (100 MHz, CDCl_3_) δ 164.11 (CH=N), 161.18 (C_6_H_4_OH), 158.14 (C-2), 147.63 (C-8a), 147.20 (C-6), 134.40 (q, *J* = 31.6 Hz, C-4), 133.76 (C_6_H_4_OH), 132.67 (C_6_H_4_OH), 130.94 (C-8), 124.85 (C-7), 123.36 (q, *J* = 274.0 Hz, CF_3_), 121.85 (C-4a), 119.64 (q, *J* = 5.3 Hz, C-3), 119.29 (C_6_H_4_OH), 119.01 (C_6_H_4_OH), 117.37 (C_6_H_4_OH), 114.75 (t, *J* = 2.2 Hz, C-5), 25.34 (CH_3_) ppm; ^19^F NMR (565 MHz, CDCl_3_) δ −61,71 (CF_3_) ppm; FTIR (ATR) ν: 3061 (ν OH), 1627 (ν CH=N), 1118 (ν C-O) cm^−1^; HRMS–ESI (*m/z*): [M + H]^+^ calcd for C_18_H_14_F_3_N_2_O, 331.1053; found, 331.1037.

(*E*)-2-(((2-Phenyl-4-(trifluoromethyl)quinolin-6-yl)imino)methyl)phenol (**3ba**): Yellow solid, yield 90%; mp 183–186 °C; ^1^H NMR (600 MHz, CDCl_3_) δ 12.96 (s, 1H, OH), 8.71 (s, 1H, CH=N), 8.27 (d, *J* = 8.9 Hz, 1H, H-8), 8.20–8.14 (m, 3H, Ph, H-3), 7.87 (bs, 1H, H-5), 7.74 (dd, *J* = 8.8, 2.3 Hz, 1H, H-7), 7.54 (t, *J* = 7.3 Hz, 2H, Ph), 7.49 (t, *J* = 7.1 Hz, 1H, Ph), 7.45–7.38 (m, 2H, C_6_H_4_OH), 7.05 (d, *J* = 8.2 Hz, 1H, C_6_H_4_OH), 6.96 (t, *J* = 7.4 Hz, 1H, C_6_H_4_OH) ppm; ^13^C NMR (151 MHz, CDCl_3_) δ 164.09 (CH=N), 161.23 (C_6_H_4_OH), 156.13 (C-2), 148.02 (C-8a), 147.58 (C-6), 138.09 (Ph), 134.80 (q, *J* = 31.6 Hz, C-4), 133.81 (C_6_H_4_OH), 132.71 (C_6_H_4_OH), 131.96 (C-8), 130.11 (Ph), 129.03 (Ph), 127.36 (Ph), 125.08 (C-7), 123.50 (q, *J* = 274.9 Hz, CF_3_), 122.44 (C-C4a), 119.29 (C_6_H_4_OH), 119.01 (C_6_H_4_OH), 117.38 (C_6_H_4_OH), 116.48 (q, *J* = 5.1 Hz, C-3), 114.71 (C-5) ppm; ^19^F NMR (565 MHz, CDCl_3_) δ −61.62 (CF_3_); FTIR (ATR) ν: 3057 (ν OH), 1625 (ν CH=N), 1029 (ν C-O) cm^−1^; HRMS–ESI (*m/z*): [M + Na]^+^ calcd for C_23_H_15_F_3_N_2_NaO, 415.1029; found, 415.1007.

(*E*)-2-(((2-(*p*-Tolyl)-4-(trifluoromethyl)quinolin-6-yl)imino)methyl)phenol (**3ca**): Yellow solid, yield 81%; mp 210–213 °C; ^1^H NMR (600 MHz, CDCl_3_) δ 12.98 (s, 1H, OH), 8.72 (s, 1H, CH=N), 8.25 (d, *J* = 8.8 Hz, 2H, H-8), 8.15 (s, 1H, H-3), 8.09 (d, *J* = 7.82 Hz, 2H, 4-tolyl), 7.86 (bs, 2H, H-5), 7.74 (d, *J* = 9.1 Hz, 2H, H-7), 7.46–7.40 (m, 2H, 4-tolyl), 7.34 (d, *J* = 7.8 Hz, 2H, C_6_H_4_OH), 7.06 (d, *J* = 8.3 Hz, 1H, C_6_H_4_OH), 6.97 (t, *J* = 7.4 Hz, 2H, C_6_H_4_OH), 2.44 (s, 3H, H-CH_3_); ^13^C NMR (151 MHz, CDCl_3_) δ 164.04 (CH=N), 161.29 (C_6_H_4_OH), 156.18 (C-2), 148.10 (C-8a), 147.44 (C-6), 140.47 (4-CH_3_-C_6_H_4_), 135.38 (4-tolyl), 134.75 (q, *J* = 31.5 Hz, C-4), 133.82 (C_6_H_4_OH), 132.74 (C_6_H_4_OH), 131.92 (C-8), 129.83 (4-tolyl), 127.30 (4-tolyl), 125.04 (C-7), 123.59 (q, *J* = 274.7 Hz, CF_3_), 122.37 (C-4a), 119.34 (C_6_H_4_OH), 119.10 (C_6_H_4_OH), 117.44 (C_6_H_4_OH), 116.40 (q, *J* = 7.3 Hz, C-3), 114.80 (C-5), 21.42 (4-*C*H_3_C_6_H_4_); ^19^F NMR (565 MHz, CDCl_3_) δ −61.65 (CF_3_); FTIR (ATR) ν: 3035 (ν OH), 1621 (ν CH=N), 1112 (ν C-O) cm^−1^; HRMS–ESI (*m/z*): [M + H]^+^ calcd for C_24_H_17_F_3_N_2_O, 407.1366; found, 407.1365.

(*E*)-2-(((2-(4-Fluorophenyl)-4-(trifluoromethyl)quinolin-6-yl)imino)methyl)phenol (**3da**): Yellow solid, yield 91%; mp 188–189 °C; ^1^H NMR (600 MHz, CDCl_3_) δ 12.91 (s, 1H, OH), 8.74 (s, 1H, CH=N), 8.27 (d, *J* = 9.0 Hz, 1H, H-8), 8.23–8.18 (m, 2H, 4-FC_6_H_4_), 8.14 (S, 1H, H-3), 7.89 (p, *J* = 2.0 Hz, 1H, H-5), 7.76 (dd, *J* = 8.9, 2.3 Hz, 1H, H-7), 7.49–7.42 (m, 2H, 4-FC_6_H_4_), 7.29–7.21 (m, 2H, C_6_H_4_OH), 7.09 (dd, *J* = 8.3, 1.1 Hz, 1H, C_6_H_4_OH), 7.00 (td, *J* = 7.5, 1.1 Hz, 1H, C_6_H_4_OH) ppm; ^13^C NMR (151 MHz, CDCl_3_) δ 164.25 (d, *J* = 250.8 Hz, 4-FC_6_H_4_), 164.13 (CH=N), 161.33 (C_6_H_4_OH), 154.97 (C-2), 148.03 (C-8a), 147.74 (C-6), 134.99 (q, *J* = 31.6 Hz, C-4), 134.31 (d, *J* = 3.6 Hz, 4-FC_6_H_4_), 133.86 (C_6_H_4_OH), 132.72 (C_6_H_4_OH), 131.92 (C-8), 129.32 (d, *J* = 8.7 Hz, 4-F-C_6_H_4_), 125.22 (C-7), 123.51 (q, *J* = 274.3 Hz, CF_3_), 122.39 (C-4a), 119.31 (C_6_H_4_OH), 119.07 (C_6_H_4_OH), 117.44 (C_6_H_4_OH), 116.17–115.85 (m) (3, 4-FC_6_H_4_), 114.67 (d, *J* = 2.2 Hz, C-5) ppm; ^19^F NMR (565 MHz, CDCl_3_) δ −61.67 (CF_3_), −110.77 (4-FC_6_H_4_) ppm; FTIR (ATR) ν: 3076 (ν OH), 1629 (ν CH=N), 1011 (ν C-O) cm^−1^; HRMS–ESI) (*m/z*): [M + H]^+^ calcd for C_23_H_14_F_4_N_2_O, 411.1115; found, 411.1119; Anal. calcd for C_23_H_14_F_4_N_2_O: C, 67.32; H, 3.44; N, 6.83; found: C, 67.18; H, 3.06; N, 6.97.

(*E*)-2-(((2-(4-Nitrophenyl)-4-(trifluoromethyl)quinolin-6-yl)imino)methyl)phenol (**3ea**): Orange solid, yield 65%; mp 223–226 °C; ^1^H NMR (600 MHz, CDCl_3_) δ 12.85 (s, 1H, OH), 8.77 (s, 1H, CH=N), 8.41 (s, 4H, 4-NO_2_C_6_H_4_), 8.34 (d, *J* = 8.7 Hz, 1H, H-8), 8.24 (s, 1H, H-3), 7.91 (s, 1H, H-5), 7.84 (dd, *J* = 8.9, 2.3 Hz, 1H, H-7), 7.53–7.41 (m, 2H, C_6_H_4_OH), 7.08 (d, *J* = 8.3 Hz, 1H, C_6_H_4_OH), 7.01 (t, *J* = 7.4 Hz, 1H, C_6_H_4_OH) ppm; ^13^C NMR (100 MHz, CDCl_3_) δ 164.71 (CH=N), 161.29 (C_6_H_4_OH), 153.41 (C-2), 148.72 (d, *J* = 3.6 Hz, C-8a, C-6), 148.03 (4-NO_2_C_6_H_4_), 143.81 (4-NO_2_C_6_H_4_), 135.45 (q, *J* = 32.4 Hz, C-4), 134.14 (C_6_H_4_OH), 132.87 (C_6_H_4_OH), 132.30 (C-8), 128.23 (4-NO_2_C_6_H_4_), 125.92 (C-7), 124.24 (4-NO_2_C_6_H_4_), 123.31 (q, *J* = 275.0 Hz, CF_3_), 123.08 (C-4a), 119.46 (C_6_H_4_OH), 118.96 (C_6_H_4_OH), 117.48 (C_6_H_4_OH), 116.33 (q, *J* = 5.2 Hz, C-3), 114.64 (C-5) ppm; ^19^F NMR (565 MHz, CDCl_3_) δ −61.66 (CF_3_); FTIR (ATR) ν: 3085 (ν OH), 1625 (ν CH=N), 1113 (ν C-O) cm^−1^; HRMS–ESI (*m/z*): [M + H]^+^ calcd for C_23_H_15_F_3_N_3_O_3_, 438.1060; found, 438.1059.

(*E*)-2-(((2-(Furan-2-yl)-4-(trifluoromethyl)quinolin-6-yl)imino)methyl)phenol (**3fa**): Brown solid, yield 80%; mp 205–209 °C; ^1^H NMR (600 MHz, CDCl_3_) δ 12.95 (s, 1H, OH), 8.74 (s, 1H, CH=N), 8.23 (dd, *J* = 9.0, 0.5 Hz, 1H, H-8), 8.13 (d, *J* = 0.8 Hz, 1H, H-3), 7.85 (p, *J* = 2.0 Hz, 1H, H-5), 7.75 (dd, *J* = 9.0, 2.3 Hz, 1H, H-7), 7.66 (dd, ^3^*J* = 1.7, ^4^*J* = 0.8 Hz, 1H, H-5” furyl), 7.49–7.39 (m, 2H, C_6_H_4_OH), 7.30 (dd, ^3^*J* = 3.5, ^4^*J* = 0.8 Hz, 1H, H-3” furyl), 7.08–7.04 (m, 1H, C_6_H_4_OH), 6.98 (td, *J* = 7.5, 1.1 Hz, 1H, C_6_H_4_OH), 6.63 (dd, ^3^*J* = 3.5, ^3^*J* = 1.8 Hz, 1H, H-4” furyl) ppm; ^13^C NMR (151 MHz, CDCl_3_) δ 164.11 (CH=N), 161.28 (C_6_H_4_OH), 152.75 (C-2), 147.97 (d, *J* = 3.7 Hz, C-6, C-8a), 147.52 (furyl), 144.82 (furyl), 134.96 (t, *J* = 31.9 Hz, C-4), 133.88 (C_6_H_4_OH), 132.76 (C_6_H_4_OH), 131.58 (C-8), 125.31 (C-7), 123.37 (q, *J* = 275.0 Hz, CF_3_), 122.38 (C-4a), 119.37 (C_6_H_4_OH), 119.08 (C_6_H_4_OH), 117.44 (C_6_H_4_OH), 115.40 (q, *J* = 5.6 Hz, C-3), 114.96 (d, *J* = 3.1 Hz, C-5), 112.67 (furyl), 111.28 (furyl) ppm; ^19^F NMR (565 MHz, CDCl_3_) δ −61.82 (CF_3_) ppm; FTIR (ATR) ν: 3066 (ν OH), 1612 (ν CH=N), 1121 (ν C-O) cm^−1^; HRMS–ESI (*m/z*): [M + H]^+^ calcd for C_21_H_13_F_3_N_2_O_2_, 383.1002; found, 383.0997.

(*E*)-5-(Diethylamino)-2-(((2-phenyl-4-(trifluoromethyl)quinolin-6-yl)imino)methyl)phenol (**3bb**): Orange solid, yield 40%; mp 208–210 °C; ^1^H NMR (600 MHz, CDCl_3_) δ 13.43 (s, 1H, OH), 8.53 (s, 1H, CH=N), 8.22 (d, *J* = 9.0 Hz, 1H, H-8), 8.18 (d, *J* = 7.4 Hz, 2H, Ph), 8.14 (s, 1H, H-3), 7.81 (p, *J* = 2.2 Hz, 1H, H-5), 7.72 (dd, *J* = 9.0, 2.3 Hz, 1H, H-7), 7.55–7.51 (m, 2H, Ph), 7.49–7.45 (m, 1H, Ph), 7.22–7.19 (m, 1H, 5-NEt_2_-C_6_H_3_OH), 6.27 (dd, *J* = 8.8, 2.5 Hz, 1H, 5-NEt_2_C_6_H_3_OH), 6.22 (d, *J* = 2.4 Hz, 1H, 5-NEt_2_C_6_H_3_OH), 3.41 (q, *J* = 7.1 Hz, 4H, (N-(C*H*_2_CH_3_)_2_), 1.22 (t, *J* = 7.1 Hz, 6H, (N-(CH_2_C*H*_3_)_2_) ppm; ^13^C NMR (151 MHz, CDCl_3_) δ 164.11 (CH=N), 161.79 (5-NEt_2_C_6_H_3_OH), 155.33 (C-2), 152.43 (5-NEt_2_C_6_H_3_OH), 148.59 (C-8a), 147.69 (C-6), 138.50 (Ph), 134.48 (q, *J* = 31.1 Hz, C-4), 134.26 (5-NEt_2_C_6_H_3_OH), 131.74 (C-8), 129.83 (Ph), 128.98 (Ph), 127.33 (Ph), 125.69 (C-7), 123.75 (q, *J* = 274.7 Hz, CF_3_), 122.81 (C-4a), 116.24 (q, *J* = 5.6, 5.2 Hz, C-3), 113.45 (C-5), 109.37 (5-NEt_2_C_6_H_3_OH), 104.26 (5-NEt_2_C_6_H_3_OH), 97.87 (5-NEt_2_C_6_H_3_OH), 44.66 ((N-(CH_2_CH_3_)_2_), 12.72 (N-(CH_2_CH_3_)_2_) ppm; ^19^F NMR (565 MHz, CDCl_3_) δ −61.73 (CF_3_) ppm; FTIR (ATR) ν: 2974 (ν OH), 1638 (ν CH=N), 1117 (ν C-O) cm^−1^; HRMS–ESI (*m/z*): [M + Na]^+^ calcd for C_27_H_24_F_3_N_3_NaO, 486.1764; found, 486.1727; Anal. calcd for C_27_H_24_F_3_N_3_O: C, 69.97; H, 5.22; N, 9.07; found: C, 69.50; H, 5.09; N, 9.26.

(*E*)-5-Methoxy-2-(((2-phenyl-4-(trifluoromethyl)quinolin-6-yl)imino)methyl)phenol (**3bc**): Orange solid, yield 75%; mp 226–230 °C; ^1^H NMR (600 MHz, CDCl_3_) δ 13.34 (s, 1H, OH), 8.64 (s, 1H, CH=N), 8.26 (d, *J* = 8.9 Hz, 1H, H-8), 8.23–8.11 (m, 3H, 2H, Ph, H-3), 7.85 (bs, 1H, H-5), 7.76–7.69 (m, 1H, H-7), 7.52 (dt, *J* = 14.2, 7.1 Hz, 3H, Ph), 7.33 (d, *J* = 8.4 Hz, 1H, 5-OCH_3_C_6_H_3_OH), 6.53 (d, *J* = 8.8 Hz, 2H, 5-OCH_3_C_6_H_3_OH), 3.86 (s, 3H, OCH_3_) ppm; ^13^C NMR (151 MHz, CDCl_3_) δ 164.70 (CH=N), 164.03 (5-OCH_3_C_6_H_3_OH), 162.99 (5-OCH_3_C_6_H_3_OH), 155.99 (C-2), 148.01 (d, *J* = 3.8 Hz, C-6, C-8a), 138.38 (Ph), 134.82 (d, *J* = 31.5 Hz, C-4), 134.05 (5-OCH_3_C_6_H_3_OH), 132.01 (C-8), 130.05 (Ph), 129.05 (Ph), 127.42 (Ph), 125.35 (C-7), 123.69 (q, *J* = 274.7 Hz, CF_3_), 122.68 (C-4a), 116.46 (q, *J* = 4.9 Hz, C-3), 114.26 (C-5), 113.23 (5-OCH_3_C_6_H_3_OH), 107.60 (5-OCH_3_C_6_H_3_OH), 101.28 (5-OCH_3_C_6_H_3_OH), 55.52 (OCH_3_) ppm; ^19^F NMR (565 MHz, CDCl_3_) δ −61.74 (CF_3_) ppm; FTIR (ATR) ν: 3016 (ν OH), 1628 (ν CH=N), 1000 (ν C-O) cm^−1^; HRMS–ESI (*m/z*): [M + H]^+^ calcd for C_24_H_17_F_3_N_2_O_2_, 423.1315; found, 423.1284.

(*E*)-4-Bromo-2-(((2-phenyl-4-(trifluoromethyl)quinolin-6-yl)imino)methyl)phenol (**3bd**): Orange solid, yield 77%; mp 194–196 °C; ^1^H NMR (600 MHz, CDCl_3_) δ 12.86 (s, 1H, OH), 8.63 (s, 1H, CH=N), 8.27 (d, *J* = 9.0 Hz, 1H, H-8), 8.19 (d, *J* = 7.0 Hz, 3H, 2H Ph, H-3), 7.89–7.84 (m, 1H, H-5), 7.72 (dd, *J* = 9.0, 2.3 Hz, 1H, H-7), 7.57–7.44 (m, 5H, 3H, Ph, 2H, 4-BrC_6_H_3_OH), 6.94 (d, *J* = 8.8 Hz, 1H, 4-BrC_6_H_3_OH) ppm; ^13^C NMR (151 MHz, CDCl_3_) δ 162.68 (CH=N), 160.34 (4-BrC_6_H_3_OH), 156.48 (C-2), 148.32 (C-8a), 147.18 (C-6), 138.15 (Ph), 136.39 (4-BrC_6_H_3_OH), 135.02 (q, *J* = 31.5 Hz, C-4), 134.64 (4-BrC_6_H_3_OH), 132.22 (C-8), 130.22 (Ph), 129.06 (Ph), 127.44 (Ph), 124.89 (C-7), 123.59 (q, *J* = 274.3 Hz, CF_3_), 122.49 (C-4a), 120.49 (4-BrC_6_H_3_OH), 119.45 (4-BrC_6_H_3_OH), 116.60 (q, *J* = 5.4 Hz, C-3), 114.92 (d, *J* = 2.4 Hz, C-5), 110.80 (4-BrC_6_H_3_OH) ppm; ^19^F NMR (565 MHz, CDCl_3_) δ −61.63 (CF_3_) ppm; FTIR (ATR) ν: 3060 (ν OH), 1602 (ν CH=N), 1112 (ν C-O) cm^−1^; HRMS–ESI (*m/z*): [M + H]^+^ calcd for C_23_H_14_BrF_3_N_2_O, 471.0314; found, 471.0314; Anal. calcd for C_23_H_14_BrF_3_N_2_O: C, 58.62; H, 2.99; N, 5.94; found: C, 58.04; H, 2.73; N, 6.26.

(*E*)-4-Nitro-2-(((2-phenyl-4-(trifluoromethyl)quinolin-6-yl)imino)methyl)phenol (**3be**): Orange solid, yield 89%; mp 253–256 °C; ^1^H NMR (600 MHz, CDCl_3_) δ 14.01 (s, 1H, OH), 8.85 (s, 1H, CH=N), 8.48 (d, *J* = 2.7 Hz, 1H, 4-NO_2_C_6_H_3_OH), 8.35 (d, *J* = 9.0 Hz, 1H, H-8), 8.33–8.30 (m, 1H, 4-NO_2_C_6_H_3_OH), 8.25–8.19 (m, 3H, H-3, Ph), 7.95 (bs, 1H, H-5), 7.81 (dd, *J* = 8.9, 2.3 Hz, 1H, H-7), 7.58 (t, *J* = 7.3 Hz, 2H, Ph), 7.53 (t, *J* = 7.2 Hz, 1H, Ph), 7.16 (d, *J* = 9.1 Hz, 1H, 4-NO_2_C_6_H_3_OH) ppm; ^13^C NMR (151 MHz, CDCl_3_) δ 166.49 (CH=N), 162.31 (4-NO_2_C_6_H_3_OH), 156.92 (C-2), 148.46 (C-8a), 146.05 (C-6), 140.26 (4-NO_2_C_6_H_3_OH), 137.97 (Ph), 135.14 (q, *J* = 30.5 Hz, C-4), 132.44 (C-8), 130.39 (Ph), 129.14 (Ph), 128.86 (4-NO_2_C_6_H_3_OH), 128.74 (4-NO_2_C_6_H_3_OH), 127.47 (Ph), 124.52 (C-7), 123.43 (q, *J* = 274.9 Hz, C-CF_3_), 122.42 (C-4a), 118.45 (4-NO_2_C_6_H_3_OH), 118.11 (4-NO_2_C_6_H_3_OH), 116.86 (q, *J* = 5.4 Hz, C-3), 115.55 (C-5) ppm; ^19^F NMR (565 MHz, CDCl_3_) δ −61.57 (CF_3_); FTIR (ATR) ν: 3072 (ν OH), 1602 (ν CH=N), 1125 (ν C-O) cm^−1^; HRMS–ESI (*m/z*): [M + H]^+^ calcd for C_23_H_14_F_3_N_3_O_3_, 438.1060; found, 438.1063.

## Supporting Information

File 1 NMR spectra of the compounds, IR spectra, crystallographic data, photophysical and singlet oxygen spectra of new structures.
