# Supplementary material for: Photophysical, photostability, and ROS generation properties of new trifluoromethylated quinoline-phenol Schiff bases
Source: Beilstein J Org Chem. 2021 Dec 1;17:2799–811. doi: 10.3762/bjoc.17.191 (PMC8649202; doi:10.3762/bjoc.17.191)
Supplement: File 1 — NMR spectra of the compounds, IR spectra, crystallographic data, photophysical and singlet oxygen spectra of new structures. [file Beilstein_J_Org_Chem-17-2799-s001.pdf]

BEILSTEIN JOURNAL OF ORGANIC CHEMISTRY

Supporting Information

for

Photophysical, photostability, and ROS generation properties of new trifluoromethylated quinoline-phenol Schiff bases

Inaiá O. Rocha, Yuri G. Kappenberg, Wilian C. Rosa, Clarissa P. Frizzo, Nilo Zanatta, Marcos A. P. Martins, Isadora Tisoco, Bernardo A. Iglesias and Helio G. Bonacorso

Beilstein J. Org. Chem. **2021,** *17,* 2799–2811. doi:10.3762/bjoc.17.191

NMR spectra of the compounds, IR spectra, crystallographic data, photophysical and singlet oxygen spectra of new structures

License and Terms: This is a supporting information file under the terms of the Creative Commons Attribution License (https://creativecommons.org/licenses/by/4.0). Please note that the reuse, redistribution and reproduction in particular requires that the author(s) and source are credited and that individual graphics may be subject to special legal provisions.
The license is subject to the *Beilstein Journal of Organic Chemistry* terms and conditions: (https://www.beilstein-journals.org/bjoc/terms)

Table of contents

1. Crystallographic data S1

2. Photophysical analyses and photostability assays S8

3. Singlet oxygen quantum yield (Φ_Δ) measurements S12

4. ^{1}H, ^{13}C, and ^{19}F NMR spectra S18

5. FTIR spectra S33

References S38

1. Crystallographic data

Single crystals of compound **3ba** were obtained by slow evaporation of a $CDCl_3$ solution at 25 °C. Diffraction measurement of compound **3ba** was performed using a Bruker D8 QUEST diffractometer using CuKα radiation (λ = 1.54178 Å) with a KAPPA four-circle goniometer equipped with a PHOTON II CPAD area detector, at a temperature of 296 K. Absorption corrections were performed using semi-empirical from equivalents methods. Non-hydrogen atoms were refined using anisotropic displacement parameters. The positions of the hydrogen atoms were calculated for idealized positions. The structure was solved and refined using the WinGX software package [1]. The structures were refined based on the full-matrix least-squares method using the SHELXL program [2]. The ORTEP projections of the molecular structures were generated using the ORTEP-3 program [1]. Crystallographic information file (CIF) for the novel structure was deposited at the Cambridge Crystallographic Data Centre (CCDC) under identification number 2036933 (**3ba**). The crystallographic data are collected in Figure S1 and Table S1 and S2).

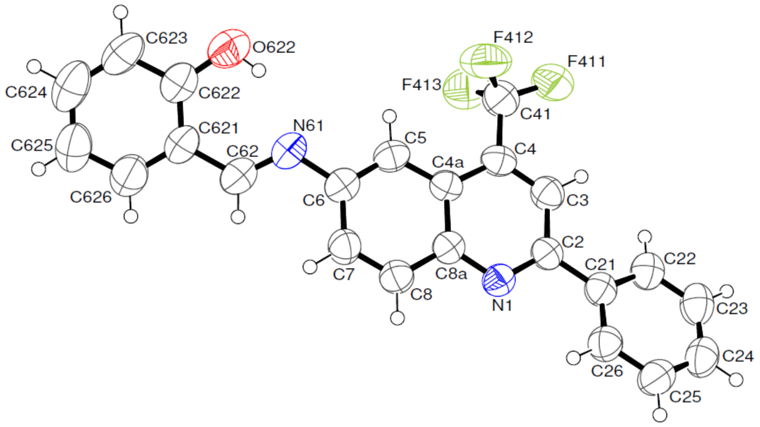

Figure S1: ORTEP of the Schiff base (**3ba**, CCDC 2036933).

checkCIF/PLATON report

You have not supplied any structure factors. As a result the full set of tests cannot be run.

THIS REPORT IS FOR GUIDANCE ONLY. IF USED AS PART OF A REVIEW PROCEDURE FOR PUBLICATION, IT SHOULD NOT REPLACE THE EXPERTISE OF AN EXPERIENCED CRYSTALLOGRAPHIC REFEREE.

No syntax errors found. CIF dictionary Interpreting this report

Datablock: shelx

```
Bond precision:   C-C = 0.0045 A          Wavelength=1.54178

Cell:             a=4.9442(2)     b=25.5275(12)      c=14.6982(7)
                  alpha=90        beta=98.687(3)     gamma=90
Temperature:      296 K
```

	Calculated	Reported
Volume	1833.82(14)	1833.82(14)
Space group	P 21/c	P 21/c
Hall group	-P 2ybc	-P 2ybc
Moiety formula	C23 H15 F3 N2 O	C23 H14 N2 O1 F3
Sum formula	C23 H15 F3 N2 O	C23 H15 F3 N2 O
Mr	392.37	392.37
Dx,g cm-3	1.421	1.421
Z	4	4
Mu (mm-1)	0.921	0.921
F000	808.0	808.0
F000'	810.85	
h,k,lmax	5,30,17	5,30,17
Nref	3348	3154
Tmin,Tmax	0.898,0.946	0.635,0.753
Tmin'	0.773	

```
Correction method= # Reported T Limits: Tmin=0.635 Tmax=0.753
AbsCorr = MULTI-SCAN

Data completeness= 0.942          Theta(max)= 68.384

R(reflections)= 0.0563( 1592)      wR2(reflections)= 0.1512( 3154)

S = 1.012                  Npar= 262
```

The following ALERTS were generated. Each ALERT has the format
test-name_ALERT_alert-type_alert-level.
Click on the hyperlinks for more details of the test.

Table S1: Crystal data and structure refinement for compound **3ba**.

Compound	**3ba**
CCDC number	2036933
Empirical formula	$C_{23}H_{15}F_3N_2O$
Molecular weight	392.37
Temperature (K)	296(2)
Wavelength (Å)	1.54178
Crystal system	Monoclinic
Space group	$P2_1/c$
Cell parameters	
a (Å)	4.9442(2)
b (Å)	25.5275(12)
c (Å)	14.6982(7)
α (°)	90
β (°)	98.687(3)
γ (°)	90
Volume ($Å^3$)	1833.82(14)
Z	4
Calculated density (Mg/m^3)	1.421
Abs. coef. (mm^{-1})	0.921
F (000)	808
Crystal size (mm)	0.279 x 0.097 x 0.060
θ range for data collection (°)	3.500 to 68.384
h, k, l range	-5<=h<=5, -30<=k<=30, -17<=l<=17
Reflections collected / unique	11117 / 3154 [R(int) = 0.0685]

Completeness to theta (%)	94.2
Absorption correction	Semi-empirical from equivalents
Max. and min. transmission	0.7531 and 0.6354
Refinement method	Full-matrix least-squares on F^2
Data / restraints / parameters	3154 / 0 / 262
Goodness-of-fit on F^2	1.012
Final R indices	R1 = 0.0563, wR2 = 0.1175
R all data	R1 = 0.1375, wR2 = 0.1512
Extinction coefficient	None
$\Delta\rho_{máx.}$ and $\Delta\rho_{min}$ (e A^{-3})	0.149 and -0.169

Table S2: Bond lengths [A] and angles [°] for compound **3ba**

F(412)-C(41)	1.337(4)	C(622)-C(621)-C(62)	121.7(3)
F(413)-C(41)	1.329(4)	N(61)-C(62)-C(621)	123.5(3)
F(411)-C(41)	1.323(4)	N(61)-C(62)-H(62)	118.2
O(622)-C(622)	1.351(4)	C(621)-C(62)-H(62)	118.2
O(622)-H(622)	0.8200	C(5)-C(6)-C(7)	120.3(3)
N(61)-C(62)	1.272(4)	C(5)-C(6)-N(61)	116.7(3)
N(61)-C(6)	1.427(4)	C(7)-C(6)-N(61)	123.1(3)
N(1)-C(2)	1.318(3)	C(6)-C(5)-C(4A)	121.3(3)
N(1)-C(8A)	1.364(3)	C(6)-C(5)-H(5)	119.4
C(624)-C(623)	1.362(5)	C(4A)-C(5)-H(5)	119.4
C(624)-C(625)	1.373(5)	C(4)-C(4A)-C(5)	126.3(3)
C(624)-H(624)	0.9300	C(4)-C(4A)-C(8A)	115.5(3)
C(625)-C(626)	1.380(5)	C(5)-C(4A)-C(8A)	118.2(3)

C(625)-H(625)	0.9300	C(3)-C(4)-C(4A)	120.2(3)
C(626)-C(621)	1.383(4)	C(3)-C(4)-C(41)	119.0(3)
C(626)-H(626)	0.9300	C(4A)-C(4)-C(41)	120.8(3)
C(621)-C(622)	1.386(4)	C(4)-C(3)-C(2)	120.5(3)
C(621)-C(62)	1.453(4)	C(4)-C(3)-H(3)	119.7
C(62)-H(62)	0.9300	C(2)-C(3)-H(3)	119.7
C(6)-C(5)	1.359(4)	N(1)-C(2)-C(3)	121.5(3)
C(6)-C(7)	1.408(4)	N(1)-C(2)-C(21)	117.1(3)
C(5)-C(4A)	1.423(4)	C(3)-C(2)-C(21)	121.3(3)
C(5)-H(5)	0.9300	C(26)-C(21)-C(22)	118.1(3)
C(4A)-C(4)	1.412(4)	C(26)-C(21)-C(2)	119.9(3)
C(4A)-C(8A)	1.427(4)	C(22)-C(21)-C(2)	122.0(3)
C(4)-C(3)	1.358(4)	C(23)-C(22)-C(21)	121.1(3)
C(4)-C(41)	1.495(4)	C(23)-C(22)-H(22)	119.4
C(3)-C(2)	1.414(4)	C(21)-C(22)-H(22)	119.4
C(3)-H(3)	0.9300	C(24)-C(23)-C(22)	120.2(3)
C(2)-C(21)	1.481(4)	C(24)-C(23)-H(23)	119.9
C(21)-C(26)	1.387(4)	C(22)-C(23)-H(23)	119.9
C(21)-C(22)	1.389(4)	C(23)-C(24)-C(25)	119.7(3)
C(22)-C(23)	1.378(4)	C(23)-C(24)-H(24)	120.2
C(22)-H(22)	0.9300	C(25)-C(24)-H(24)	120.2
C(23)-C(24)	1.366(4)	F(411)-C(41)-F(413)	106.4(3)
C(23)-H(23)	0.9300	F(411)-C(41)-F(412)	105.6(3)
C(24)-C(25)	1.372(4)	F(413)-C(41)-F(412)	106.2(3)
C(24)-H(24)	0.9300	F(411)-C(41)-C(4)	113.1(3)

C(25)-C(26)	1.383(4)	F(413)-C(41)-C(4)	112.4(3)
C(25)-H(25)	0.9300	F(412)-C(41)-C(4)	112.5(3)
C(26)-H(26)	0.9300	C(24)-C(25)-C(26)	120.7(3)
C(8A)-C(8)	1.410(4)	C(24)-C(25)-H(25)	119.7
C(8)-C(7)	1.363(4)	C(26)-C(25)-H(25)	119.7
C(8)-H(8)	0.9300	C(25)-C(26)-C(21)	120.3(3)
C(7)-H(7)	0.9300	C(25)-C(26)-H(26)	119.9
C(622)-C(623)	1.396(4)	C(21)-C(26)-H(26)	119.9
C(623)-H(623)	0.9300	N(1)-C(8A)-C(8)	117.6(3)
C(622)-O(622)-H(622)	109.5	N(1)-C(8A)-C(4A)	123.6(3)
C(62)-N(61)-C(6)	119.9(3)	C(8)-C(8A)-C(4A)	118.8(3)
C(2)-N(1)-C(8A)	118.6(2)	C(7)-C(8)-C(8A)	121.3(3)
C(623)-C(624)-C(625)	121.2(4)	C(7)-C(8)-H(8)	119.4
C(623)-C(624)-H(624)	119.4	C(8A)-C(8)-H(8)	119.4
C(625)-C(624)-H(624)	119.4	C(8)-C(7)-C(6)	120.2(3)
C(624)-C(625)-C(626)	118.5(4)	C(8)-C(7)-H(7)	119.9
C(624)-C(625)-H(625)	120.8	C(6)-C(7)-H(7)	119.9
C(626)-C(625)-H(625)	120.8	O(622)-C(622)-C(621)	122.2(3)
C(625)-C(626)-C(621)	121.9(4)	O(622)-C(622)-C(623)	118.1(3)
C(625)-C(626)-H(626)	119.0	C(621)-C(622)-C(623)	119.7(3)
C(621)-C(626)-H(626)	119.0	C(624)-C(623)-C(622)	120.2(4)
C(626)-C(621)-C(622)	118.5(3)	C(624)-C(623)-H(623)	119.9
C(626)-C(621)-C(62)	119.8(3)	C(622)-C(623)-H(623)	119.9

2. Photophysical analyses and photostability assays

For spectroscopic analysis, UV–vis absorption spectra were recorded using a Shimadzu UV2600 spectrophotometer (2.0 nm data range), using DMSO, MeOH, or chloroform as solvent. The steady-state emission fluorescence spectra in DMSO, MeOH or chloroform solutions were measured with a Cary50 Eclipse Fluorescence Spectrophotometer (excitation/emission; slit 2.5 mm). The values of the quantum fluorescence yield (Φ_f) were determined in solution, using the compound 9,10-diphenylanthracene (DPA) in chloroform ($\Phi_f = 0.65$, $\lambda_{exc} = 366$ nm) as a comparison emission standard, according Equation (1):

$$\phi_F = \phi_{Fstd} \frac{I}{I_{\mathrm{std}}} \frac{(1-10^{-A})_{std}}{(1-10^{-A})} \frac{\eta}{\eta_{\mathrm{std}}} \quad \textbf{(1)}$$

where Φ_F, *I*, A and η are the fluorescence quantum yield, the integrated area of the emission band, the absorbance in the excitation wavelength (λ_{exc}) and refractive index of the solvent, respectively. The subscript "std" refers to the 9,10-diphenylanthracene; DPA.

Photostability assays were performed using white-light LED array system irradiation (visible range) at 25 mW/cm^2 and total light dosage 90 J/cm^2 at 60 min, according to the current literature [3-5]. All experiments were performed in duplicate and independently.

DMSO solutions of compounds at 1.0 µM were freshly prepared and kept in the dark at room temperature. The photo-irradiation experiments were performed in magnetically stirred cuvette solutions (with 2.0 mL of sample), over a period of 30 min with white-light LED array system at fluence of 25 mW/cm^2 and total light dosages of

90 J/cm^2 (60 min). The absorbance was determined before irradiation and at 0, 5, 10, 15, 20, 30, 40, 50, and 60 min after irradiation. The results were expressed as follows using Equation (2):

$$Photostability(\%) = \frac{Abs\ at\ a\ given\ time\ of\ irradiation}{Abs\ before\ irradiation} x100\% \quad \textbf{(2)}$$

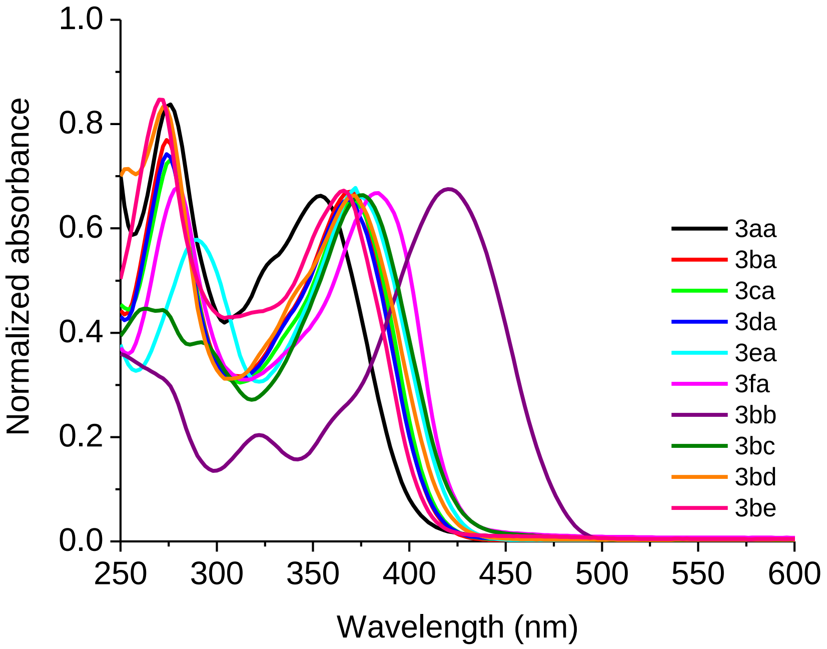

Figure S2: UV–vis absorption spectra of compounds **3aa–fa** and **3bb–be** in $CHCl_3$ solution ([ ] = 1.50 × 10^{-5} M).

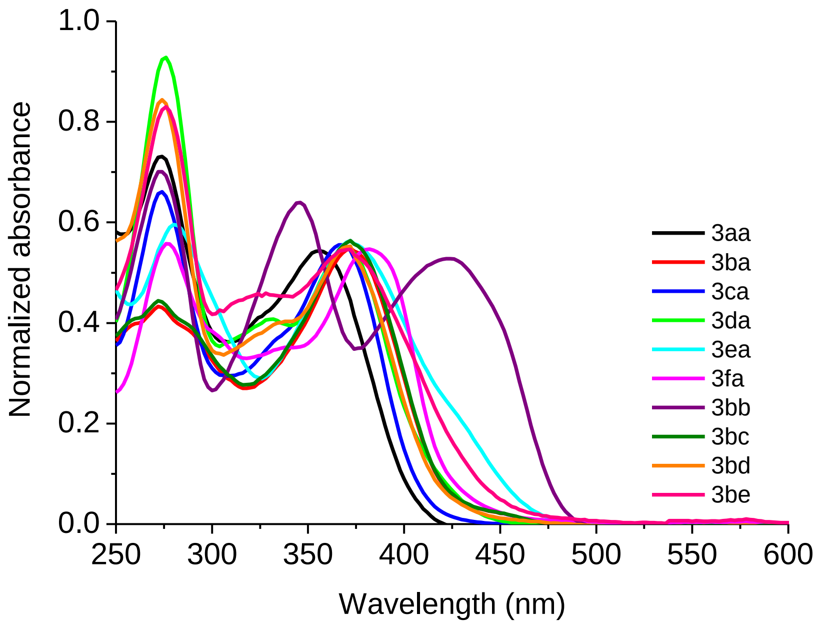

Figure S3: UV–vis absorption spectra of compounds **3aa–fa** and **3bb–be** in MeOH solution ([ ] = 1.50 × 10^{-5} M).

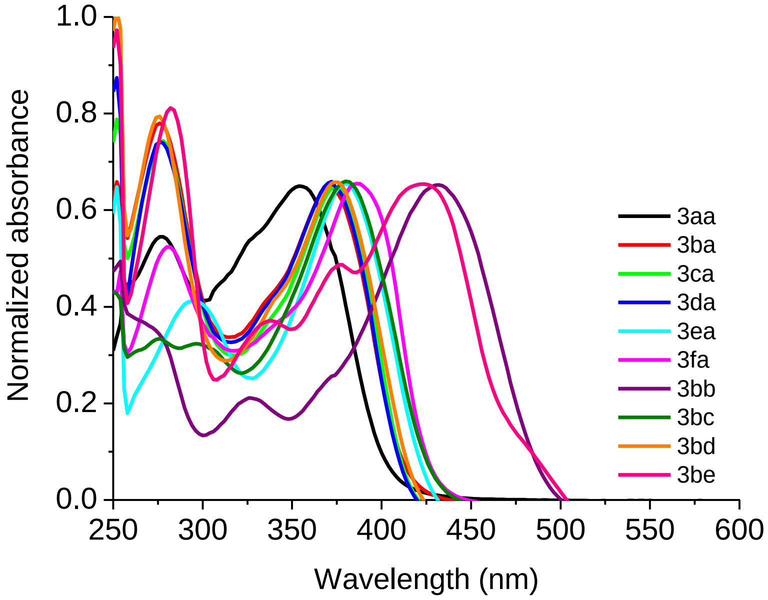

Figure S4: UV–vis absorption spectra of compounds **3aa–fa** and **3bb–be** in DMSO solution ([ ] = 1.50 × 10^{-5} M).

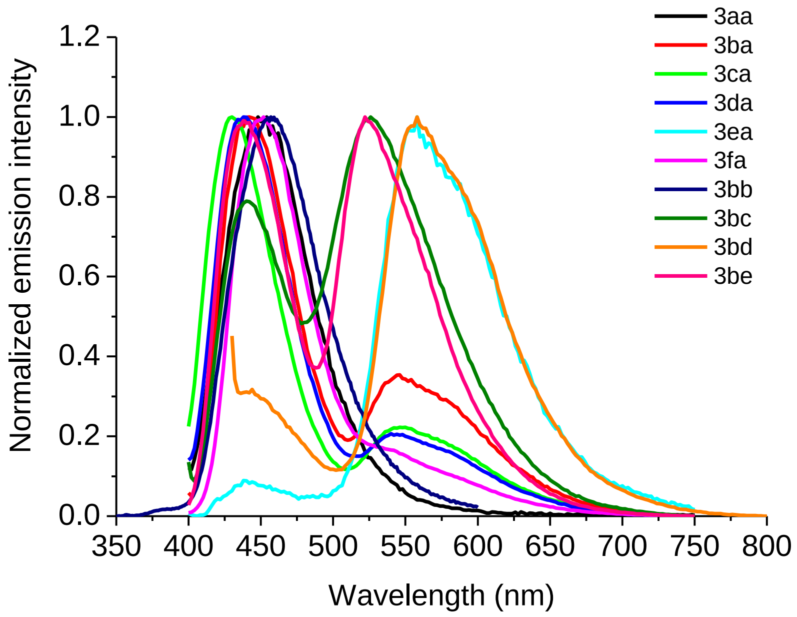

Figure S5: Normalized steady-state fluorescence emission spectra of all compound in $CHCl_3$ solution ([ ] = 1.50 × 10^{-5} M).

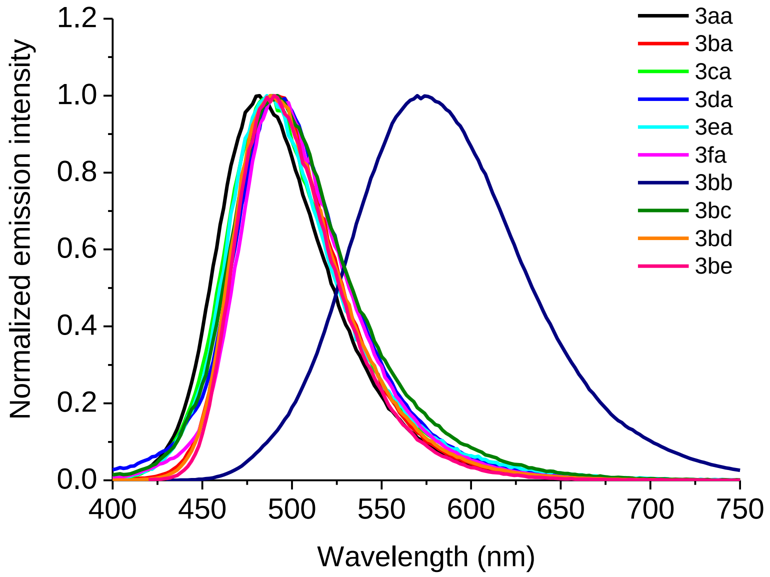

Figure S6: Normalized steady-state fluorescence emission spectra of all compound in DMSO solution ([ ] = 1.50 × 10^{-5} M).

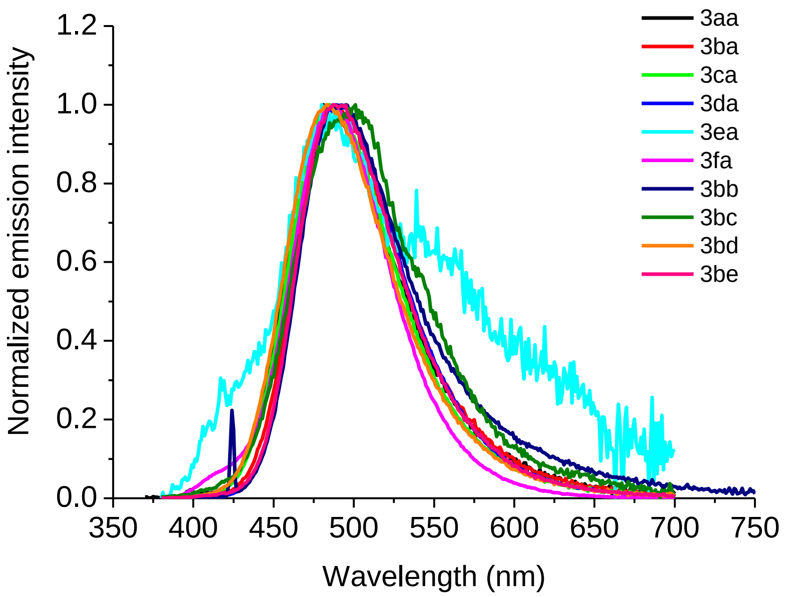

Figure S7: Normalized steady-state fluorescence emission spectra of all compound in MeOH solution ([ ] = 1.50 × 10^{-5} M).

3. Singlet oxygen quantum yield (Φ_Δ) measurements

In a typical experiment of 1,3-diphenylisobenzofuran (DPBF) singlet oxygen quencher photo-oxidation [6], solutions containing DPBF (50 µM) with or without Schiff bases derivatives 0.50 µM were prepared in DMSO in a quartz cuvette. In order to measure $^1O_2$ generation, UV–vis spectra of the solutions (samples and standard) were recorded for different exposure times by using a 660 nm red diode laser positioned 2.0 cm from the sample (TheraLase DMC, São Carlos, SP, Brazil) with an average power of 100 mW, during 10 min (irradiation intervals every 30 s). The singlet oxygen production quantum yield (Φ_Δ) was calculated by using Equation (3):

$$\Phi_\Delta = \Phi_\Delta^{std} \frac{k}{k^{std}} \frac{I^{std}}{I} \qquad \textbf{(3)}$$

in which, $I^{std} / I = (1 - 10^{Astd}) / (1 - 10^{A})$, Φ_Δ^{std} is the singlet oxygen quantum yield of standard sample (in our case, methylene blue (MB) in ethanol solution, Φ_Δ^{std} = 0.52)

[7], k and k^{std} are the photo-oxidation kinetic constants for the Schiff bases and MB (standard), respectively, and A^{std} and A are the absorbances of MB and studied compounds, respectively.

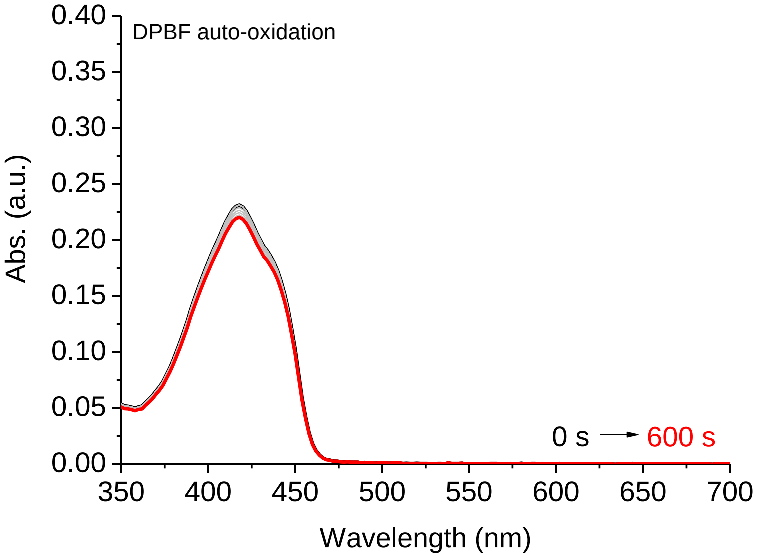

Figure S8: Photo-oxidation of DPBF by red-light irradiation with a diode laser (λ = 660 nm) in the absence of derivatives.

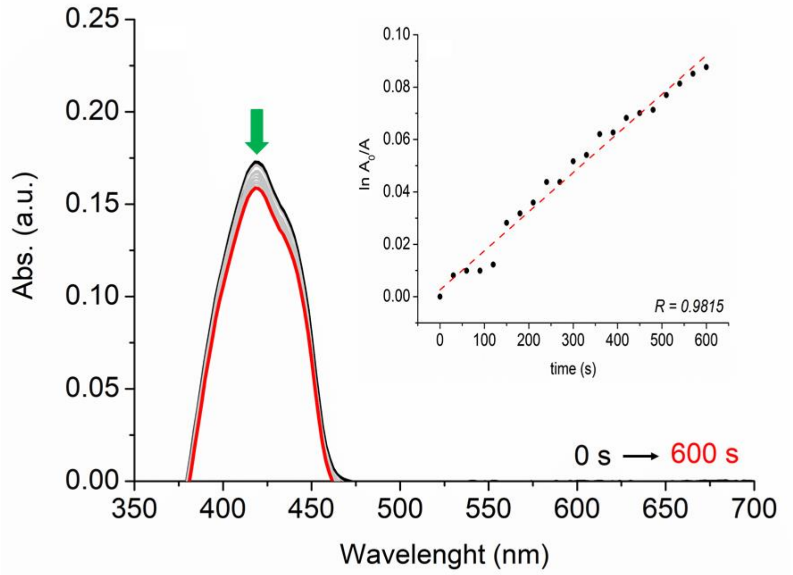

Figure S9: Photo-oxidation of DPBF by red-light irradiation with a diode laser (λ = 660 nm) in the presence of compound **3aa**. The *inset* shows the first-order kinetic profile.

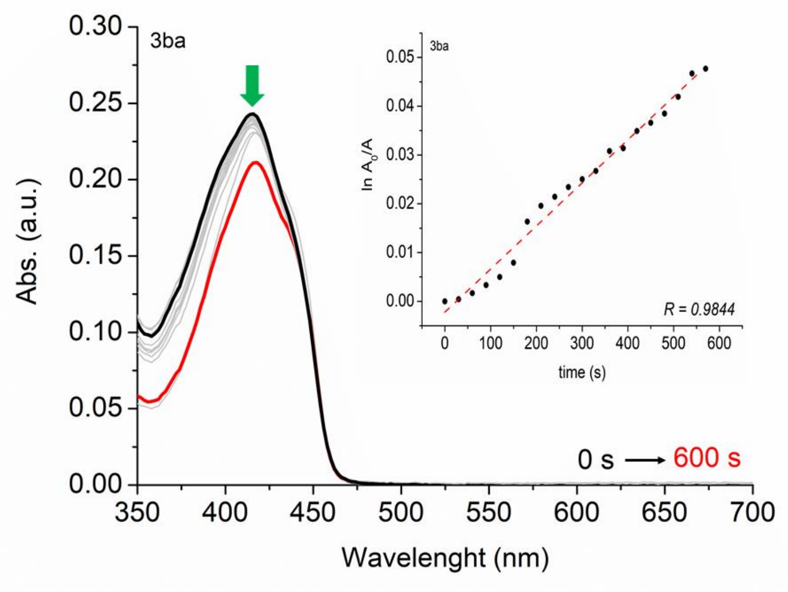

Figure S10. Photo-oxidation of DPBF by red-light irradiation with a diode laser (λ = 660 nm) in the presence of compound **3ba**. The *inset* shows the first-order kinetic profile.

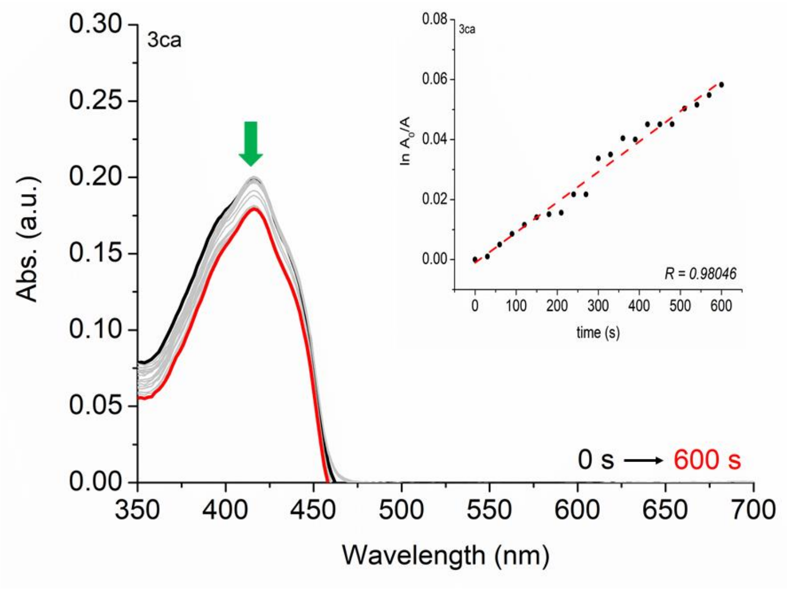

Figure S11. Photo-oxidation of DPBF by red-light irradiation with a diode laser (λ = 660 nm) in the presence of compound **3ca**. The *inset* shows the first-order kinetic profile.

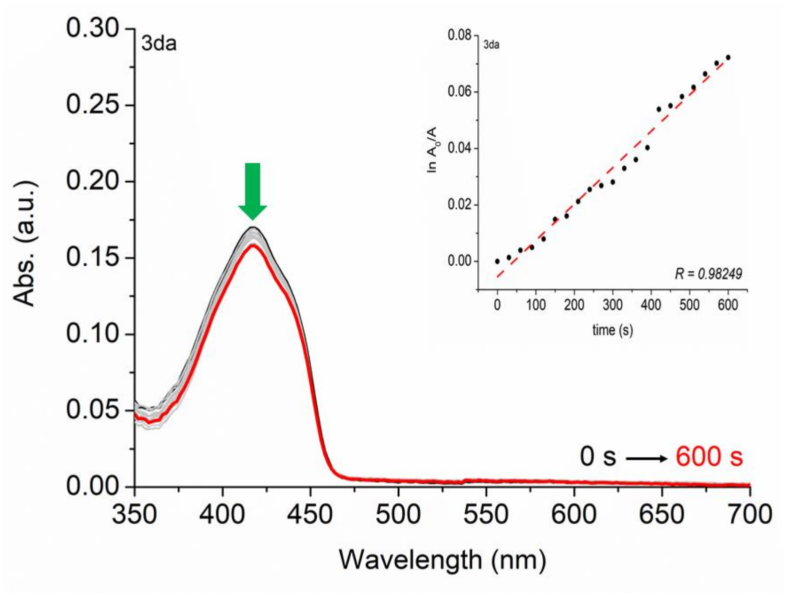

Figure S12. Photo-oxidation of DPBF by red-light irradiation with a diode laser (λ = 660 nm) in the presence of compound **3da**. The *inset* shows the first-order kinetic profile.

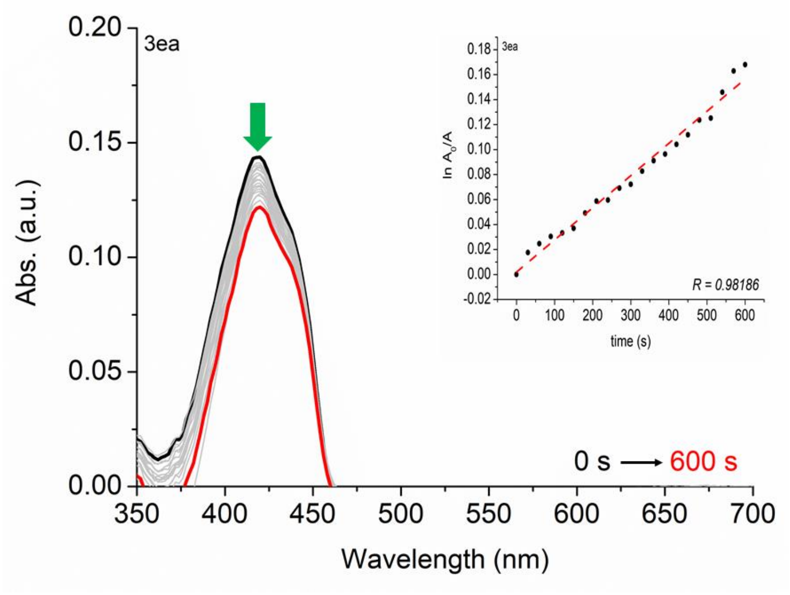

Figure S13. Photo-oxidation of DPBF by red-light irradiation with diode laser (λ = 660 nm) in the presence of compound **3ea**. The *inset* shows the first-order kinetic profile.

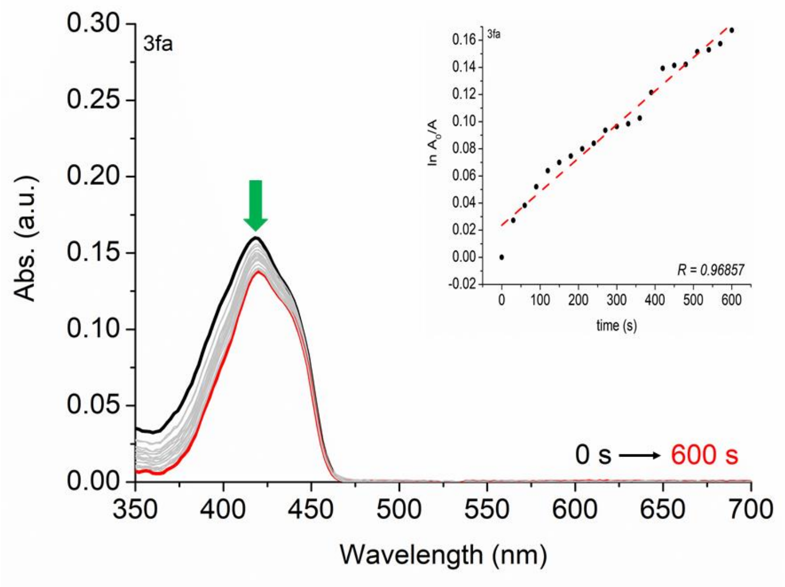

Figure S14. Photo-oxidation of DPBF by red-light irradiation with a diode laser (λ = 660 nm) in the presence of compound **3fa**. The *inset* shows the first-order kinetic profile.

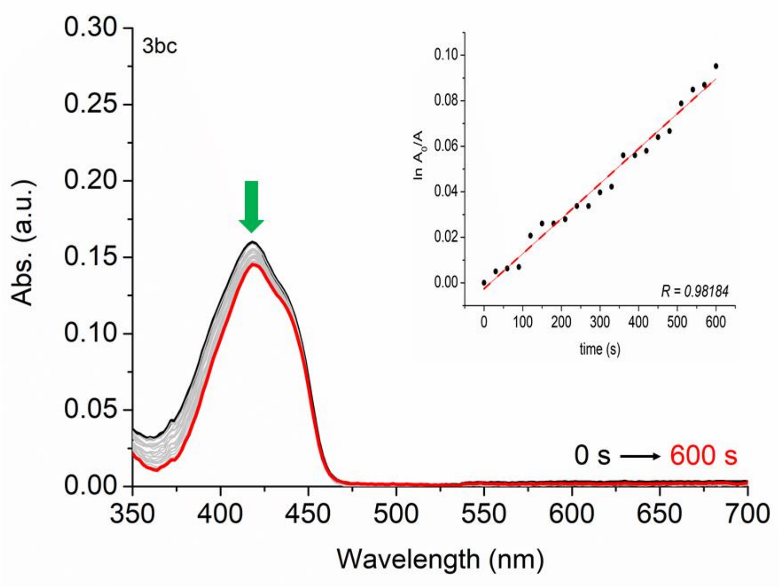

Figure S15. Photo-oxidation of DPBF by red-light irradiation with a diode laser (λ = 660 nm) in the presence of compound **3bc**. The *inset* shows the first-order kinetic profile.

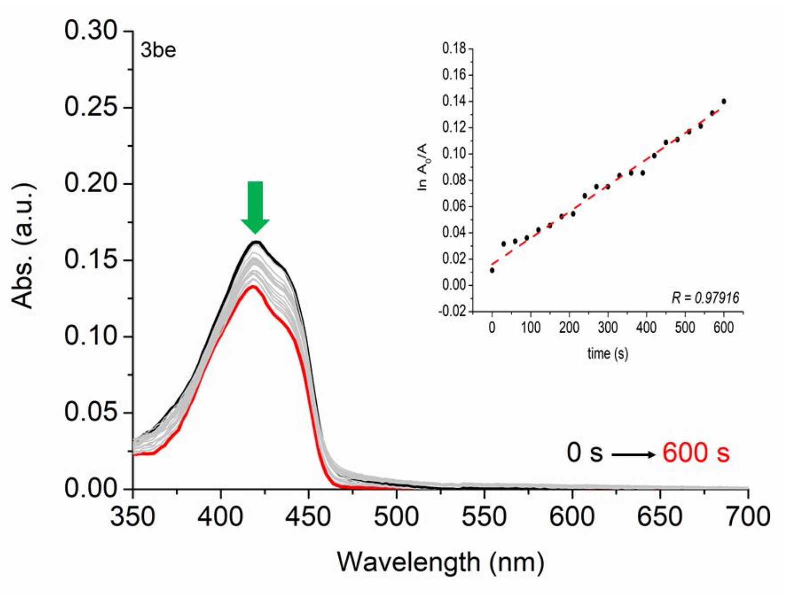

Figure S16. Photo-oxidation of DPBF by red-light irradiation with a diode laser (λ = 660 nm) in the presence of compound **3bd**. The *inset* shows the first-order kinetic profile.

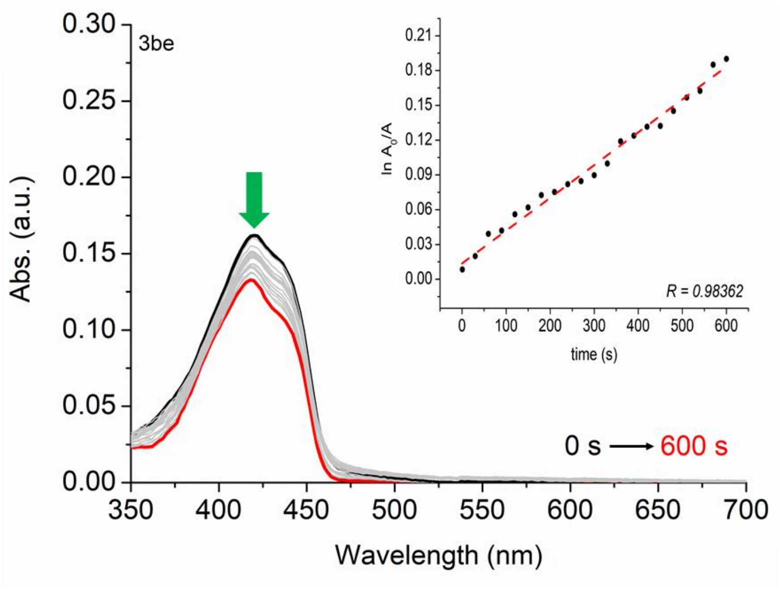

Figure S17. Photo-oxidation of DPBF by red-light irradiation with a diode laser (λ = 660 nm) in the presence of compound **3be**. The *inset* shows the first-order kinetic profile.

4. ^{1}H, ^{13}C and ^{19}F NMR Spectra

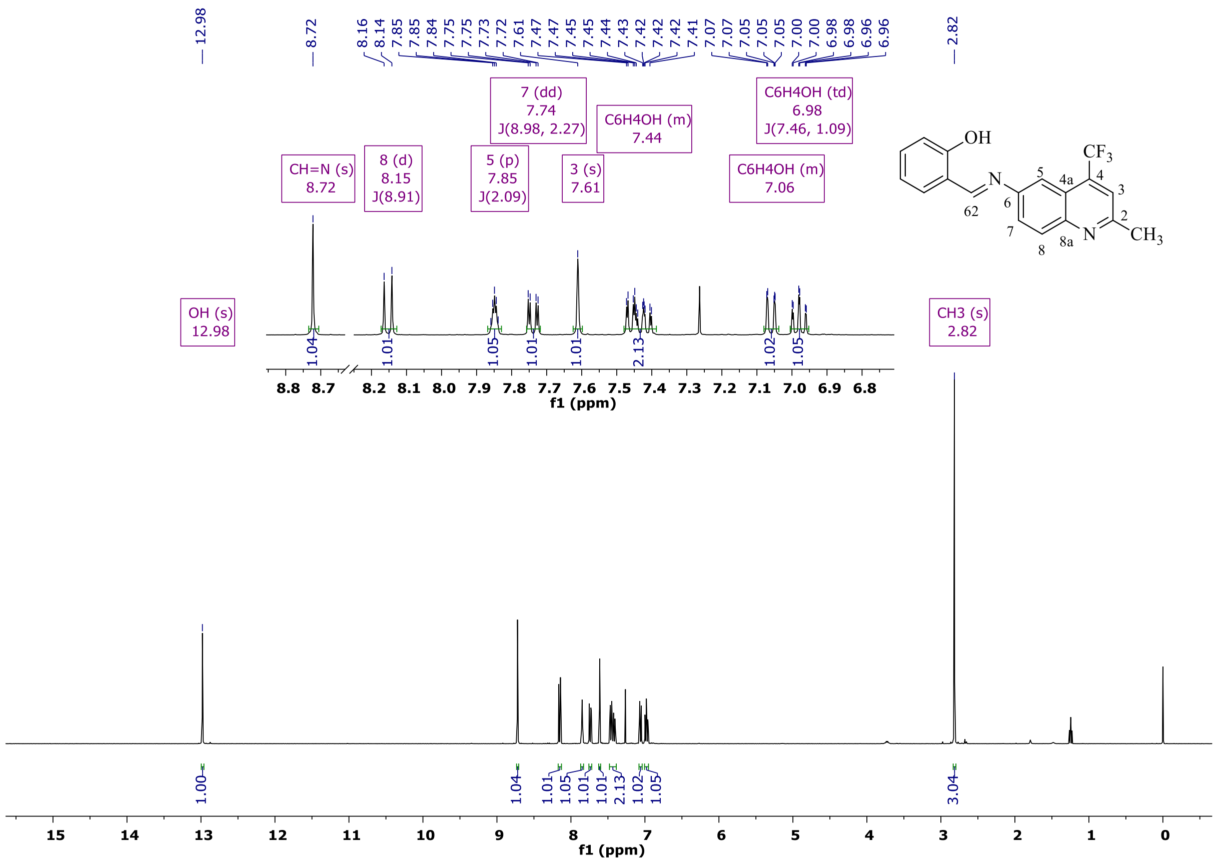

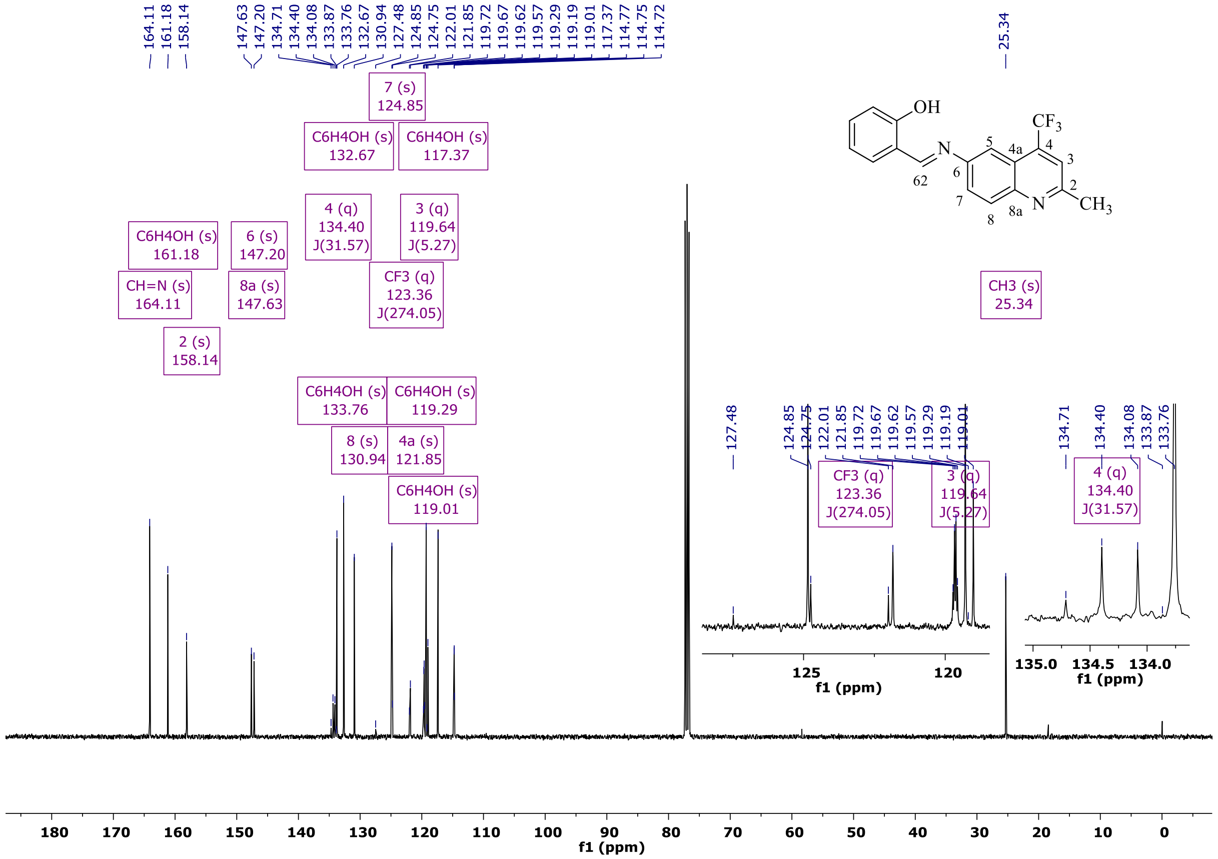

Figure S18: (a) ^{1}H (400 MHz) and (b) ^{13}C (100 MHz) NMR spectra of **3aa** in $CDCl_3$.

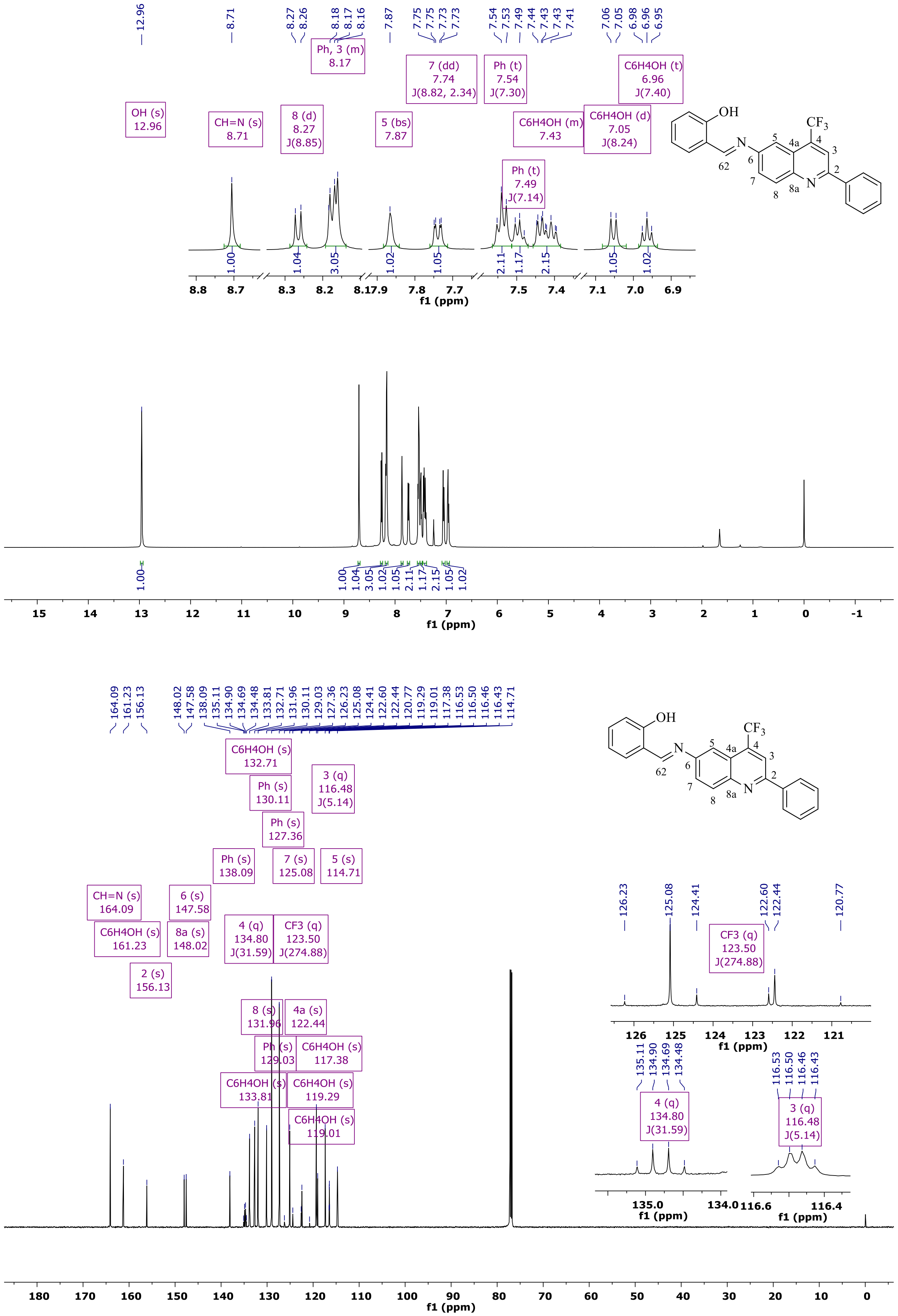

Figure S19: (a) ^{1}H (600 MHz) and (b) ^{13}C (151 MHz) NMR spectra of **3ba** in $CDCl_3$.

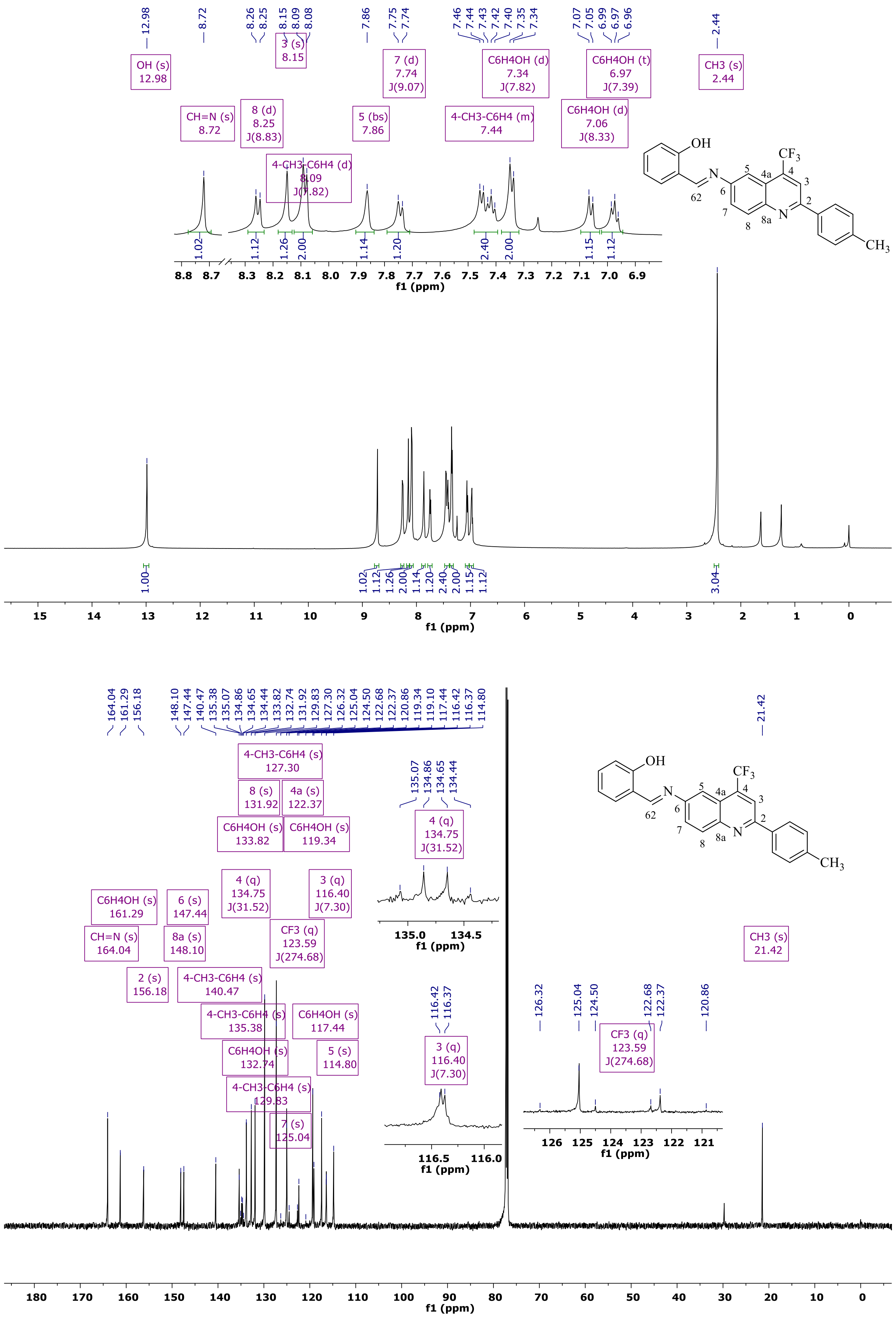

Figure S20: (a) ^{1}H (600 MHz) and (b) ^{13}C (151 MHz) NMR spectra of **3ca** in $CDCl_3$.

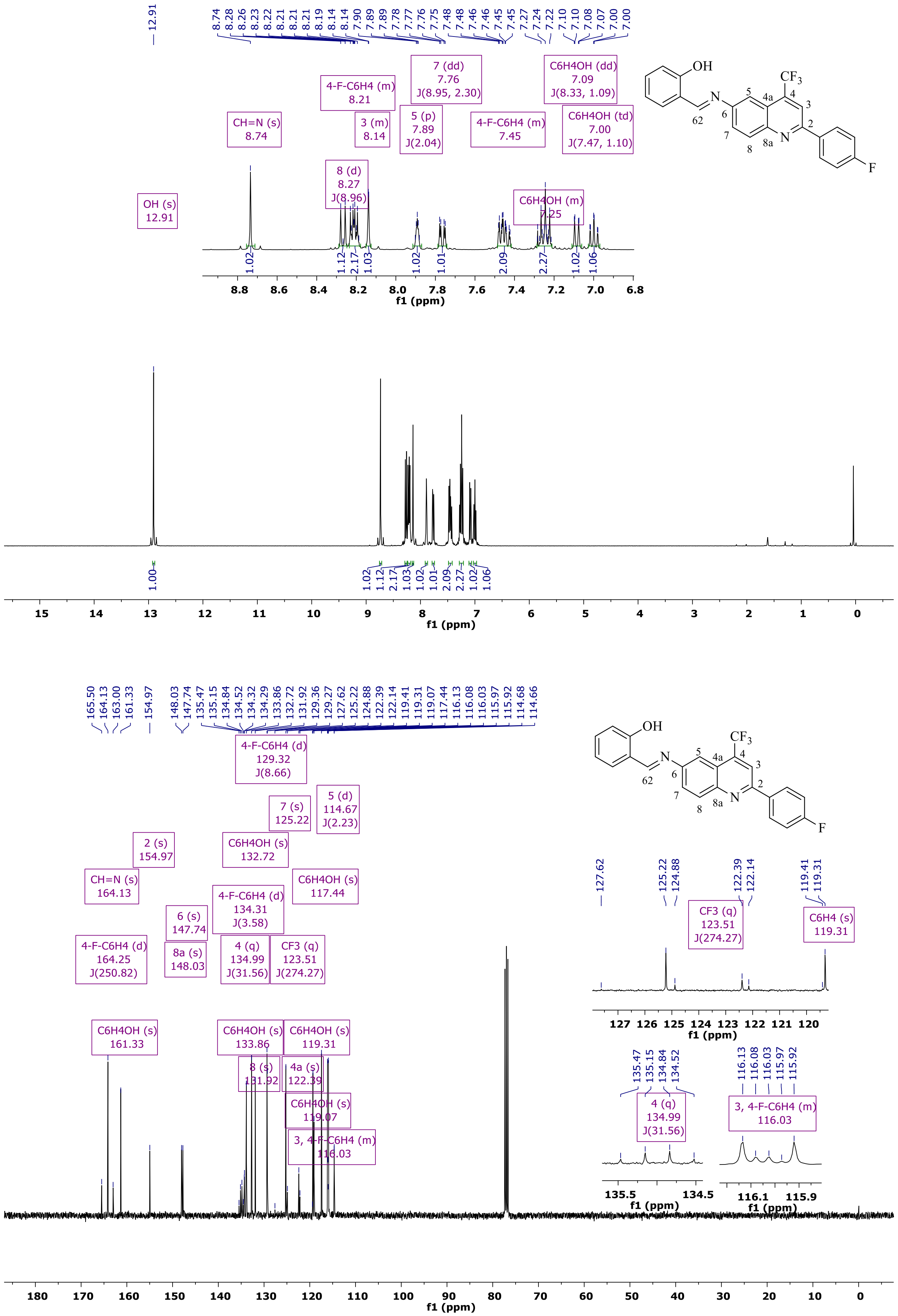

Figure S21: (a) ^{1}H (400 MHz) and (b) ^{13}C (100 MHz) NMR spectra of **3da** in $CDCl_3$.

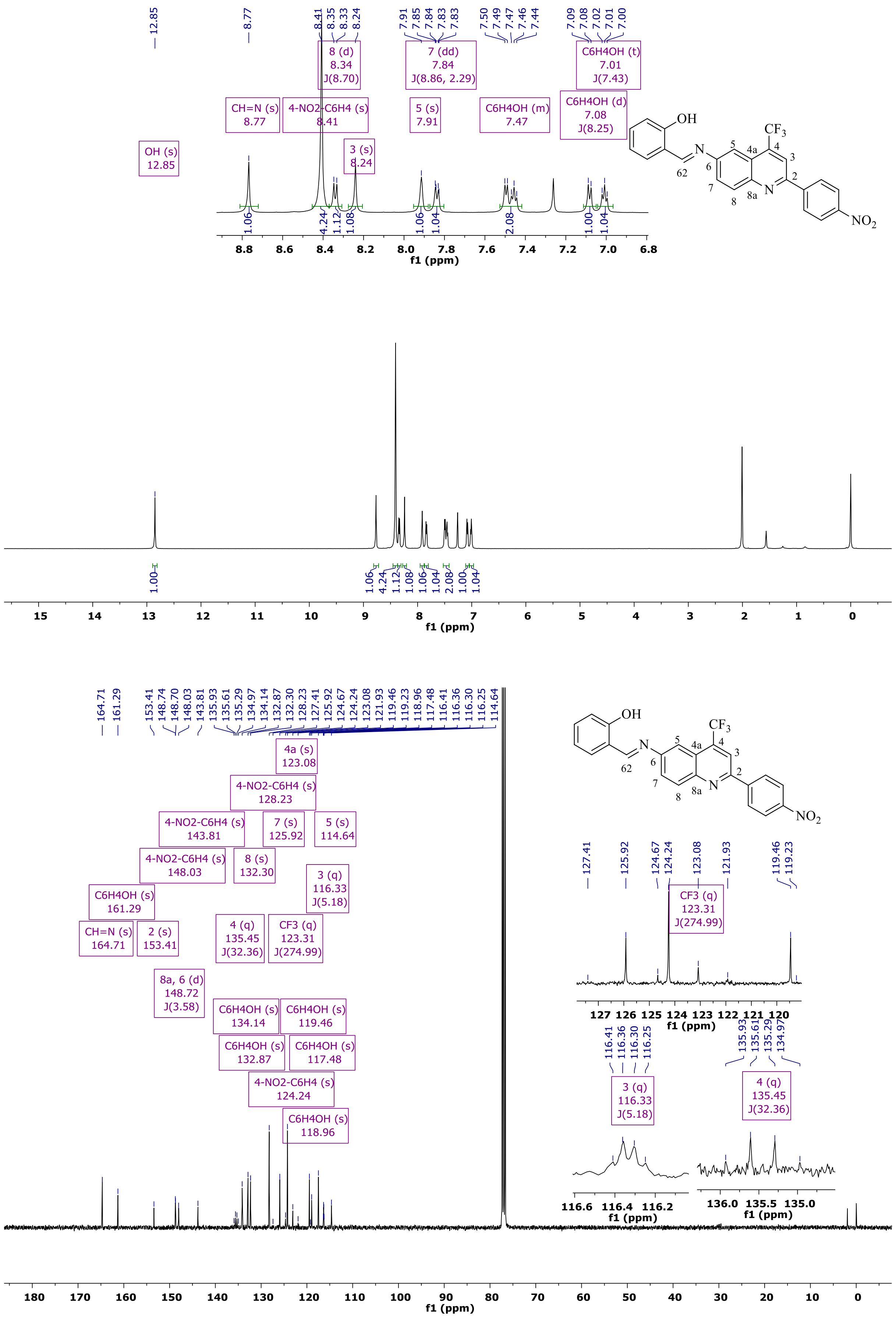

Figure S22: (a) ^{1}H (600 MHz) and (b) ^{13}C (100 MHz) NMR spectra of **3ea** in $CDCl_3$.

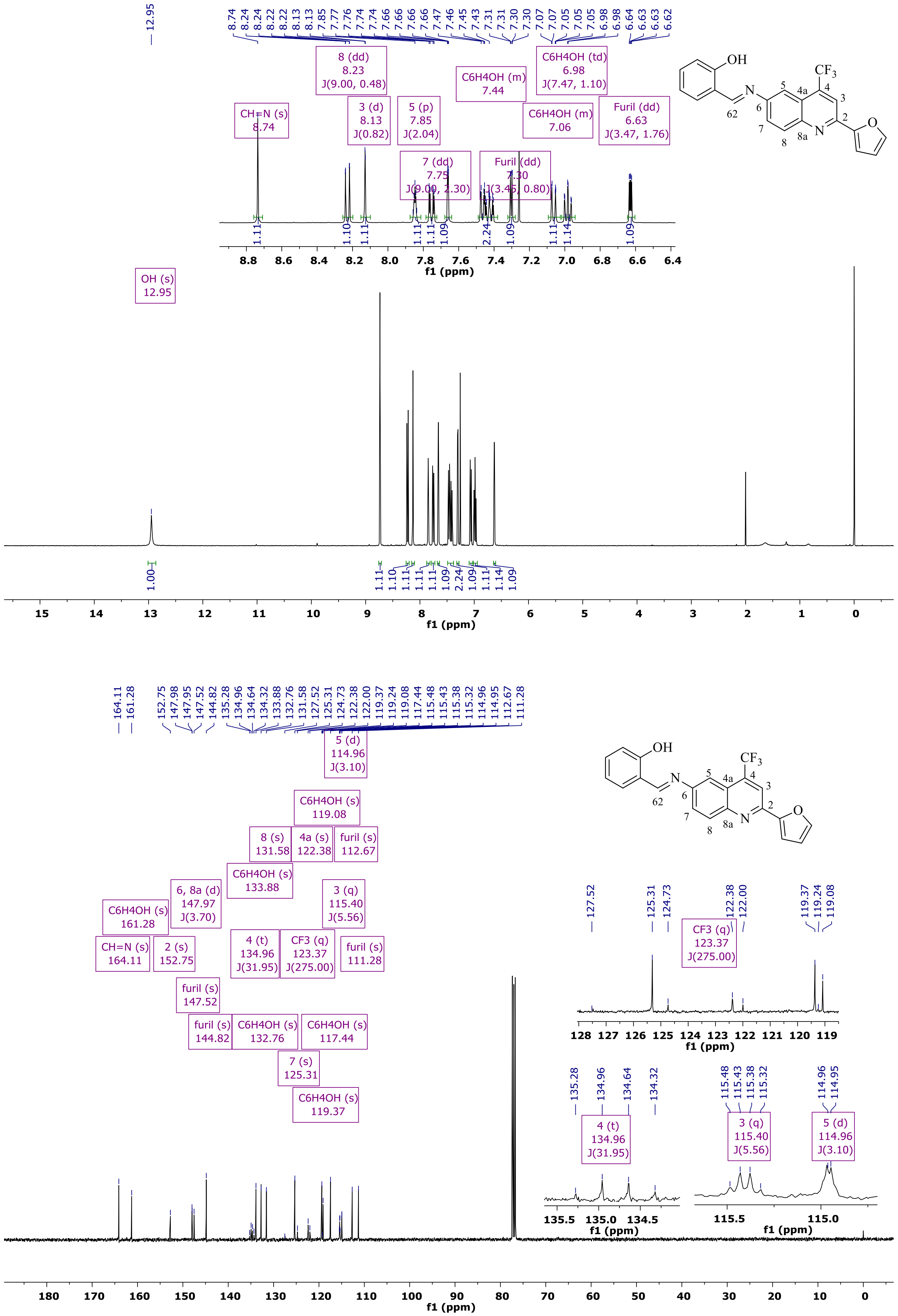

Figure S23: (a) ^{1}H (400 MHz) and (b) ^{13}C (100 MHz) NMR spectra of **3fa** in $CDCl_3$.

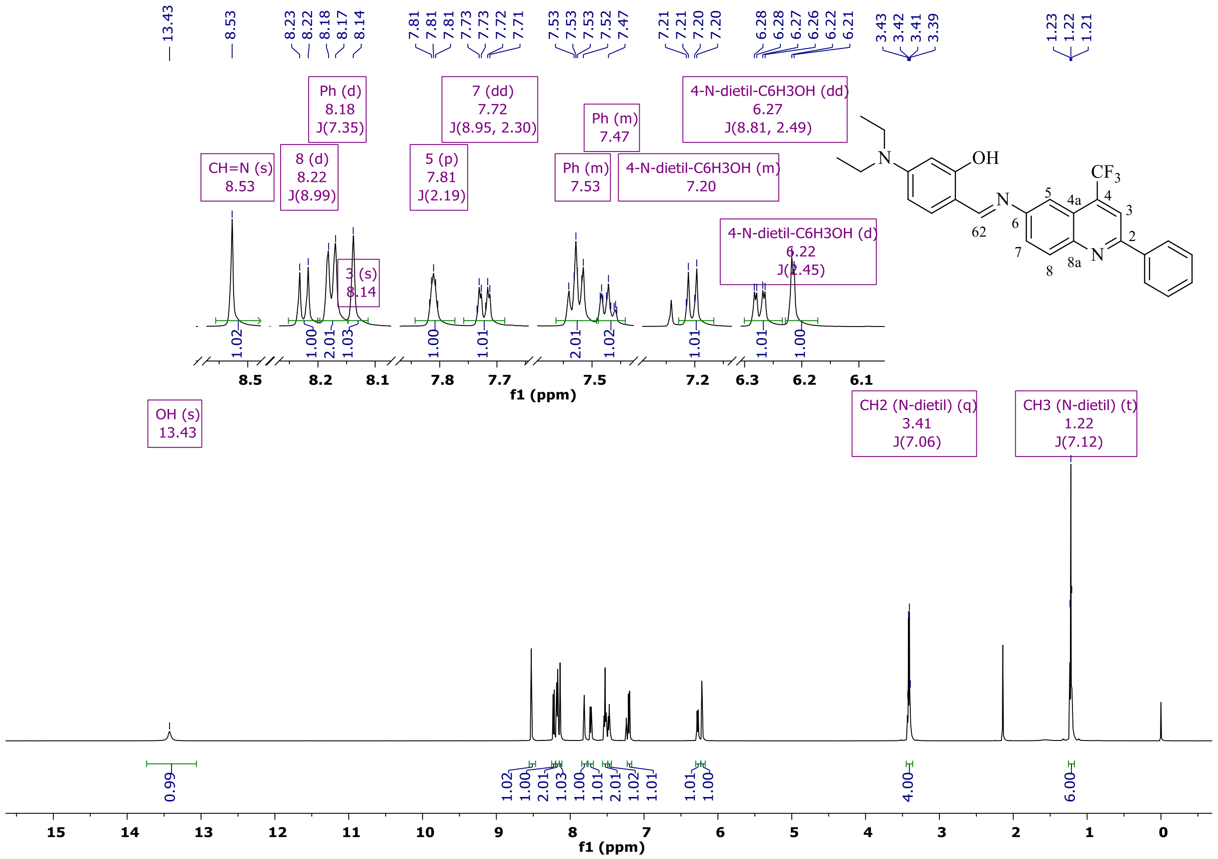

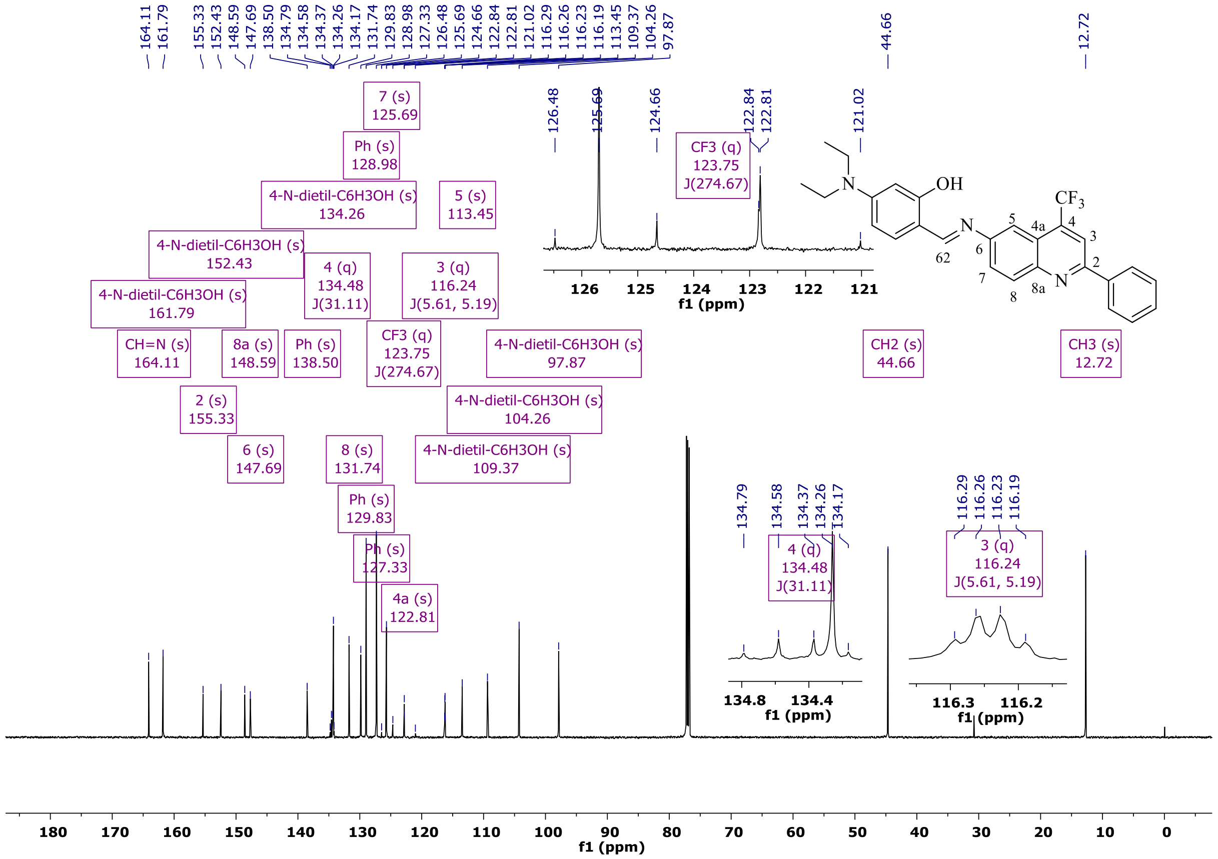

Figure S24: (a) ^{1}H (600 MHz) and (b) ^{13}C (151 MHz) NMR spectra of **3bb** in $CDCl_3$.

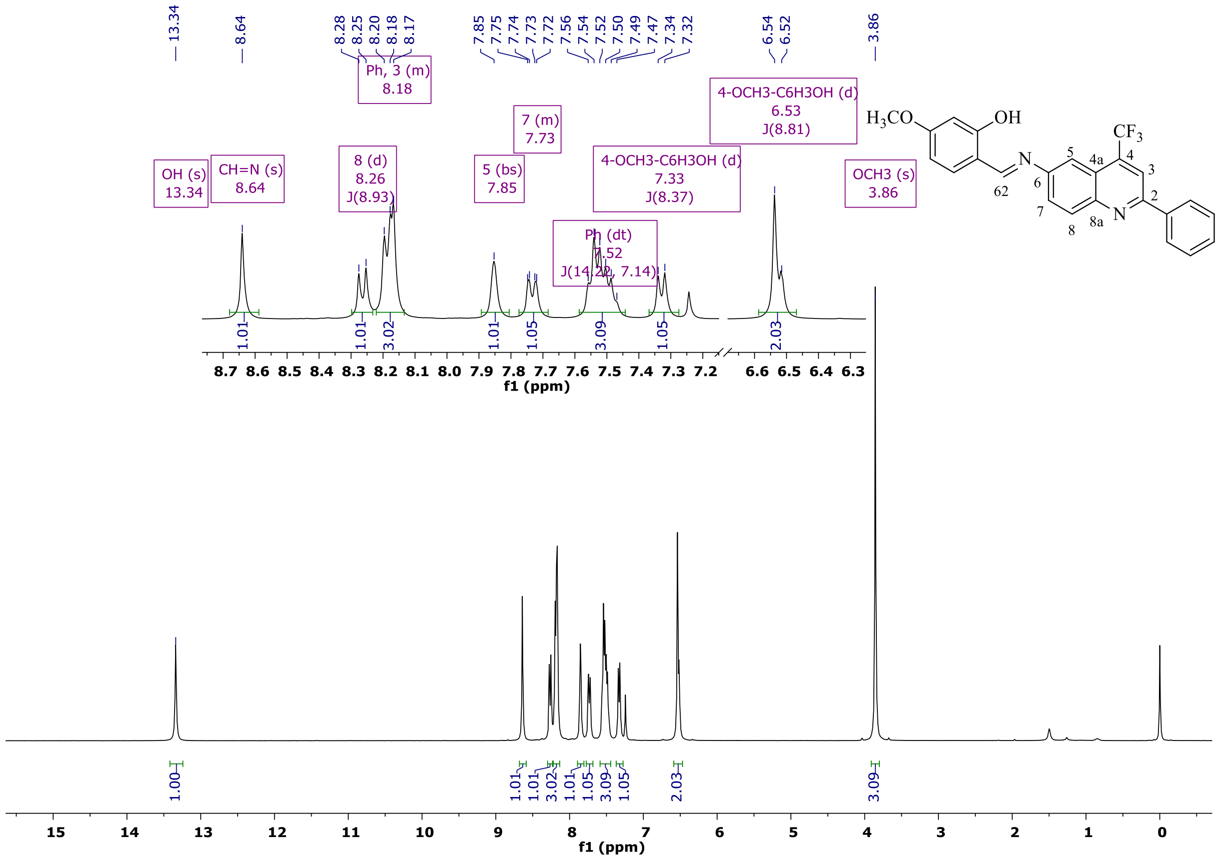

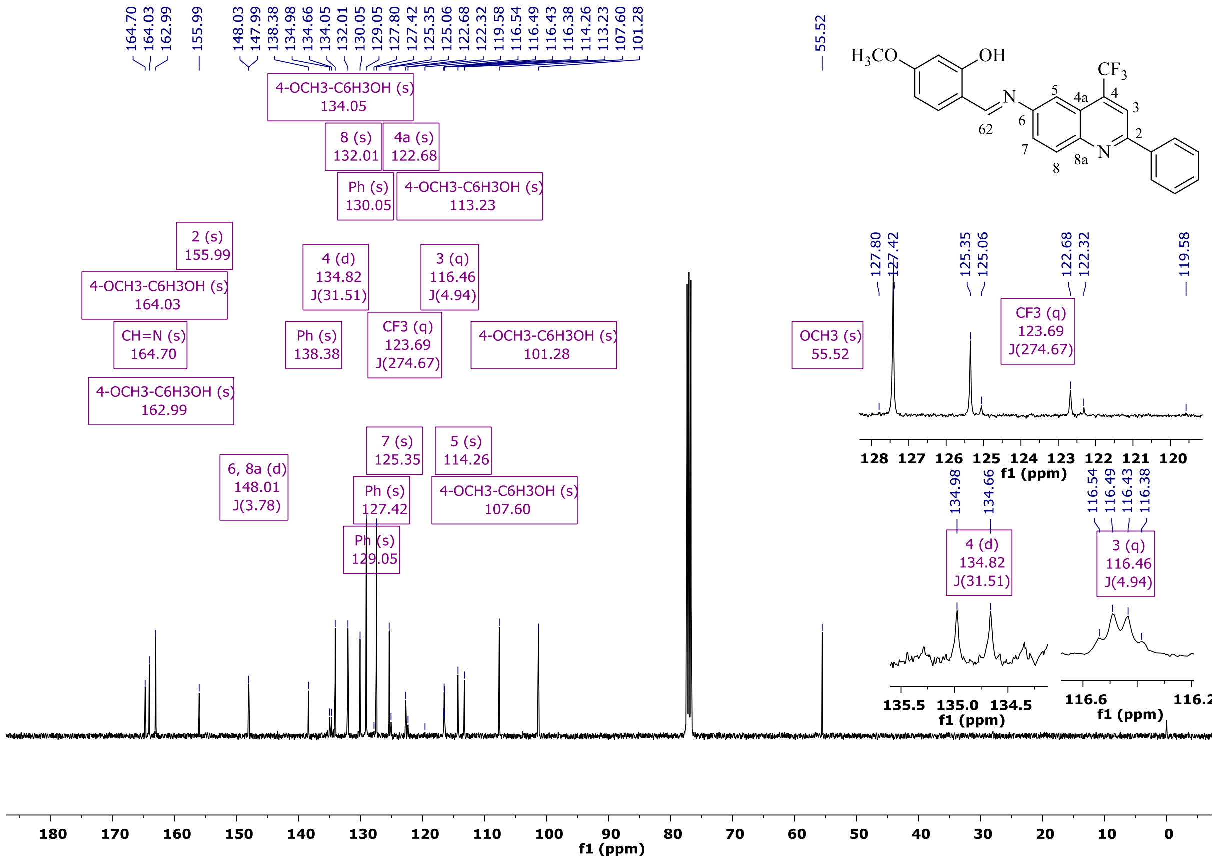

Figure S25: (a) ^{1}H (400 MHz) and (b) ^{13}C (100 MHz) NMR spectra of **3bc** in $CDCl_3$.

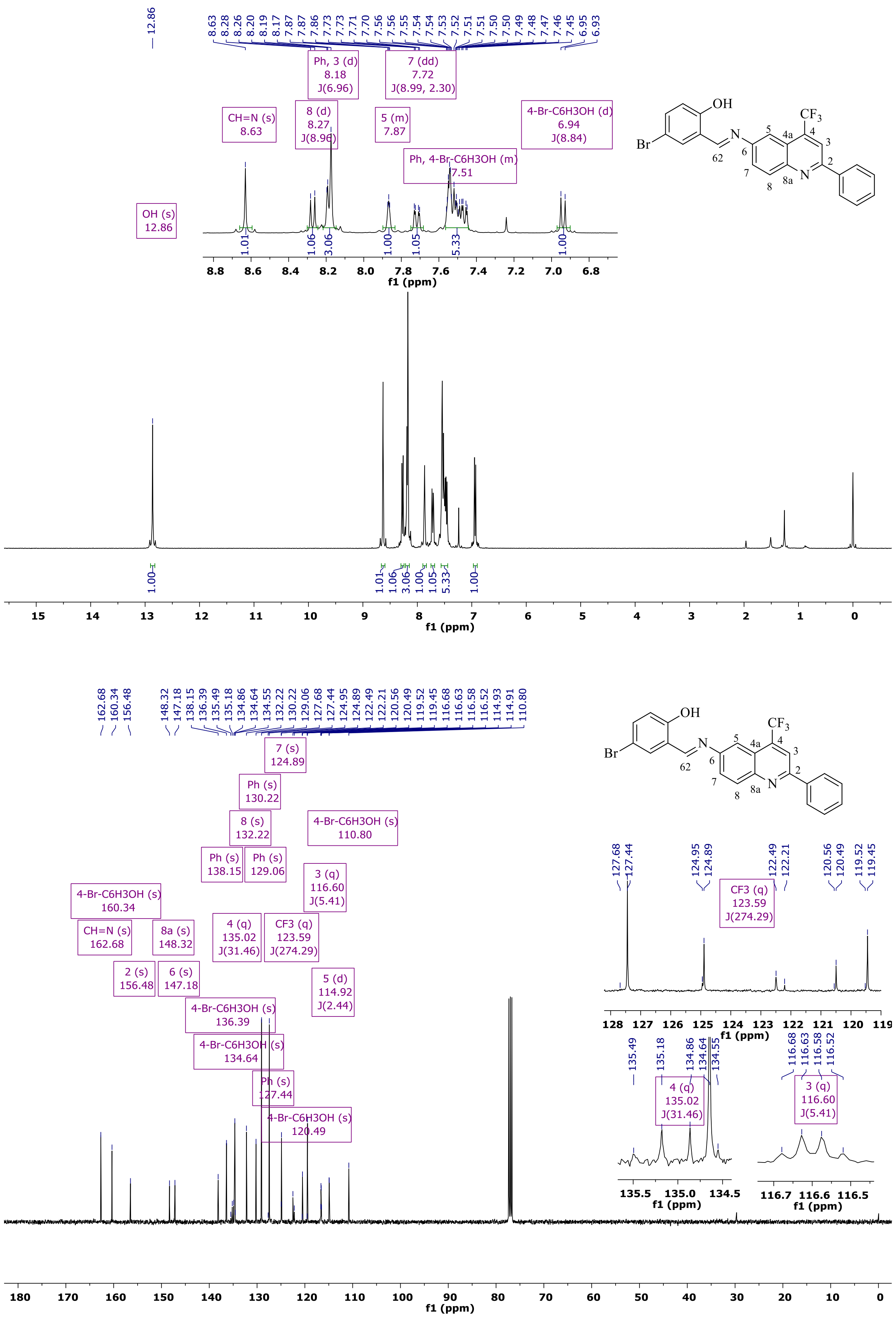

Figure S26: (a) ^{1}H (400 MHz) and (b) ^{13}C (100 MHz) NMR spectra of 3**bd** in $CDCl_3$.

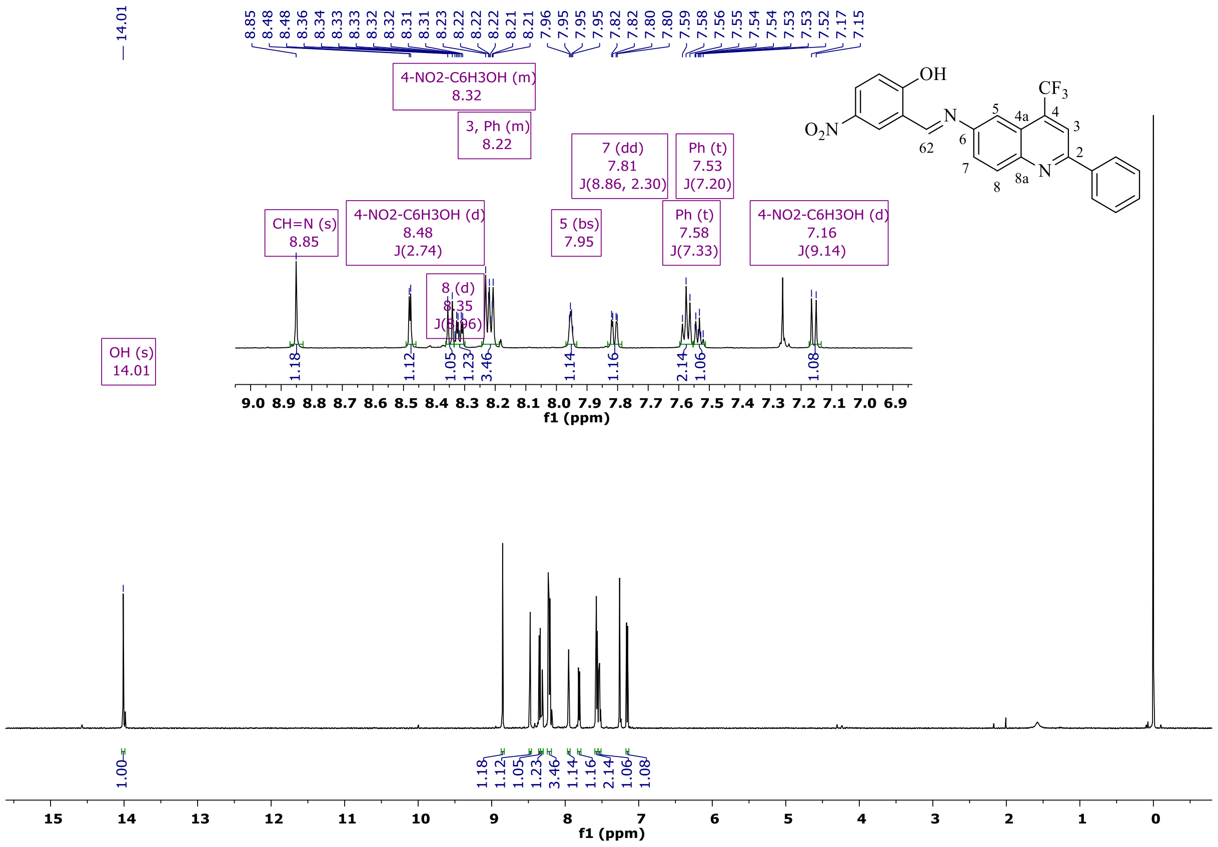

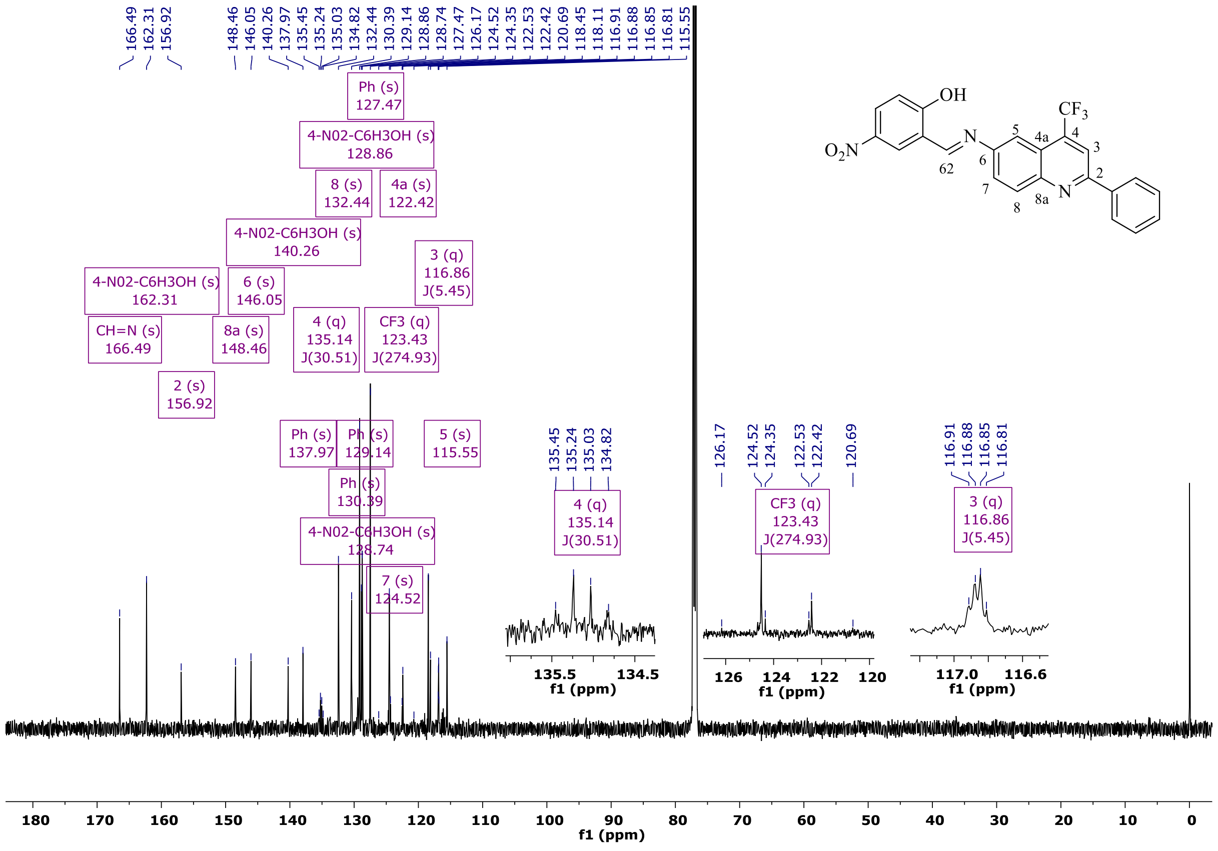

Figure S27: (a) ^{1}H (600 MHz) and (b) ^{13}C (151 MHz) NMR spectra of **3be** in $CDCl_3$.

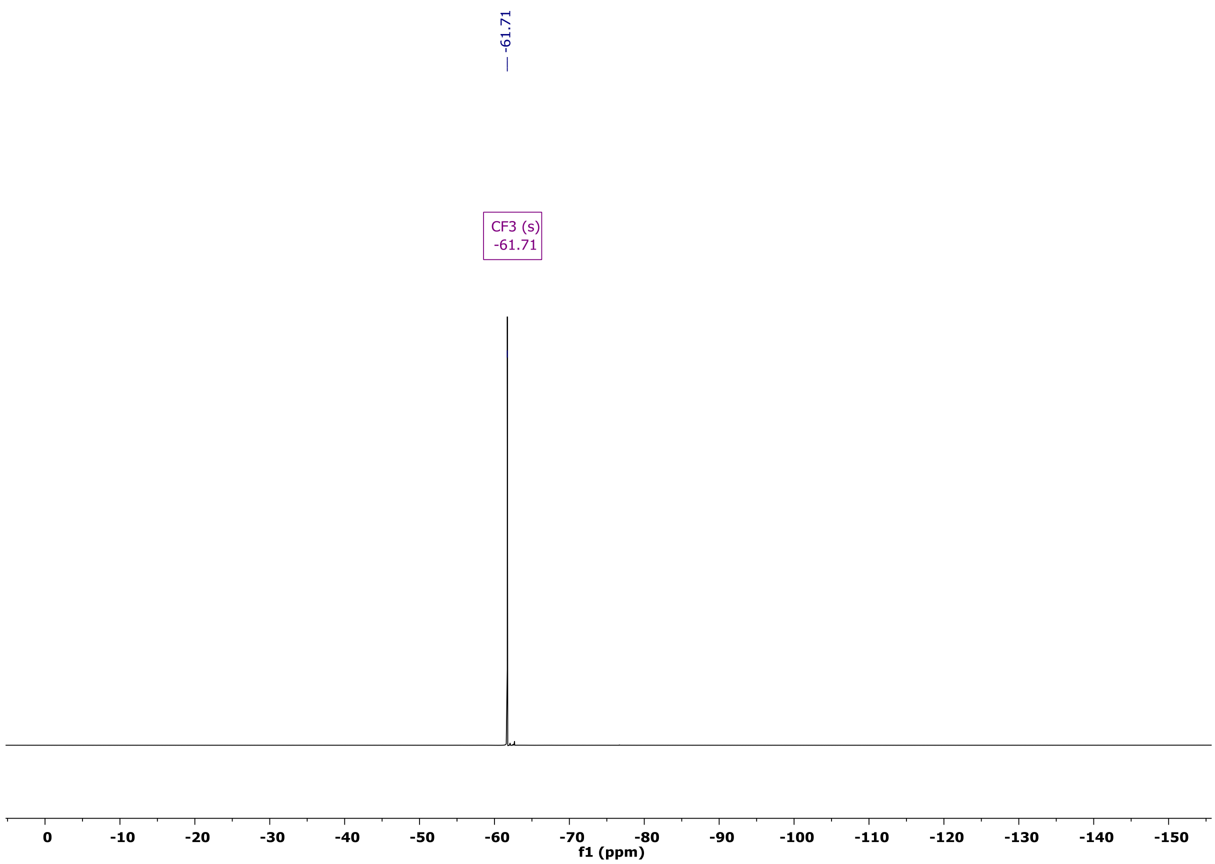

Figure S28: ^{19}F (565 MHz) NMR spectrum of **3aa** in $CDCl_3$.

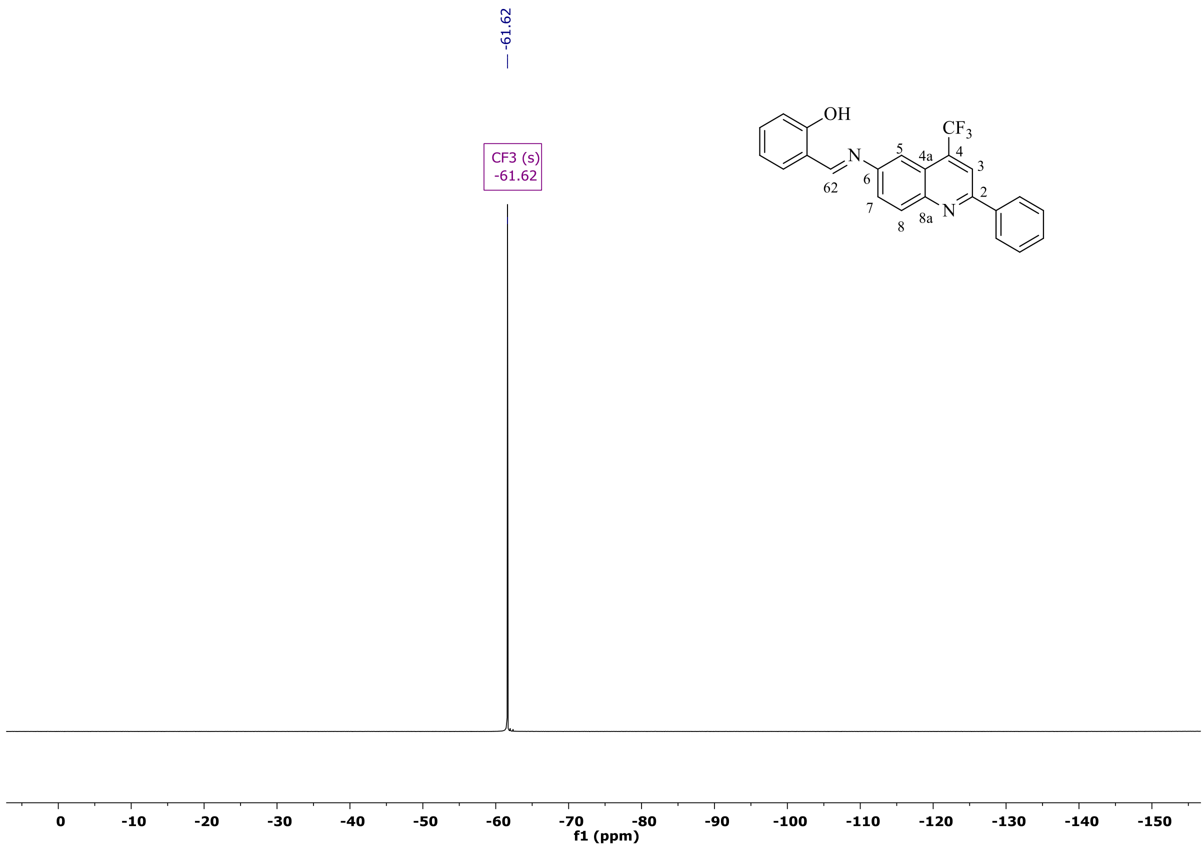

Figure S29: ^{19}F (565 MHz) NMR spectrum of **3ba** in $CDCl_3$.

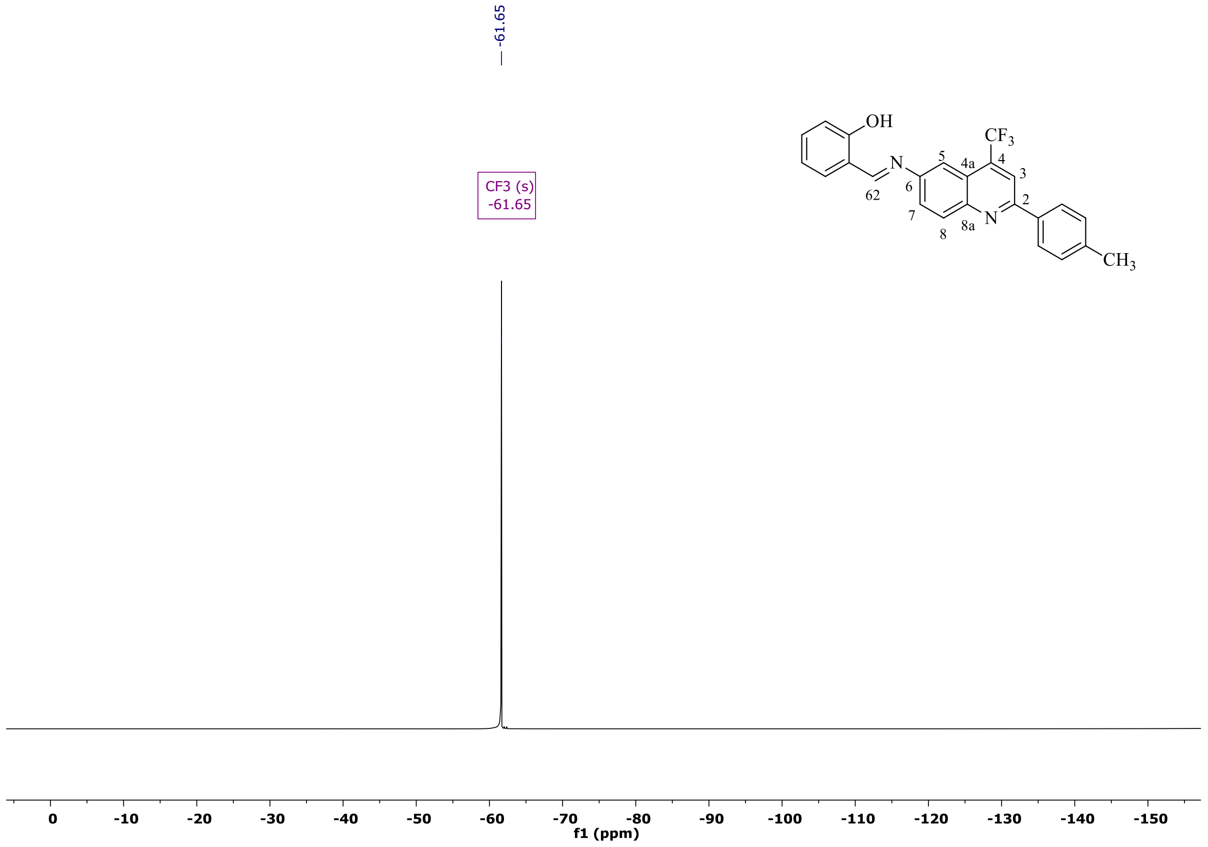

Figure S30: ^{19}F (565 MHz) NMR spectrum of **3ca** in $CDCl_3$.

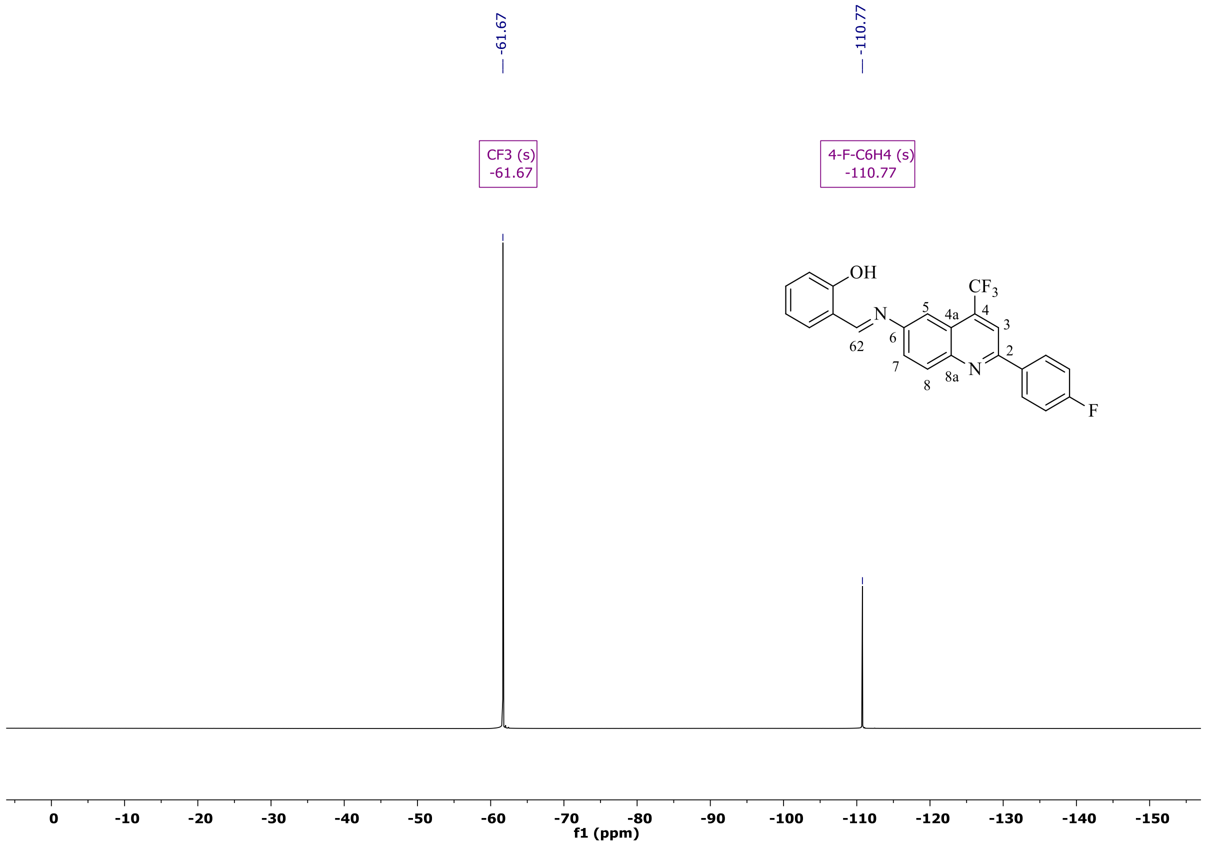

Figure S31: ^{19}F (565 MHz) NMR spectrum of **3da** in $CDCl_3$.

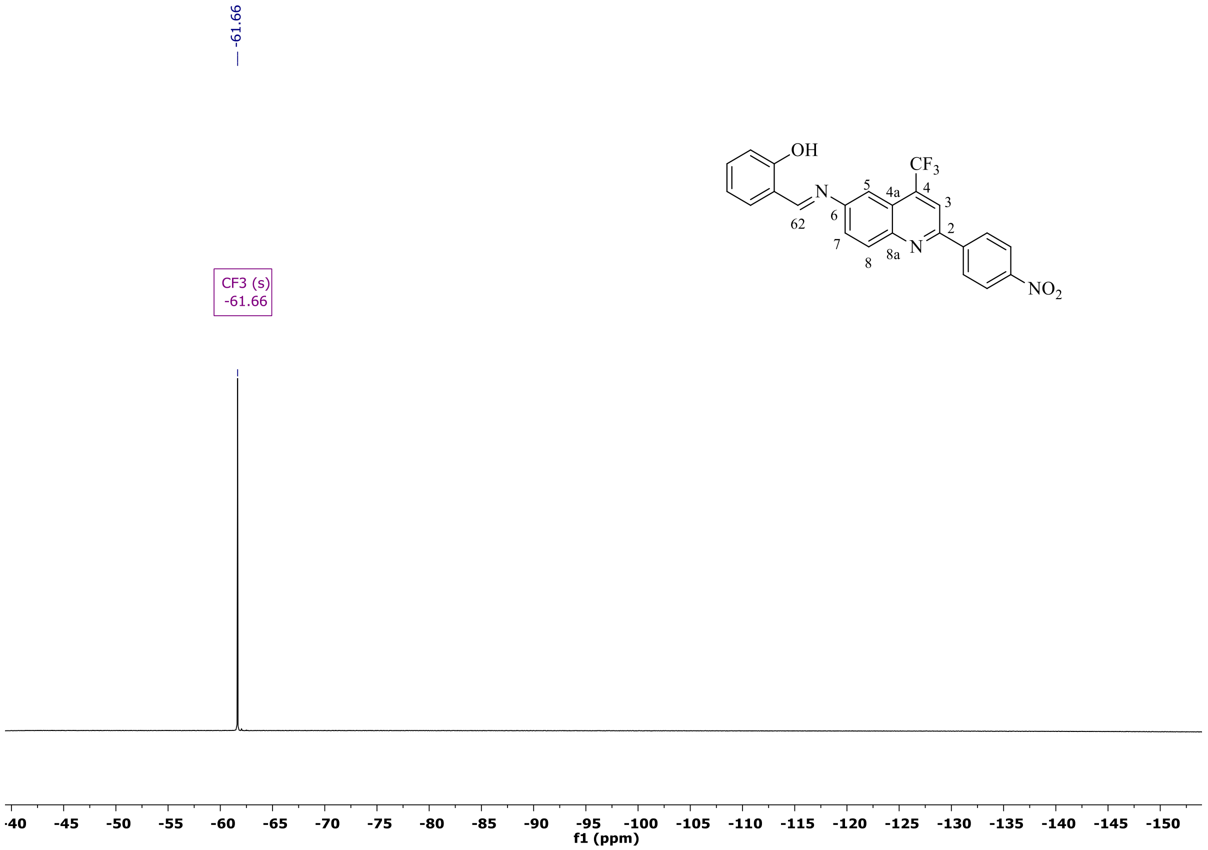

Figure S32: ^{19}F (565 MHz) NMR spectrum of **3ea** in $CDCl_3$.

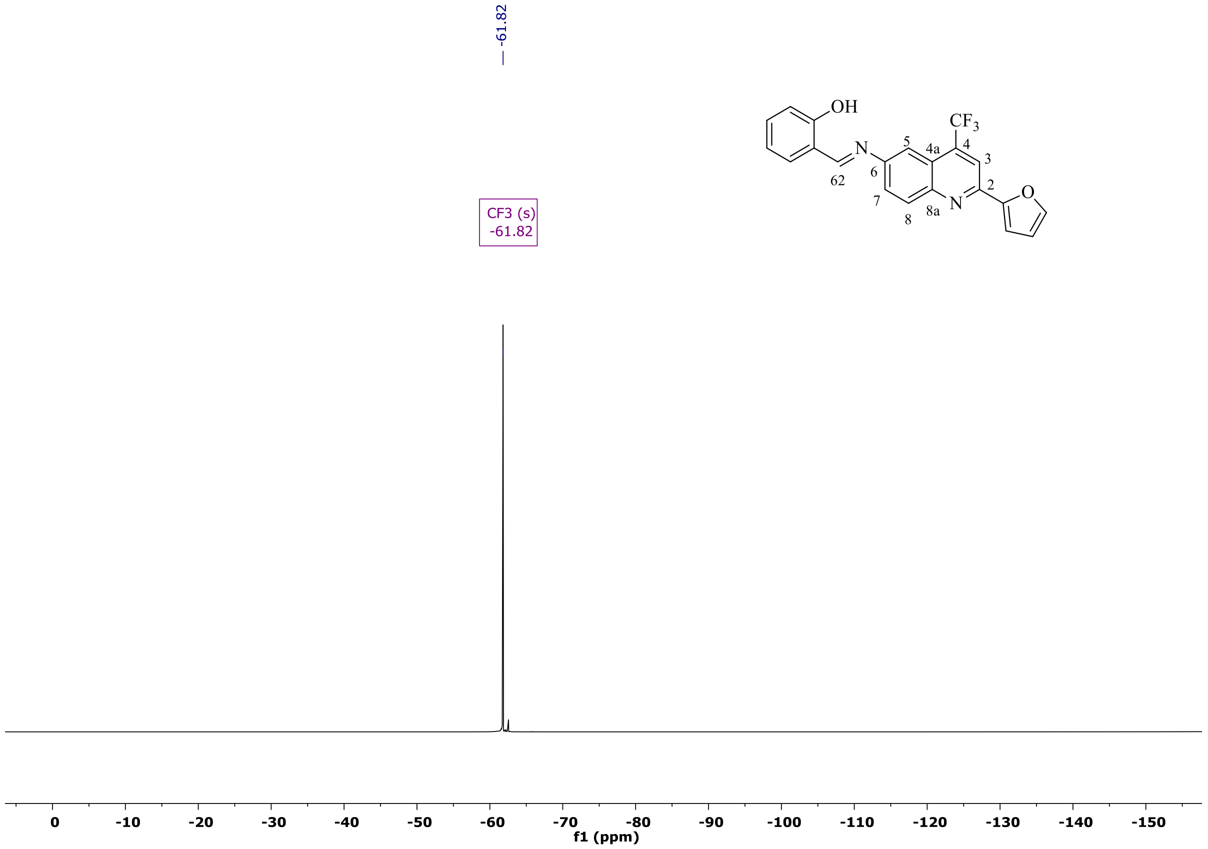

Figure S33: ^{19}F (565 MHz) NMR spectrum of **3fa** in $CDCl_3$.

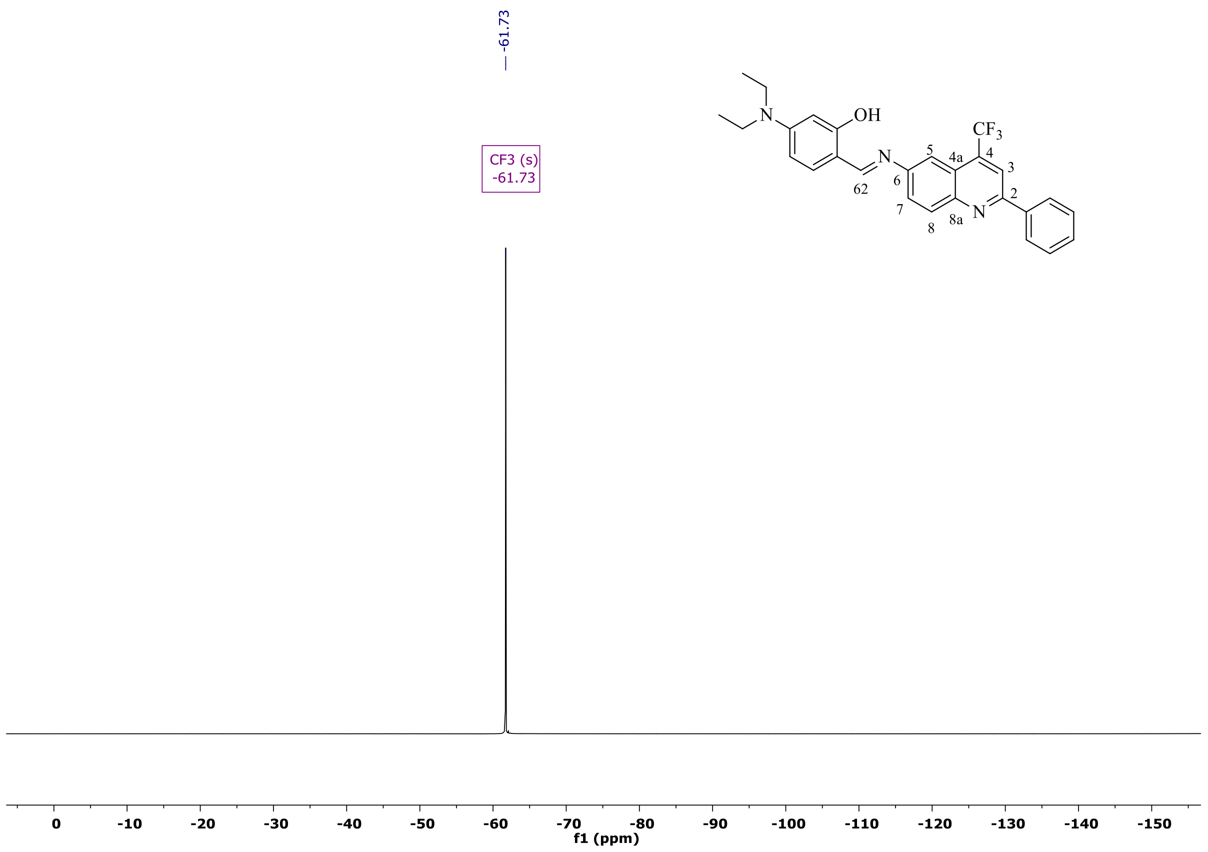

Figure S34: ^{19}F (565 MHz) NMR spectrum of **3bb** in $CDCl_3$.

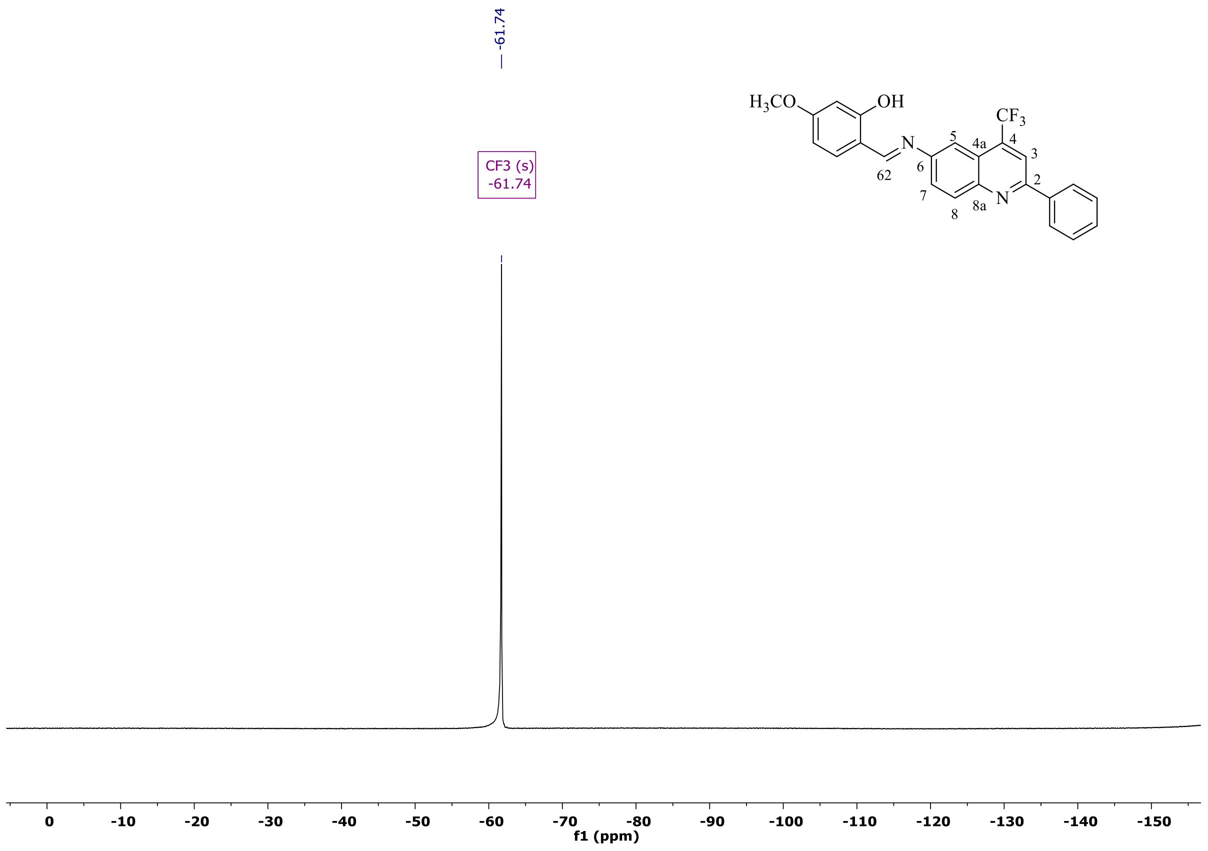

Figure S35: ^{19}F (565 MHz) NMR spectrum of **3bc** in $CDCl_3$.

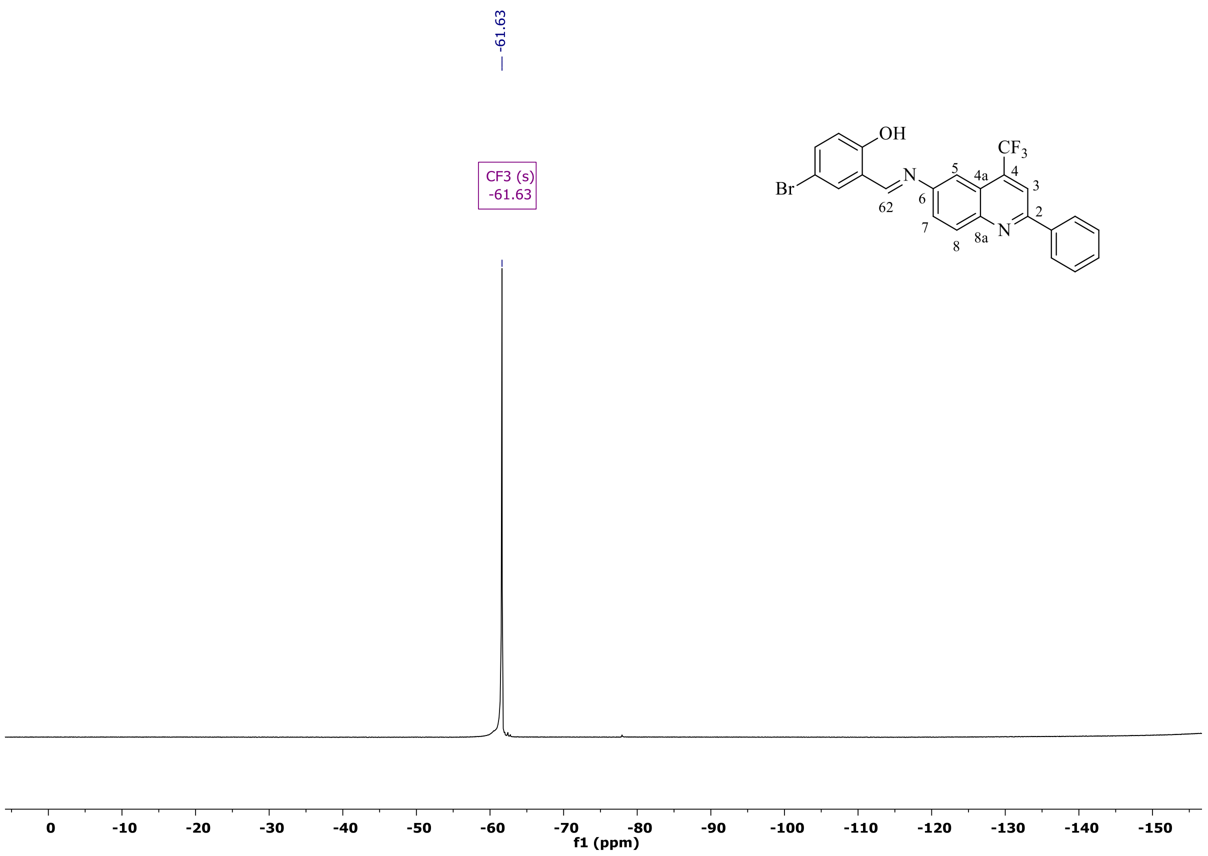

Figure S36: ^{19}F (565 MHz) NMR spectrum of **3bd** in $CDCl_3$.

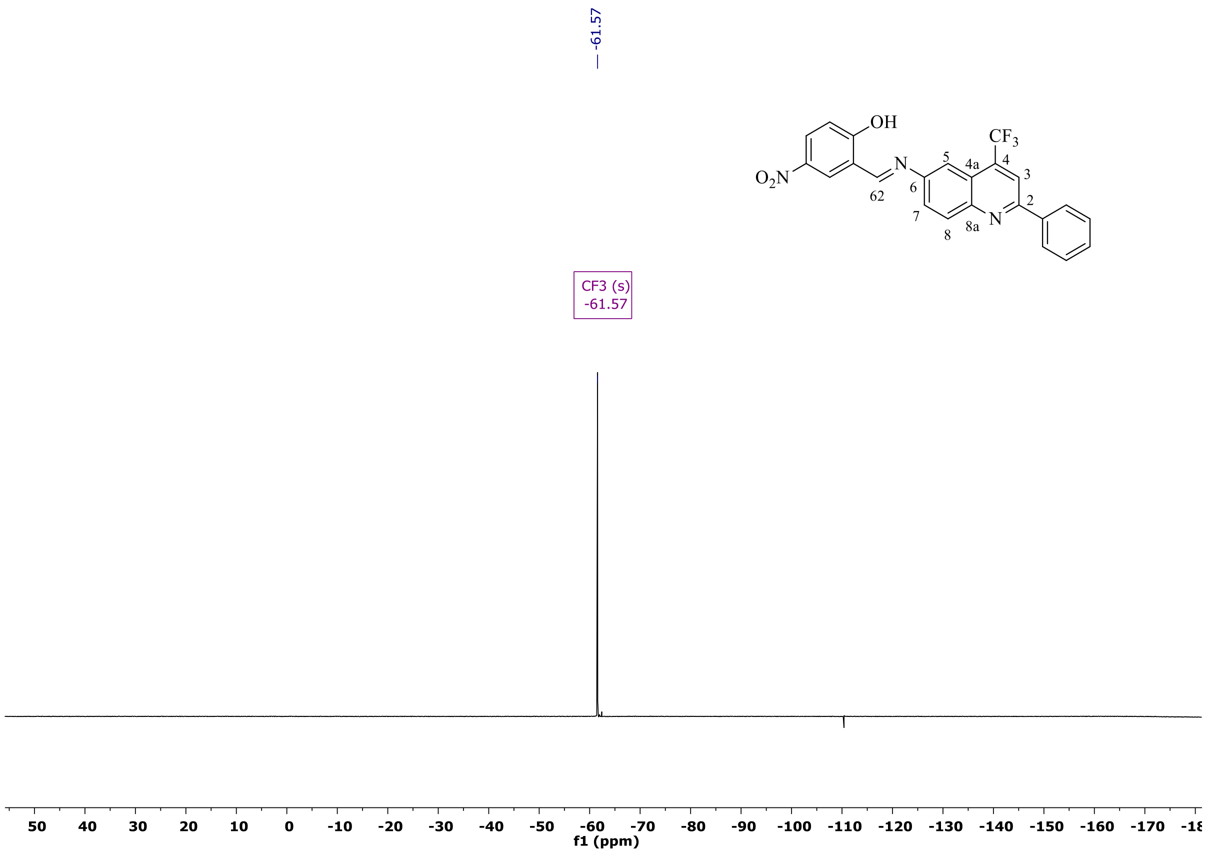

Figure S37: ^{19}F (565 MHz) NMR spectrum of **3be** in $CDCl_3$.

5. FTIR Spectra

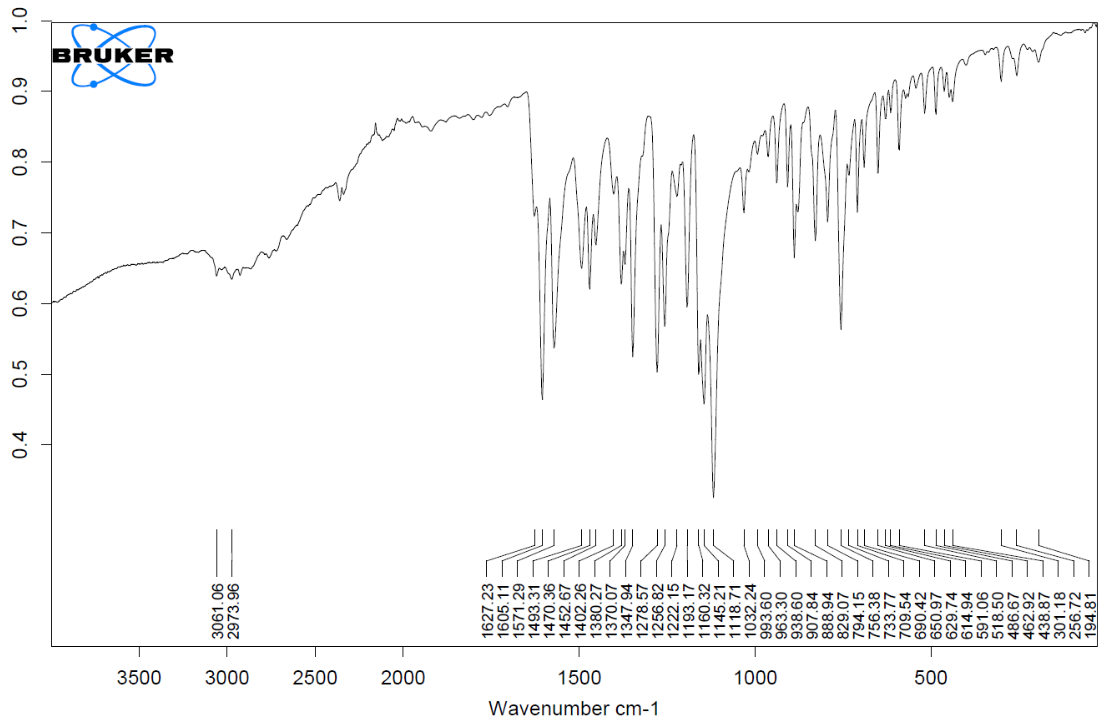

Figure S38: FTIR spectrum of compound **3aa**.

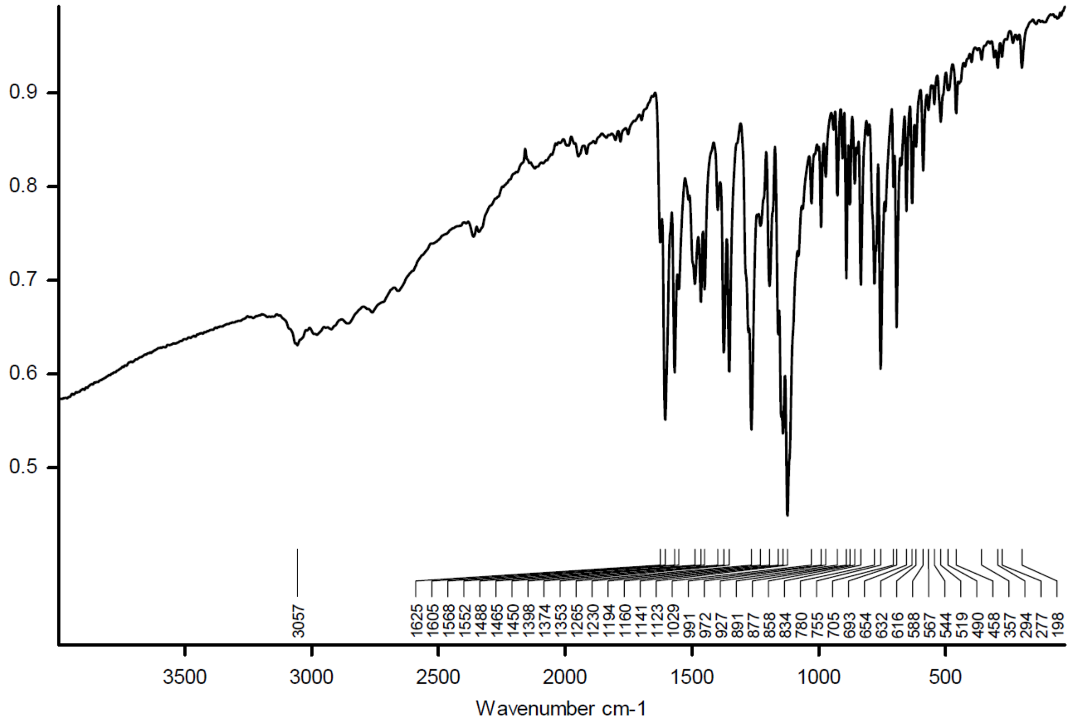

Figure S39: FTIR spectrum of compound **3ba**.

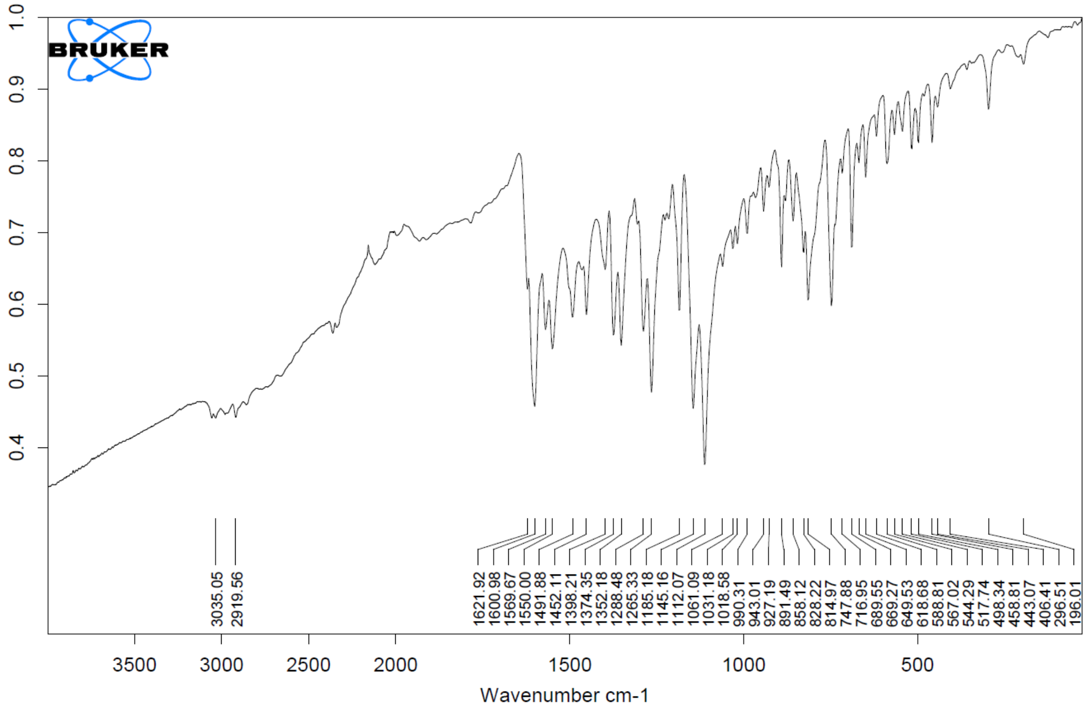

Figure S40: FTIR spectrum of compound **3ca**.

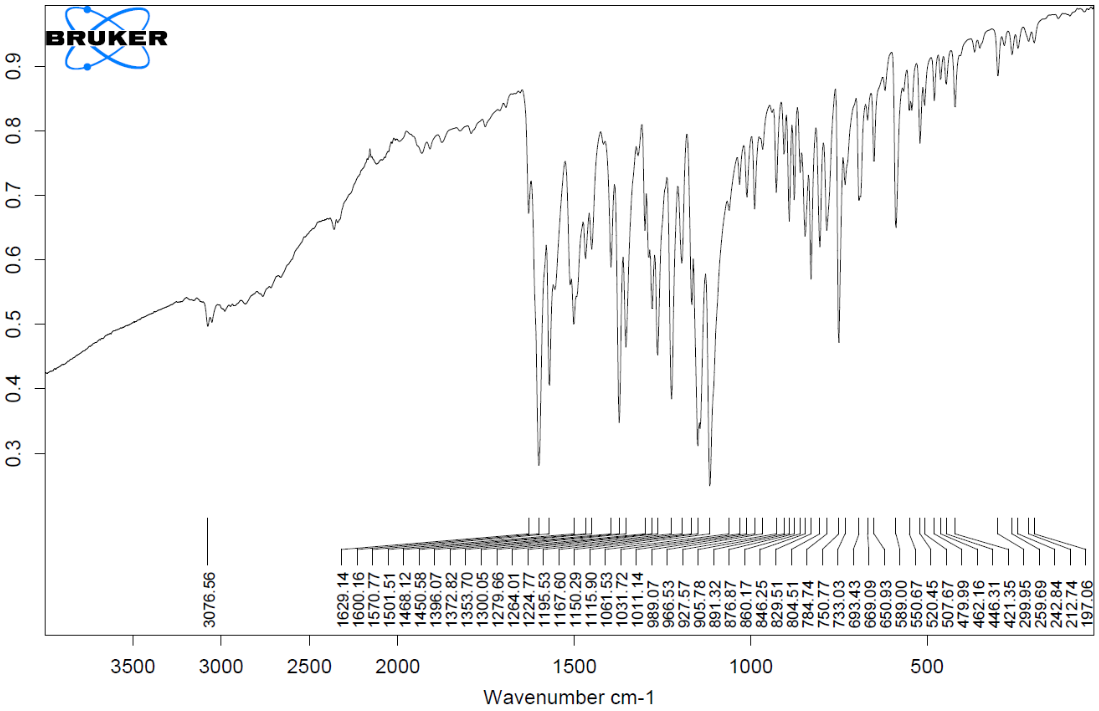

Figure S41: FTIR spectrum of compound **3da**.

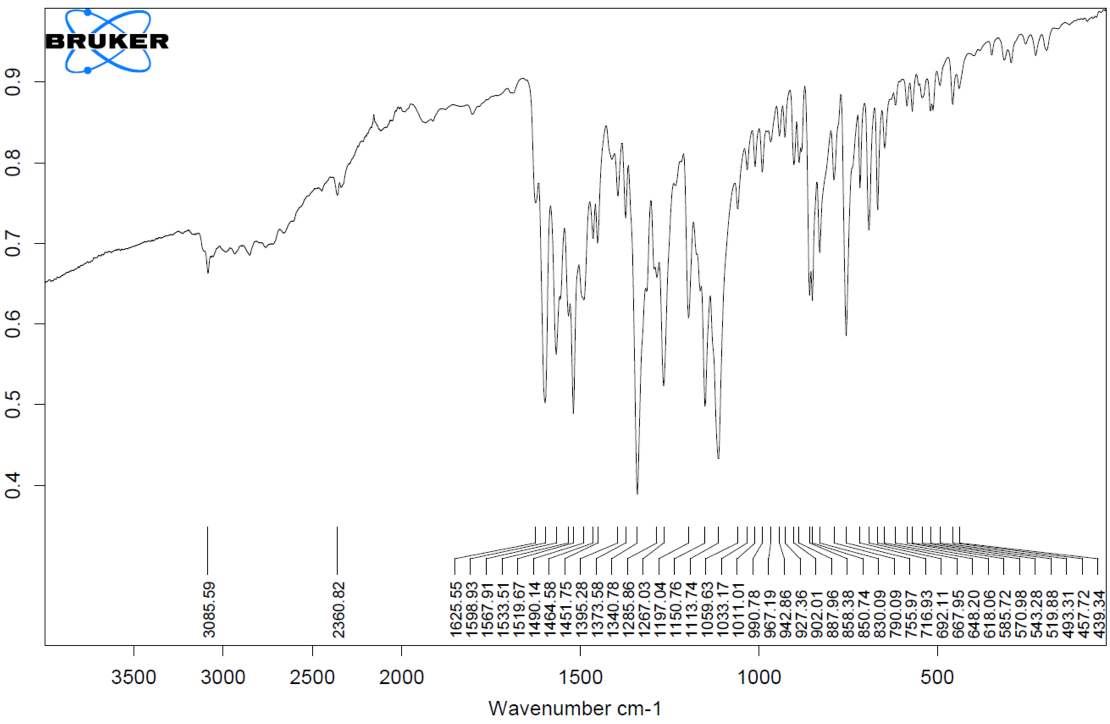

Figure S42: FTIR spectrum of compound **3ea**.

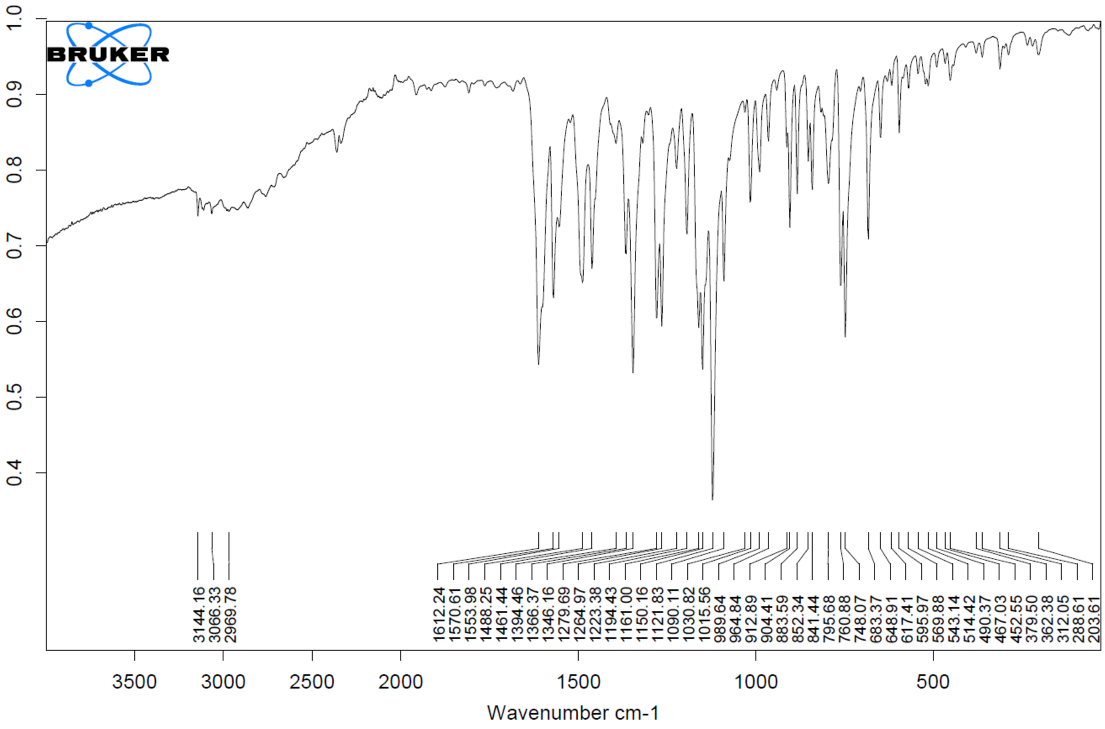

Figure S43: FTIR spectrum of compound **3fa**.

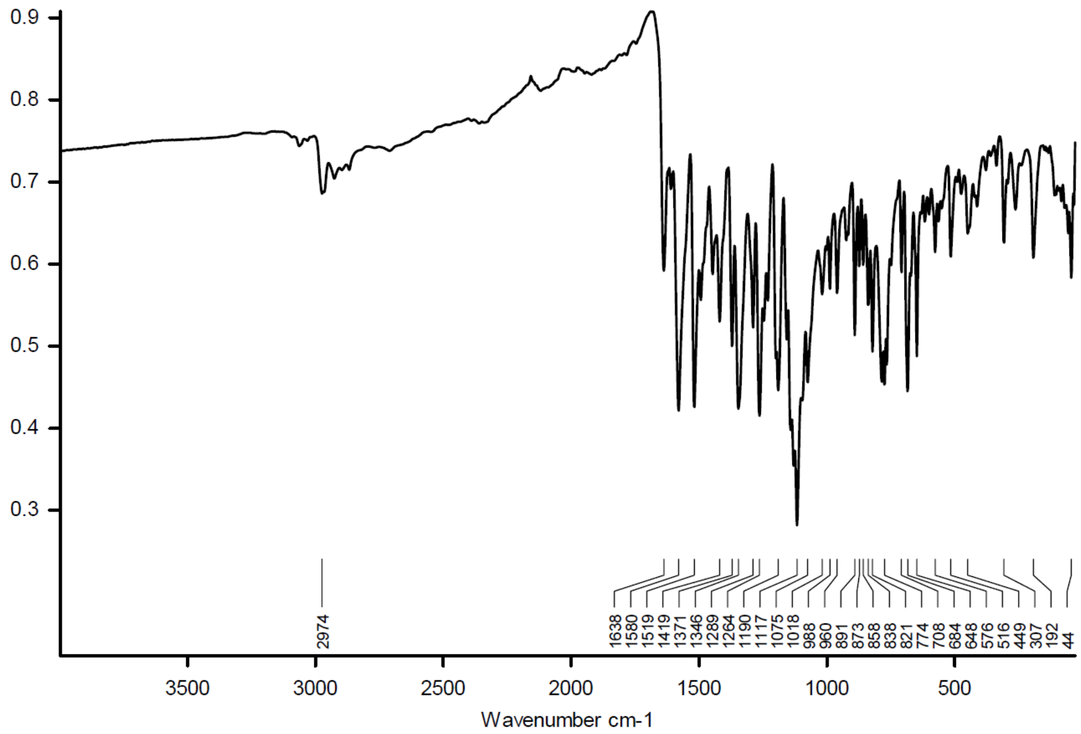

Figure S44: FTIR spectrum of compound **3bb**.

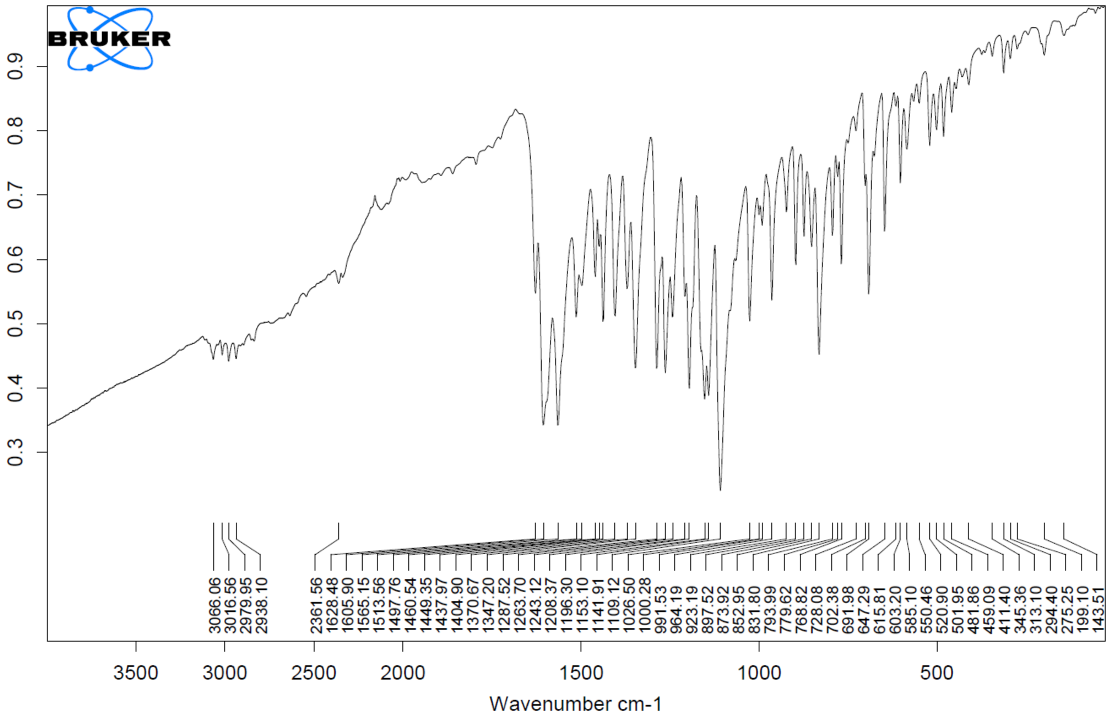

Figure S45: FTIR spectrum of compound **3bc**.

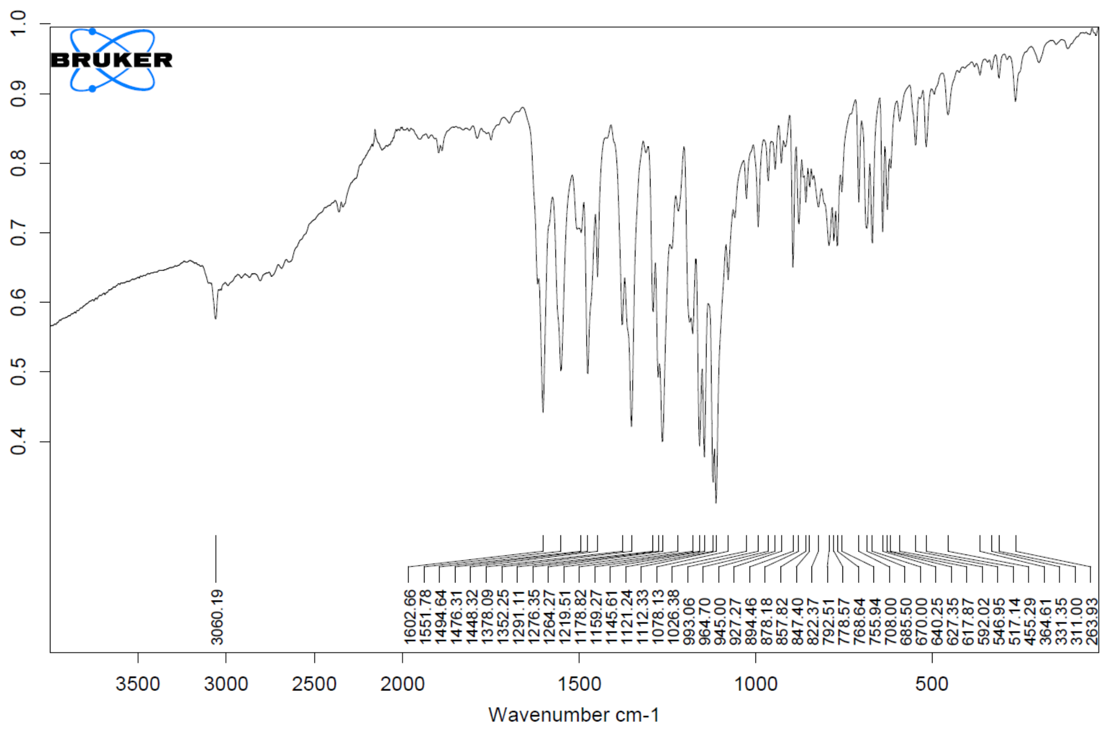

Figure S46: FTIR spectrum of compound **3bd**.

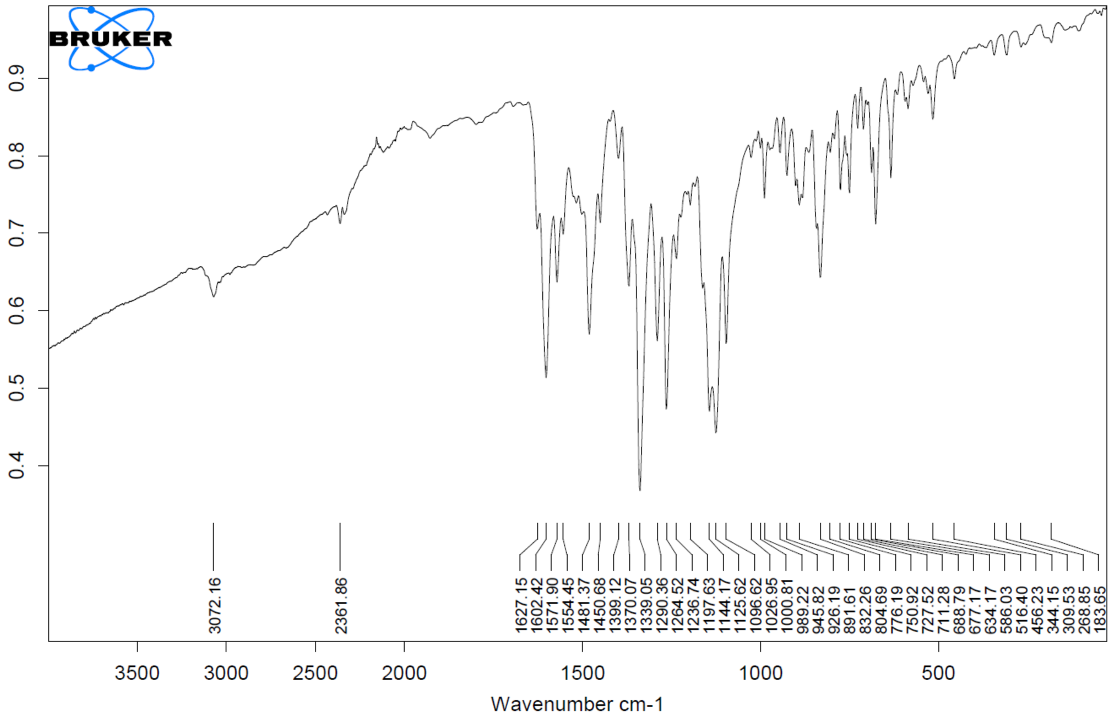

Figure S47: FTIR spectrum of compound **3be**.

References

(1) Farrugia, L. J. *J. Appl. Crystallogr.* **2012**, *45* (4), 849–854. doi:10.1107/S0021889812029111

(2) Sheldrick, G. M. *Acta Crystallogr. Sect. A Found. Crystallogr.* **2008**, *64* (1), 112–122. doi:10.1107/S0108767307043930

(3) Basso, G.; Cargnelutti, J. F.; Oliveira, A. L.; Acunha, T. V.; Weiblen, R.; Flores, E. F.; Iglesias, B. A. *J. Porphyr. Phthalocyanines* **2019**, *23* (9), 1041–1046. doi:10.1142/S1088424619500767

(4) Guterres, K. B.; Rossi, G. G.; Menezes, L. B.; Anraku de Campos, M. M.; Iglesias, B. A. *Tuberculosis* **2019**, *117* (May), 45–51. doi:10.1016/j.tube.2019.06.001

(5) Silveira, C. H. da; Vieceli, V.; Clerici, D. J.; Santos, R. C. V.; Iglesias, B. A. *Photodiagnosis Photodyn. Ther.* **2020**, *31* (June), 101920. doi:10.1016/j.pdpdt.2020.101920

(6) Pivetta, R. C.; Auras, B. L.; Souza, B. de; Neves, A.; Nunes, F. S.; Cocca, L. H. Z.; Boni, L. De; Iglesias, B. A. *J. Photochem. Photobiol. A Chem.* **2017**, *332*, 306–315. doi:10.1016/j.jphotochem.2016.09.008

(7) Tanielian, C.; Golder, L.; Wolff, C. *J. Photochem.* **1984**, *25* (2–4), 117–125. doi:10.1016/0047-2670(84)87016-1
